# Connecting single-nucleotide polymorphisms, glycosylation status, and interactions of plasma serine protease inhibitors

**DOI:** 10.1016/j.chempr.2022.11.018

**Published:** 2023-03-09

**Authors:** Di Wu, Manman Guo, Carol V. Robinson

**Affiliations:** 1 Department of Chemistry, University of Oxford, Oxford OX1 3QZ, UK; 2 Kavli Institute for Nanoscience Discovery, University of Oxford, Oxford OX1 3QU, UK; 3 Botnar Research Centre, NIHR Biomedical Research Unit Oxford, Nuffield Department of Musculoskeletal Sciences, University of Oxford, Oxford OX3 7LD, UK

**Keywords:** proteoform, single-nucleotide polymorphism, glycosylation, serine protease inhibitors, interaction

## Abstract

Understanding the combined impacts of genetic variances and post-translational modifications requires new approaches. Here, we delineate proteoforms of plasma serine protease inhibitors and relate specific proteoforms to their interactions in complexes through the use of native mass spectrometry (MS). First, we dissect the proteoform repertoire of an acute-phase plasma protein, serine protease inhibitor A1 (SERPINA1), resolving four SERPINA1 variants (M1V, M1A, M2, and M3) with common single-nucleotide polymorphisms (SNPs). Investigating the glycosylation status of these variants and their ability to form complexes with a serine protease, elastase, we find that fucosylation stabilizes the interaction of the SERPINA1 M1V variant through its core fucosylation on Asn271. In contrast, antennary fucosylation on Asn271 destabilizes SERPINA1-elastase interactions. We unveil the same opposing effects of core and antennary fucosylation on SERPINA3 interactions with chymotrypsin. Together, our native MS results highlight the modulating effects of fucosylation with different linkages on glycoprotein interactions.

## Highlights

• Native mass spectrometry uncovers proteoform and SNP-specific protein interactions
• Core and antennary fucosylation play opposing roles in SERPIN interactions
• Core fucosylation and E400D mutation cooperatively modulates SERPINA1 interactions

## The bigger picture

The most common type of genetic variation among people is single-nucleotide polymorphisms (SNPs). Occurring throughout a person’s DNA, SNPs can influence modifications on proteins (post-translational modifications [PTMs]). The combination of genetic variance and protein modification results in myriad variations. Understanding the combined impact of these two variations on protein activity and function is challenging.

Selecting serine proteases due to their roles in diverse physiological processes from blood coagulation to immunity, researchers considered how their activity is regulated by binding to inhibitory proteins (serine protease inhibitors [SERPINs]). Maintaining SERPIN-protease complexes intact and characterizing their PTMs/SNPs required high-resolution mass spectrometry. By developing such an approach, the authors revealed synergistic effects of SNPs and PTMs on SERPIN-protease interactions. More broadly, this approach connects genomic discovery with protein function.

## Graphical abstract

**Figure:**
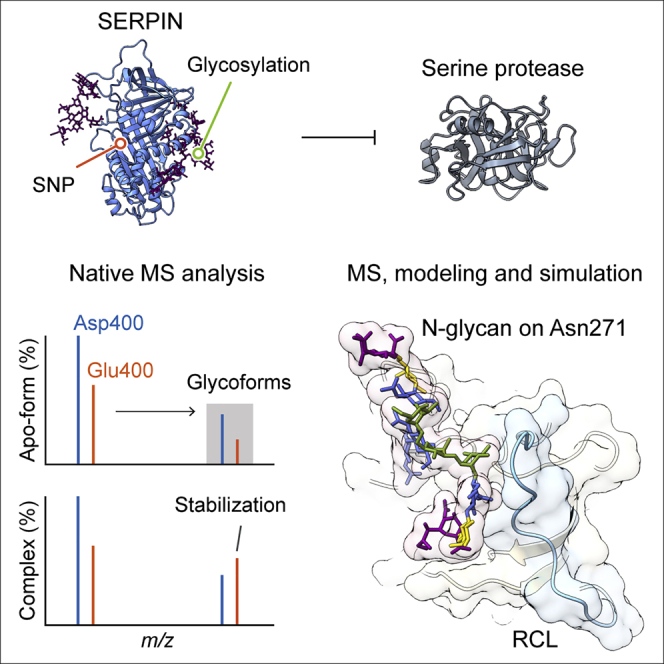

## Teaser

Understanding the combined effects of single-nucleotide polymorphisms (SNPs) and post-translational modifications (PTMs) on protein interactions is challenging. Wu et al. developed and applied a high-resolution native mass spectrometry (MS) approach to monitor individual SNPs and glycoforms on serine protease inhibitors (SERPINs) in complexes with proteases. To do this, they combined MS, modeling, and molecular dynamics simulations to relate site-specific glycan heterogeneity to SERPIN-protease interactions.

## Introduction

Proteoforms are distinct variants of protein molecules, derived from combinations of individual genetic variances, translational, and post-translational modifications (PTMs).^1^ Several diseases are driven by aberrant expression of these proteoforms making it imperative to understand how proteoforms regulate protein interactions and influence downstream signaling events. Recent genomics and proteomics studies discovered thousands of disease-related proteoforms and informed the importance of proteoforms in personalized medicine and oncology.^2^^,^^3^ A wealth of information exists for disease-related proteoforms in human blood,^4^ and many plasma protein proteoforms have been implicated in the predisposition or onset of disease.^5^^,^^6^ However, missing to date is the link between aberrant proteoforms or SNPs, their glycosylation status and how these combine to regulate plasma protein interactions.

Human serine protease inhibitors (SERPINs) belong to a family of 37 evolutionarily conserved members that regulate the activity of over 180 serine proteases.^7^ Therefore, the interplay of SERPINs and serine proteases controls many biological processes, including immune responses, coagulation, and cell growth/maturation. Notably, the expression levels and PTMs of SERPINs serve as biomarkers for susceptibility and pathogenesis of lung cancer, hepatocellular carcinoma, and coronavirus disease 2019 (COVID-19).^6^^,^^8^^,^^9^ Moreover, recent genome-wide association studies (GWASs) demonstrated that nonsynonymous mutations of SERPINs are related to their expression levels, function, and metabolic interactome.^5^^,^^10^

A number of SERPINs are present in human plasma, and all are structurally similar with two main features: a five-stranded β-sheet A and a flexible reactive center loop (RCL) containing a protease cleavage site. The two most abundant plasma SERPINs, SERPINA1 (α1-antitrypsin) and SERPINA3 (α1-antichymotrypsin) are acute-phase proteins, upregulated during inflammation.^7^ SERPINA1 is the major endogenous inhibitor of elastase, a serine protease secreted by activated neutrophils to degrade foreign molecules and microorganisms.^11^ SERPINA1 is also the endogenous inhibitor of the transmembrane protease serine 2 (TMPRSS2), critical for spike protein shedding on the SARS-CoV-2 virion.^12^ SERPINA3 is an important regulator of the renin-angiotensin system and neutralizes mast cell chymase and neutrophil cathepsin G activities.^13^

More than a hundred SERPINA1 variants have been identified, many of which are related to α1-antitrypsin deficiency (AATD), an autosomal-codominant disorder characterized by reduced protein levels in plasma. Conflicting reports exist however about whether other common variants are benign or pathogenic with these discrepancies arising from genomics, biochemical and biophysical observations.^5^^,^^10^^,^^14^^,^^15^ Genomics studies revealed associations of SNPs in SERPINA1 to specific diseases. Nevertheless, SERPINA1 has three heterogeneous covalently linked N-glycans, with differences in occupancy, composition, linkage, and structure that are anticipated to fine-tune protein structure and function.^16^ The apparent activity and function of endogenous SERPINA1, containing all proteoforms, is therefore likely highly variable. While recombinant protein expression eliminates this variability, proteins expressed in immortalized cell lines are often different from their endogenous counterparts derived from human plasma/tissue.^17^ Therefore, capturing proteoform-specific interactions of endogenous proteins from human plasma/tissue is critical in connecting genomic discoveries to protein function.

Mass spectrometry (MS)-based proteomics, involving sequencing of either digested or intact proteins, plays an important role in proteoform discovery, particularly in identifying novel PTMs^18^ and unveiling the regulation of biological systems.^19^ Proteomics is not yet capable of directly informing proteoform-specific interactions.^20^ Recently, native MS has demonstrated great promise in defining the extent of proteoforms within protein complexes.^21^ Here, we investigated how SNPs and PTMs synergize to impact SERPIN interactions using a native MS approach.

## Results

### Resolving SERPINA1 proteoforms by high-resolution native MS

**Figure 1:**
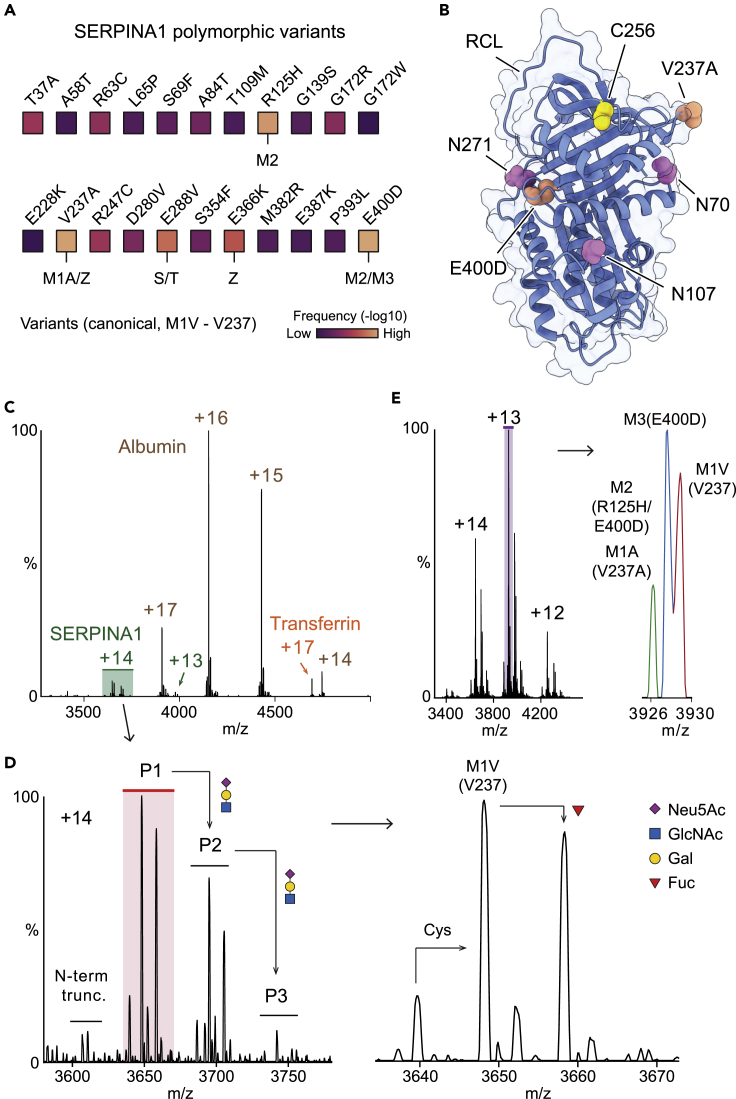
Probing SERPINA1 heterogeneity using native MS (A) The frequency of common SERPINA1 polymorphisms. The M1A, M1V, M2, and M3 variants are the four most abundant isoforms encoded by the wild-type SERPINA1 sequence. S, T, and Z are pathogenic variants related to alpha-1 antitrypsin deficiency. (B) SERPINA1 carries three N-glycosylation sites on Asn70, Asn107, and Asn271 (highlighted in pink) and one cysteinylation site on C256 (highlighted in yellow). Two mutations (Val237Ala and Glu400Asp) are highlighted in orange. (C) Native MS of an undepleted human plasma sample from an individual donor. Albumin, SERPINA1, and transferrin peaks are labeled with the corresponding charge states. The albumin peaks with charge state +17 overlap with the SERPINA1 peaks with charge states +13. (D) SERPINA1 peaks with charge state +14. Three main peak series, namely P1, P2, and P3 are observed that correspond to additions of Neu5Ac-Gal-GlcNAc units. Within those series, further heterogeneity is assigned to cysteinylation (Cys) and fucosylation. An N-terminal truncated form (N-term trunc.) missing Asp25 to Gly29 was also detected. (E) Native mass spectrum of SERPINA1 from pooled plasma with peaks assigned to single amino acid mutations. Three peaks are annotated to M2/M1A, M3, and M1V variants, respectively. The mass spectra in (C) and (D) were acquired with R values of 30,000 and R = 25,000 for (E).

We began our investigation using human plasma directly from a single donor on a modified Orbitrap Eclipse mass spectrometer.^22^ Since SERPINA1 is an abundant plasma glycoprotein and the *SERPINA1* gene is highly polymorphic, we anticipated detection of one or more of the twenty frequently observed SNPs in the coding exon region. The M1V (canonical sequence), M1A (Ala237), M2 (His125/Asp400), and M3 (Asp400) are the four most common variants, encoded by wild-type *SERPINA1* sequences (Figure 1A). SERPINA1 also carries three N-glycosylation sites at Asn70, Asn107, and Asn271, and a cysteinylation site at C256 (Figure 1B). The serine protease cleavage site (the cleavage occurs between Met382 and Ser383) is located on the RCL (Gly368 to Lys392). The native mass spectrum of this donor plasma is dominated by serum albumin with represents >50% of the plasma protein content (Figure 1C). Its different charge state peaks overlap those of other proteins, leading to ambiguous mass assignments for other plasma proteins. Therefore, we applied high-resolution conditions on the Orbitrap Eclipse platform (resolving power of 500,000 at *m*/*z* 200) to assign SERPINA1 peaks using a single charge state (Figure S1). The spectrum revealed the SERPINA1 proteoforms at the intact protein level, including the genetic variant (M1V), N-terminal truncation status, glycosylation heterogeneity (N-glycan branching and fucosylation), as well as the stoichiometry of cysteinylation (Figure 1D), in agreement with recent MS studies of SERPINA1.^23^ N-glycan branching events (addition of a trisaccharide: Neu5Ac-Gal-GlcNAc; Neu5Ac, *N*-acetylneuraminic acid; Gal, galactose; GlcNAc, *N*-acetylglucosamine) account for the major heterogeneity of the SERPINA1 peaks spanning the molecular weight range from 51 to 53 kD.

To eliminate the interference from serum albumin and transferrin, and fully resolve SERPINA1 and SERPINA1-elastase complexes, we then analyzed isolated SERPINA1 from pooled human plasma. The native mass spectrum of SERPINA1 from pooled human plasma shows similar PTM patterns to the non-purified SERPINA1 from the individual donor (cysteinylation, glycosylation, and N-terminal truncation) (Figure S2), suggesting no bias to specific proteoforms was introduced during purification. Importantly, the four most common variants, M2 (His125/Asp400), M1A (Ala237), M3 (Asp400), and M1V (canonical sequence) could be observed with isotopic resolution under high-resolution conditions on the Orbitrap Eclipse platform (Figures 1E and S3). We further confirmed the presence of these variants by native top-down MS analysis (Figure S4) and MS-based bottom-up proteomics (Figure S5). Importantly this confirmation, together with the relative abundance determined from native MS, enables us to compare the protease binding events of these variants simultaneously and directly.

### Probing proteoform-specific regulation of SERPINA1-elastase complexes

**Figure 2:**
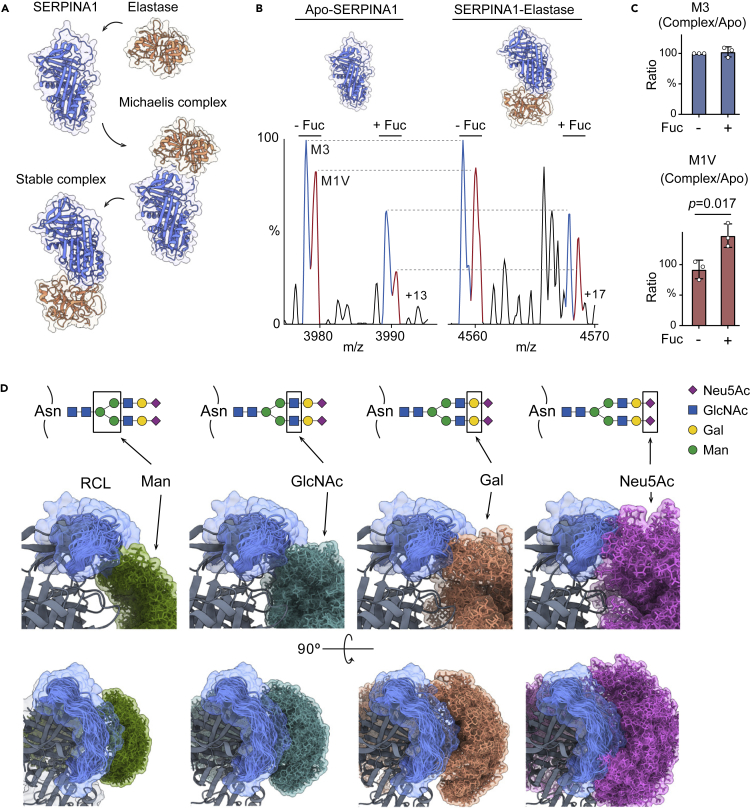
Analysis of SERPINA1-elastase complexes using native MS and MD simulations (A) Illustrative mechanism of the SERPINA1-elastase interaction. Elastase recognizes the RCL and forms a Michaelis-Menten complex with SERPINA1. After hydrolysis of the enzymatic cleavage sites, elastase and SERPINA1 form a stable complex. (B) Mass spectra of the SERPINA1 variants M3 and M1V in apo forms and in complex with elastase. The N-glycan composition and variants are labeled. (C) Bar graphs of the ratio of the SERPINA1-elastase complex to apo-SERPINA1, with and without fucosylation. Bars show mean ± standard deviation with dots from three independent experiments. A Student’s t test was performed to calculate the p value. (D) Stereoview of the superimposed conformers of four different monosaccharide residues on Asn271 and the RCL from the 150 ns MD simulation trajectory. The snapshots of the monosaccharide residues and RCL conformations are extracted (one frame ns^−1^) and overlayed.

SERPINA1 traps elastase by a two-step process (Figure 2A). First, it captures elastase and forms a reversible Michaelis-Menten complex, subsequently, it is proteolytically cleaved and undergoes a large conformational change to form a stable complex with elastase. Several missense mutations and glycoforms in SERPINA1 reduce this protease inhibitory activity by unknown mechanisms.^9^^,^^24^^,^^25^ To explore how these distinct proteoforms regulate the SERPINA1-elastase interactions, we first analyzed SERPINA1 mixed with sub-stoichiometric quantities of elastase, using our modified Eclipse mass spectrometer. The resulting spectrum shows peaks assigned to both apo-SERPINA1 and the SERPINA1-elastase complex (Figure S6A). Unexpectedly, high resolution was maintained within these ∼80 kDa SERPINA1-elastase complexes at *m*/*z* > 4,500 enabling us to distinguish the single amino acid difference between M1V (canonical sequence) and M3 (Glu400Asp). We compared the relative abundances of peaks assigned to the SERPINA1-elastase complex with two bi-antennary and one tri-antennary N-glycans with the corresponding peaks of apo-SERPINA1 (Figure 2B). Interestingly, mono-fucosylation stabilizes the M1V variant-elastase complex but has no impact on the M3 variant-elastase complex (Figure 2C). Therefore, we hypothesize that this single amino acid difference (Glu400Asp) may cause variant-specific regulation through fucosylation.

To investigate the effects of this possible SNP regulation, we compared the structural differences between M1V and M3 variants (Figure S6B). The Glu400 in M1V (corresponding to Asp400 in M3) is proximal to the glycosylation site in the structure (Asn271) such that the side chain of Glu400 can form hydrogen bonds with the side chains of Thr126, Thr379, Ser381, and Asn402. The Glu400Asp mutation introduces a shorter negatively charged side chain to the M3 variant, which might affect its hydrogen bonding to other amino acids (Figures S6C and S6D). To investigate this, we performed molecular dynamics (MDs) simulations of the M1V and M3 variants. We found that while the Glu400Asp mutation does not impact protein backbone stability or conformation (Figures S6E and S6F), it does reduce hydrogen-bonding interactions to side chains (Figure S6G). As a consequence, the flexible side chains of Thr126, Thr379, and Asn402 might be anticipated to affect the N-glycan dynamics of the proximal Asn271.

We next investigated whether or not the N-glycans on SERPINA1 can influence the RCL, which is critical for activity (Figure 1B). We performed MD simulation of a fully glycosylated SERPINA1 (M1V variant) with three bi-antennary N-glycans with α2-6 sialylation on Asn70, Asn107, and Asn271 (Figures S7A and S7B). We then extracted snapshots of the N-glycans and RCL conformations over the simulation trajectory (one frame per 1 ns) and overlayed those conformations (Figure S7C). We found only the flexible N-glycan on Asn271 is proximal and able to interact with the RCL. To probe further the N-glycan (Asn271)-RCL interactions, we then extracted and overlayed snapshots of the individual monosaccharide residues (Figure 2D). Notably, RCL can contact the antennary monosaccharide residues (α2-6 Neu5Ac and β1-4 Gal) rather than the core α1-3,6 Man and β1-2 GlcNAc residues. Therefore, we propose that the terminal monosaccharide residues, namely α2-6 Neu5Ac and/or β1-4 Gal on the Asn271 N-glycan interact with the RCL and may regulate SERPINA1-elastase binding. We further released the negatively charged Neu5Ac residues from SERPINA1 using neuraminidase treatment to investigate whether we can suppress fucosylation regulation. We probed the desialylated SERPINA1-elastase complexes using native MS (Figure S8). Interestingly, we found the mono-fucosylation stabilization effect on M1V variant is completely abolished after the removal of the terminal Neu5Ac residues. Together, these results suggest that mono-fucosylation regulates M1V variant, and the terminal Neu5Ac residues are essential for this regulation.

### Core fucosylation stabilizes SERPINA1 interactions with elastase

**Figure 3:**
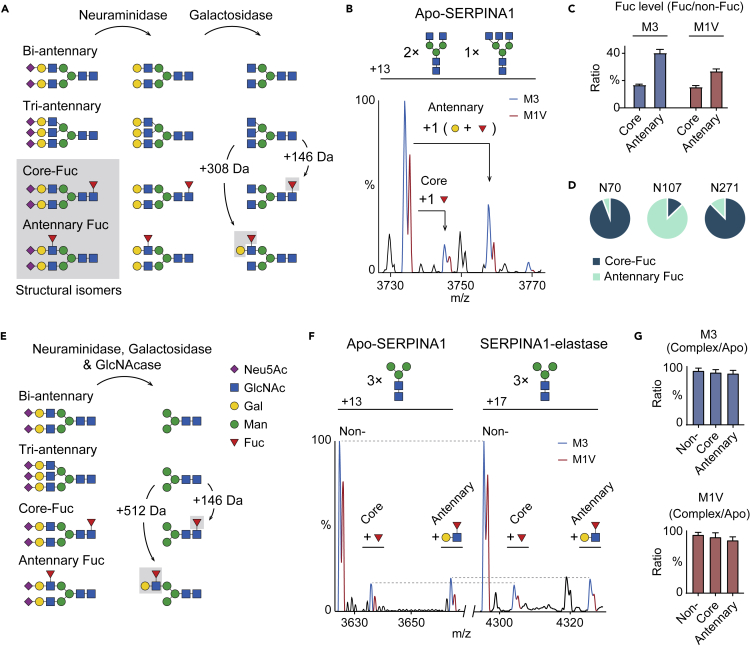
Analysis of core and antennary fucosylation on SERPINA1 and SERPINA1-elastase complexes (A) Schematic illustration of neuraminidase and galactosidase digestion of N-glycans. Core-fucosylated and antennary fucosylated N-glycans are structural isomers. After double exoglycosidase digestion, the isomeric fucosylated N-glycans are transformed into two different structures with a mass difference of 162 Da (a galactose residue). (B) Native mass spectrum of SERPINA1 digested with neuraminidase and galacosidase. (C) Core- and antennary fucosylation levels of double exoglycosidase digested M3 and M1V variants. Bars show mean ± standard deviation. (D) Quantification of site-specific core- and antennary fucosylation on Asn70, Asn107, and Asn271. (E) Schematic illustration of triple exoglycosidase digestion for N-glycans using neuraminidase, galactosidase and GlcNAcase. The triple exoglycosidase digestion removes all non-fucosylated N-glycan antennae. (F) Native mass spectra of triple exoglycosidase treated apo-SERPINA1 and SERPINA1-elastase complexes. (G) Bar graphs of the ratios of the triple exoglycosidase treated SERPINA1 (M3 and M1V variants)-elastase complex to apo-SERPINA1. The ratios for non-, core- and antennary fucosylated forms are plotted. Bars show mean ± standard deviation.

For SERPINA1 fucosylation, the fucose residue can be either α1-6 linked to the innermost GlcNAc residue (core fucosylation) or α1-3/4 linked to the branched GlcNAc residue (antennary fucosylation) (Figure 3A).^26^ However, the core- and antennary fucosylated N-glycans are isomers that could not be distinguished by native MS. Therefore, we applied a double exoglycosidase treatment strategy to release Neu5Ac and Gal residues using neuraminidase and galactosidase (Figure 3A).^27^ Briefly, either core- or antennary fucose residues do not have any impact on neuraminidase treatment. Nevertheless, antennary fucosylation sterically inhibits galactosidase activity and leaves one terminal galactose residue after digestion. This treatment transforms the fucosylated isomers into two smaller glycans with a difference of one galactose residue (162 Da). Following this treatment, we analyzed the double exoglycosidase digested SERPINA1 with native MS. The spectrum shows that all peaks of the trimmed SERPINA1 move to a lower *m*/*z* region compared with untreated SERPINA1 (Figure S9). After annotating all major peaks, we confirmed that all antennary Neu5Ac and Gal residues were removed from SERPINA1. Furthermore, the spectrum shows the M1V and M3 variants carry both core- and antennary fucose residues (Figures 3B and 3C). The ratio of the mass spectral peaks shows that the both variants bear similar core-fucosylation levels.

Next, we performed glycoproteomics of SERPINA1 to examine the site-specific fucosylation level (Figure S10). All three N-glycosylation sites (Asn70, Asn107, and Asn271) can be either core- or antennary fucosylated (Figure 3D). Importantly, the fucosylation on Asn271 is dominated by core-fucosylated glycan (90%) with only 10% antennary fucosylation. Moreover, the antennary fucosylation is primarily present on Asn107, distal from RCL. A previous study revealed antennary and core fucosylation have different impacts on N-glycan conformation.^28^ Hence, we hypothesize that the core-fucosylation level on the M1V variant may stabilize the M1V-elastase complexes via changing the N-glycan conformations and/or dynamics on Asn271.

To test this hypothesis, we first attempted to release the core-fucose residue on SERPINA1 with α1-6 fucosidase digestion. No de-fucosylated species was observed since core-fucose residues on intact glycoproteins are typically shielded by the extended sugar chain. This steric hindrance largely reduces the α1-6 fucosidase activity to folded glycoproteins.^9^ Additionally, we found that the double exoglycosidase treatment of SERPINA1 generates an interfering GlcNAc adduct peak series, which overlaps with the fucosylated species in native MS analysis (Figure S11). To this end, we performed a triple exoglycosidase treatment to remove Neu5Ac, β1-4 Gal, and β1-2 GlcNAc residues using neuraminidase, galactosidase, and *N*-acetylglucosaminidase (GlcNAcase). This treatment completely removes antennary sugar residues on non-fucosylated glycans, leaving the trimannosyl pentasaccharide cores (Man_3_GlcNAc_2_) on SERPINA1 (Figure 3E). The core and antennary fucosylated glycans are transformed to the cores (Man_3_GlcNAc_2_) with a single core-fucose residue and a Galβ1-4(Fucα1-3/4)GlcNAc trisaccharide, respectively. We then analyzed the treated SERPINA1 with elastase to examine the extent of complex formation (Figure 3F). We found that there was no difference between the ratio of complexes for the two SNPs with core or antennary fucosylation. This allows us to conclude that core-fucose regulation is completely abolished when the outer arm residues (Neu5Ac-Gal-GlcNAc unit) are removed (Figure 3G). Moreover, the antennary fucosylation (Galβ1-4(Fucα1-3/4)GlcNAc unit), which primarily occurs on Asn107 does not play any function on SERPINA1-elastase interactions.

We further investigated whether N-glycan branching, in terms of additional Neu5Ac-Gal-GlcNAc units can modulate SERPINA1-elastase interactions. We compared the relative abundances of the SERPINA1-elastase complex with two bi- and one tri- antennary N-glycans with the complexes with three bi- antennary N-glycans (Figure S12A). However, we found that the additional Neu5Ac-Gal-GlcNAc unit on SERPINA1 does not significantly impact SERPINA1-elastase interactions (Figure S12B). We then analyzed the distribution of bi- and tri-antennary N-glycans across the three N-glycosylation sites in SERPINA1. We found that tri-antennary N-glycan is principally distributed on Asn107 (Figure S12C). Therefore, the additional Neu5Ac-Gal-GlcNAc unit on Asn107, which is distal to the RCL, does not have any significant effect on SERPINA1 interactions.

Collectively, we have shown that the N-glycan on Asn271 is critical for regulating SERPINA1-elastase binding via the antennary sugar moieties whereas additional Neu5Ac-Gal-GlcNAc units have little impact. Moreover, we attributed the stabilization effect on the M1V variant-elastase complex to core fucosylation on Asn271. However, the antennary fucosylation level on SERPINA1 Asn271 is relatively low. We, therefore selected another SERPIN, SERPINA3, which has higher levels of antennary fucosylation than SERPINA1, to understand further the regulation of SERPIN-protease interactions.

### Core and antennary fucosylation play different roles in SERPINA3 interactions

**Figure 4:**
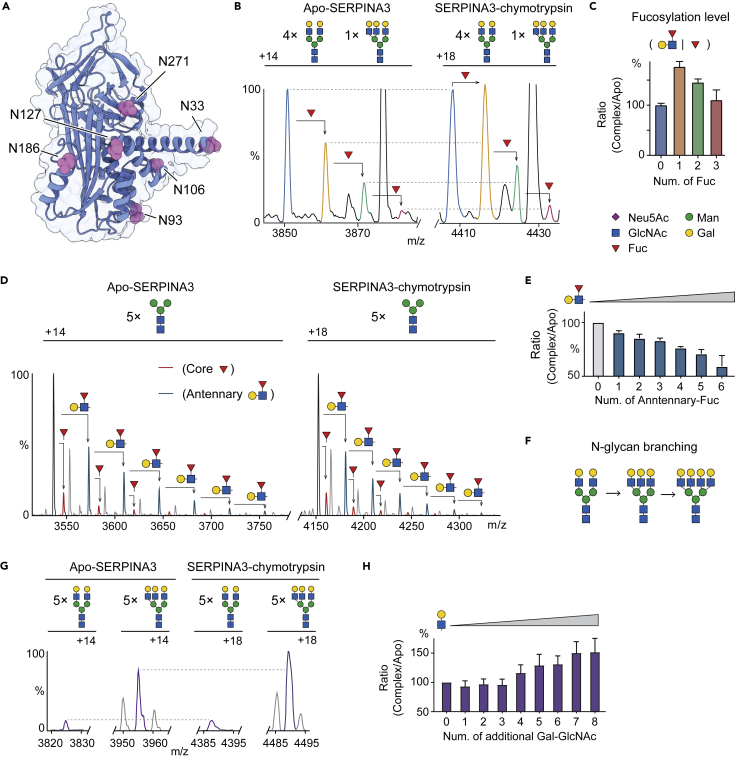
Probing N-glycan heterogeneity on SERPINA3-chymotrypsin complexes using native MS analysis (A) Structure of SERPINA3. Six potential N-glycosylation sites, including Asn33, Asn93, Asn106, Asn127, Asn186, and Asn271 are highlighted in pink. (B and C) (B) Native MS analysis of the desialylated SERPINA3-chymotrypsin complexes. Native mass spectra show the fucosylation status (0 to 3 fucose residues) of apo-form SERPINA3 and SERPINA3-chymotrypsin complexes. The ratios of their relative abundances are plotted as a bar graph in (C). Error bars represent the standard deviation of three individual replicates. (D) Native MS analysis of triple exoglycosidase treated SERPINA3-chymotrypsin complexes reveals the stoichiometry and relative abundances of core- and antennary fucosylation on apo-SERPINA3 and SERPINA3-chymotrypsin complexes. (E) The ratio shown in a bar graph of the triple exoglycosidase treated SERPINA3-chymotrypsin complex to apo-SERPINA3 with different numbers of antennary fucose residues. Bars show mean ± standard deviation from three independent experiments. (F) Illustration of N-glycan branching events on SERPINA3. (G and H) (G) Native MS analysis shows the N-glycan branching status on apo-SERPINA3 and SERPINA3-chymotrypsin complexes. The ratios of the relative abundances for the corresponding proteoforms (same numbers of additional Gal-GlcNAc units) are plotted as a bar graph in (H). Bars show mean ± standard deviation from three independent experiments.

SERPINA3 is structurally homologous to SERPINA1, but more highly glycosylated with six potential N-glycosylation sites (Asn93, Asn106, Asn127, Asn133, Asn186, and Asn271) (Figures 4A and S13A). In contrast to SERPINA1, SERPINA3 carries multiple fucose residues.^29^ This provides an opportunity to examine stoichiometric regulation via fucosylation. Similar to SERPINA1, SERPINA3 carries a complex type of N-glycan on Asn271, which is proximal to the RCL, such that it may regulate interactions with proteases (Figure S13B). To investigate this, we first analyzed desialylated SERPINA3 from human plasma and observed N-terminal truncated proteoforms, with highly branched N-glycans and fucosylation, in line with a previous native MS study (Figures S14A and S14B).^29^ We also found a minor peak series corresponding to the full-length proteoform and further confirmed this observation by MS-based proteomics (Figures S14B–S14D).^30^

SERPINA3 is the endogenous inhibitor for cathepsin G secreted by neutrophils, chymase derived from mast cells and the angiotensin-converting enzyme.^31^ Since both human chymase and cathepsin G are glycosylated, native MS of SERPINA3-protease complexes would be overly complicated. We therefore selected bovine chymotrypsin, a non-glycosylated structural homolog of human chymase and cathepsin G (Figure S15) to probed SERPINA3-chymotrypsin complexes by native MS. We quantified proteoforms of both unliganded apo forms and the complexes (Figures 4B, S16A, and S16B). Interestingly, we found that mono-fucosylation stabilizes the complex while further additions of fucose residues to the mono-fucosylated form, attenuate the interaction (Figures 4C, S16C, and S16D). Since fucose residues on SERPINA3 can be either core- or antennary linked, we hypothesized that core- and antennary fucosylation may have opposite effects on SERPINA3-protease interactions.

To test the hypothesis, we applied the triple exoglycosidase digestion strategy outlined above to transform the fucosylated isomers into two distinct glycans with a mass difference of 366 Da (Figure S16E). Native MS analysis of the treated SERPINA3-chymotrypsin mixture revealed SERPINA3 primarily carries one core fucosylation and up to six antennary fucose residues (Figures 4D and S16F). We then compared the ratios of complex to apo-form SERPINA1 with different antennary fucosylation statuses. We found the ratios are inversely proportional to the number of antennary fucose residues (Figure 4E), implying that antennary fucosylation stoichiometrically impairs SERPINA3-chymotrypsin interactions. We further examined the fucosylation status of Asn271 by glycoproteomics and confirmed the existence of multi-antennary fucosylation at this site (Figure S17). Given that antennary fucosylation attenuates SERPINA3-chymotrypsin binding, we attributed the stabilization effect of mono-fucosylation to core-fucose residues on SERPINA3. Since the N-glycan on Asn271 is proximal to the RCL, we ascribe the stoichiometric regulation of fucosylation to the multiple fucose residues on Asn271.

In summary, these results suggest that antennary fucosylation (Galβ1-4(Fucα1-3/4)GlcNAc trisaccharide) attenuates SERPINA3-chymotrypsin binding while core fucosylation (Fucα1-6) enhances its interactions. As the N-glycan antenna (Galβ1-4GlcNAc disaccharide) is essential for core-fucose stabilization, we interrogated whether N-glycan branching, in terms of Galβ1-4GlcNAc disaccharide, regulates SERPINA3-chymotrypsin interactions (Figure 4F). We further probed the N-glycan branching events on desialylated apo-SERPINA3 and SERPINA3-chymotrypsin complexes (Figures 4G and S18). Comparing the ratios of complex to apo forms of SERPINA3 with different N-glycan branching levels (number of additional Gal-GlcNAc units) (Figure 4H), we find that the ratio of complex to apo SERPINA3 is proportional to the number of additional Gal-GlcNAc residues on SEPINA3. These results imply that N-glycan branching stabilizes SERPINA3-chymotrypsin interactions in a stoichiometric manner (Figure 4H).

### Implications for oligosaccharide mediated SERPIN interactions

**Figure 5:**
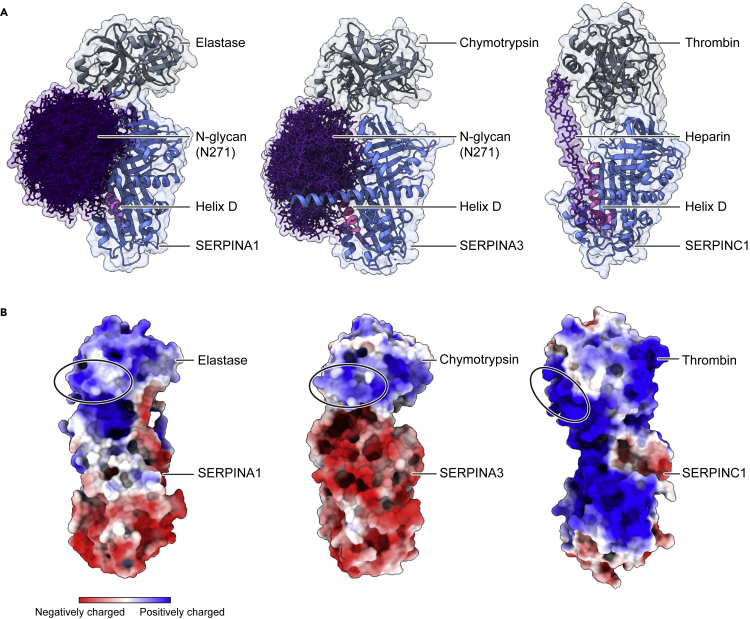
Structural analysis of SERPIN-protease complexes (A) Structural comparison of SERPINA1-elastase, SERPINA3-chymotrypsin, and SERPINC1-thrombin-heparin complexes (PDB:1TB6). N-glycan on Asn271 is modeled on SERPINA1/A3. The positively charged helix D in SERPINA1/A3 and SERPINC1 are highlighted in pink. (B) The surface electrostatic potential of SERPINA1-elastase, SERPINA3-chymotrypsin, and SERPINC1-thrombin complexes. The loop 3 and 6 of proteases at the interfacial domain are highlighted in black circles.

Since the bulky N-glycan on Asn271 of SERPINA1/A3 regulates interactions via contacts with the RCL, we simulated conformations of the sialylated bi-antennary N-glycan on Asn271 in the SERPINA1/A3-protease complexes (Figures 5 and S19). We noted that the glycan on Asn271 shields helix D and the serine protease. Helix D is a well-defined oligosaccharide binding domain in several SERPINs, including SERPINC1 (Antithrombin), SERPIND1 (Heparin cofactor 2), and SERPINE2 (Glia-derived nexin) (Figure S20).^32^ Therefore, we examined the crystal structure of the SERPINC1-thrombin-heparin complex to inform the oligosaccharide regulation of the SERPINA1/A3-protease interactions (Figure 5).^33^ SERPINC1 is an endogenous inhibitor of thrombin, involved in the coagulation cascade, its positively charged helix D is available for oligosaccharide binding, since the corresponding Asn271 glycan is absent. Negatively charged heparin is known to bind tightly to helix D to regulate SERPINC1-thrombin interactions via two mechanisms.^33^ The binding of heparin to SERPINC1 rearranges the RCL conformation and exposes the reactive sites. Alternatively, heparin bridges the protease and inhibitor to stabilize the non-covalently linked complex before enzymatic reaction. Therefore, it follows that the Asn271 glycan on SERPINA1/A3 may regulate the protease inhibitor interactions via similar mechanism as heparin to SERPINC1.

## Discussion

**Figure 6:**
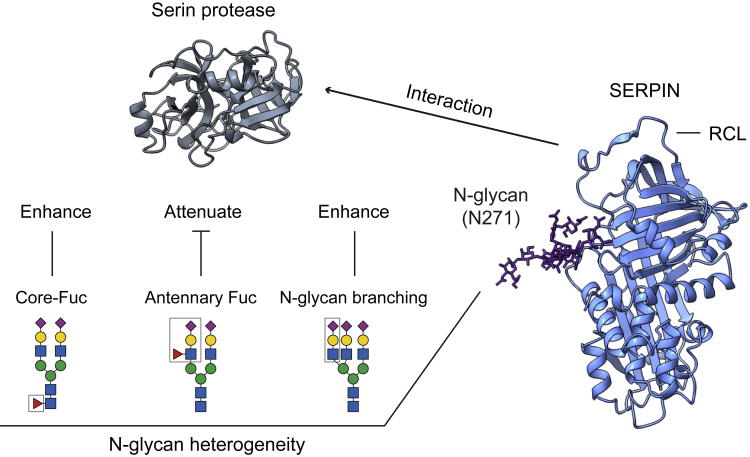
A general model for N-glycan regulation of SERPIN-serine protease interactions The N-glycan on Asn271 plays a regulatory role for SERPIN-protease interactions. Core fucosylation and N-glycan branching enhance SERPIN-protease complex formation while antennary fucosylation attenuates the interactions.

Depiction of proteoform-specific interactions is crucial to understanding how diverse proteoforms regulate protein functions. Here, we developed a native MS protocol to monitor individual SNPs on SERPIN-protease complexes and to relate this to site-specific glycan heterogeneity in SERPIN-protease interactions. We introduced sequential exoglycosidase treatments to differentiate core and antennary fucosylated structural isomers in glycoprotein complexes and performed MD simulation to distinguish site-specific glycan shielding effects on the reactive domain. We revealed that the N-glycan on Asn271 modulates SERPINA1/A3-protease interactions, and the position of the fucose residue (core and antennary) and N-glycan branching determines the regulatory role for SERPINA1/A3 interactions (Figure 6). Importantly, this glycosylation site (Asn271) is conserved in seven members of the SERPIN clade A, including six inhibitory proteins, namely SERPINA1, A2, A3, A5, A10, and A12, and one non-inhibitory protein, SERPINA6 (Figure S21A). For SERPINA2, A5, A10, and A12, the N-glycan on this site is also close to the RCL (Figure S21B). Therefore, we propose that this N-glycosylation may play a regulatory role for all four inhibitory SERPINs. Indeed, previous reports described the corresponding N-glycosylation sites, namely Asn243 in SERPINA5 and Asn267 in SERPINA12, as important for protease inhibitory activity.^34^^,^^35^ The non-inhibitory member, SERPINA6 is a corticosteroid-binding globulin that transports glucocorticoids and progestins in human blood.^13^ Notably, the corresponding glycosylation site Asn260 is proximal to the corticosteroid-binding site and regulates corticosteroid-binding, while the conserved Glu389 in SERPINA6 (Glu400 in SERPINA1) is essential for corticosteroid-binding (Figure S21C).^36^

In this study, exoglycosidase treatment is key to reducing the complexity of native mass spectra for proteoform assignment and to distinguishing fucosylated isomers. We removed all Neu5Ac residues on SERPINA3 to avoid ambiguous assignment of two fucose residues (292.29 Da) and one Neu5Ac residue (291.26 Da). To explore the role of Neu5Ac residues, we modeled fully glycosylated apo-SERPNA3 and SERPINA3-chymotrypsin complexes with and without Neu5Ac residues (Figure S22). We found that some N-glycan conformers on Asn271 in SERPINA3 are proximal to chymotrypsin. Therefore, sialylation may affect fucosylation regulation of SERPINA3-serine protease interactions. Interestingly, several serine proteases, namely TMPRSS2, cathepsin G and chymase have also been reported to be modified with N-glycans. Therefore, we modeled fully glycosylated SERPINA1-TMPRSS2, SERPINA3-cathepsin G, and SERPINA3-chymase complexes to investigate how glycans on these serine proteases may impact SERPIN-protease interactions (Figure S23). We found that the glycans on TMPRSS2 and cathepsin G are distal to SERPINs. Interestingly, the N-glycan on Asn103 in chymase clashes with Asn106 N-glycan in SERPINA3 (Figure S23C). This steric constraint between N-glycans may be also involved in SERPINA3-chymase interactions.

Of recent interest, genomic and glycoproteomic studies of recovered COVID-19 patients revealed that fucosyltransferases and plasma glycoprotein fucosylation levels are associated with critical illness from COVID-19.^37^^,^^38^^,^^39^ Based on our results, we speculate that additional fucose residues on SERPINA1 may regulate SERPINA1-TMPRSS2 interactions. As a consequence, these additional residues may therefore affect TMPRSS2-mediated SARS-CoV-2 spike protein priming and viral infection.^40^ Interestingly, we found that regulation of the SERPINA1-serine protease interaction by fucosylation could be affected by a single amino acid variant (E400D) in SERPINA1, which is proximal to the N-glycan on Asn271 (Figure S6B). This finding implies that the variants, namely E400 or D400, may influence the severity of COVID-19. To our knowledge, there is no report of proteoform profiling of SERIPNA1 variants in COVID-19. We performed data analysis of a well-established proteomics dataset from COVID-19 patients to examine the expression levels of the E400 and D400 variants of SERPINA1.^41^ We found significantly higher expression levels of SERPINA1^D400/D400^, but not SERPINA1^E400/E400^ in patients with severe symptoms (Figure S24). While it is tempting to speculate that the D400 mutation would affect hydrogen-bonding interactions with the N-glycan on Asn271, thereby impacting the RCL and its interactions with TMPRSS2, further details about the synergic effects of glycosylation and SNPs on SERPINA1 functions will be required to make definite conclusions.

In this study, we probed the circulating plasma SERPINs that are primarily secreted by hepatocytes. Interestingly, alveolar macrophages and monocytes that are responsible for local SERPIN-protease balance in lung also secret SERPINs.^42^ Probing the proteoforms of SERPINs from alveolar immune cells will inform how inflammatory stress reshapes the SERPIN proteoforms in lung diseases and affects local SERPIN-protease balance. Augmentation therapy using supplementary SERPINA1 to elevate circulating levels in patients is the only approved therapy for AATD-related lung diseases, including chronic obstructive pulmonary diseases and emphysema.^43^ Moreover, recent clinical studies suggested that SERPINA1 is also a promising anti-inflammatory therapeutic to treat obesity-associated insulin resistance, cystic fibrosis, and COVID-19.^44^^,^^45^ To date, all FDA-approved SERPINA1 products are directly purified from the pooled plasma of healthy donors. Previous studies of therapeutic recombinant SERPINA1 focused on protein engineering of the protein backbone to stabilize the protein structure and improve the circulation half-life.^46^ Our results elucidate the regulation of N-glycan on Asn271 to SERPINA1-protease interactions and highlight an opportunity to design glycoengineered recombinant SERPINA1 to enhance protease inhibition activity.

Structural biology tools, namely cryoelectron microscopy and X-ray crystallography provide the structural basis to link disease-related mutations to protein structure and interactions. However, these structural biology approaches are limited in elucidating proteoforms of endogenous protein complexes with compositional and structural heterogeneities, especially the flexible glycosylation. Therefore, a combination of structural and MS-based approaches is essential to map proteoform information to structural models.^47^^,^^48^^,^^49^ Importantly, the native MS approach provides a unique quantitative readout of the repertoire of proteoforms on protein complexes and connects specific proteoforms to protein interactions. Here, we elucidated site-specific proteoform regulation of SERPIN-protease complexes by combining native MS platform and computational simulations. Our approach has widespread applicability providing opportunities to characterize not only plasma proteins but also endogenous membrane receptor/transporter complexes to thereby inform the interactions and glycosylation status of disease-related proteoforms.

## Experimental procedures

### Resource availability

#### Lead contact

Further information and requests for resources and reagents should be directed to and will be fulfilled by the lead contact, Professor Carol V. Robinson (carol.robinson@chem.ox.ac.uk).

#### Material availability

This study did not generate new unique reagents.

### Materials

Human plasma was from a healthy female donor (Cambridge Biosciences). Human SERPINA1 affinity-purified from pooled plasma was purchased from Sigma-Aldrich (Steinheim, Germany). Human SERPINA3, human elastase, formic acid, ammonium acetate (7.5 M solution), and ammonium bicarbonate were purchased from Sigma-Aldrich (Steinheim, Germany). Liquid chromatography (LC) grade water, acetonitrile, and Tris were purchased from Merck. MS grade trypsin and chymotrypsin were purchased from Promega (Madison, WI, USA). Neuraminidase (α2-3,6,8,9), galactosidase (β1-4), and β-*N*-acetylglucosaminidase were from New England Biolabs.

### Sample preparation

SERPINs were buffer-exchanged to 100 mM sodium citrate (pH 5.5), and incubated with glycosidase (glycoprotein:glycosidase = 50 μg:1 unit) at 37°C overnight for glycosidase digestion.^27^ All SERPINs and proteases were extensively desalted with 1 M ammonium acetate using Amicon centrifugal filters (MWCO 10 K, Millipore) and then diluted into 200 mM ammonium acetate for native MS analysis.

### Native MS analysis

SERPINs and proteases were stored on ice, mixed, and loaded into gold-coated needles prepared in-house and analyzed by native MS on a modified Orbitrap Eclipse Tribrid mass spectrometer in intact protein detection mode (Thermo Fisher Scientific).^22^ Briefly, ion transmission was tuned for high *m*/*z* ions, higher-energy collisional dissociation (HCD) collision energies were optimized in the ion routing multipole for higher charge state species and the ion trap was optimized to trap, isolate and activate high *m*/*z* ions (up to 8,000 *m*/*z*). Typical MS settings were spray voltage 1.2 kV, and source temperature 150°C. The source fragmentation voltage was kept at 0–50 V to minimize in-source dissociation of glycoprotein complexes.^50^

### Data processing

Native MS raw data processing and visualization were performed with Xcalibur 4.1 (Thermo Fisher Scientific). Protein and glycan mass calculations were based on amino acid and monosaccharide average residue masses. Glycoproteomics data were processed with Xcalibur 4.1 and PGlyco (version 2.0).^51^ Glycoprotein structures were retrieved from PDB database and modeled with the Solution Builder module of CHARM-GUI^52^ and GlycoSHIELD (version 0.1).^53^ MDs simulation was performed with GROMACS 2020 with CUDA feature.^54^ The glycoprotein molecular graphics and analysis were performed with UCSF ChimeraX (version 1.2.5)^55^ and VMD (version 1.9.3).^56^

## Data Availability

The raw MS data have been deposited at Figshare under the (https://doi.org/10.6084/m9.figshare.21206150) and are publicly available as of the date of publication. Detailed experimental procedures can be found in the supplemental information.

## Supplemental information

Document S1. Supplemental experimental procedures, Figures S1–S24, and supplemental references

Document S2. Article plus supplemental information
