# Supplementary material for: Connecting single-nucleotide polymorphisms, glycosylation status, and interactions of plasma serine protease inhibitors
Source: Chem. 2023 Mar 9;9(3):665–81. doi: 10.1016/j.chempr.2022.11.018 (PMC10914678; doi:10.1016/j.chempr.2022.11.018)
Supplement: Document S1. Supplemental experimental procedures, Figures S1–S24, and supplemental references [file mmc1.pdf]

Chem, Volume *9*

Supplemental information

Connecting single-nucleotide polymorphisms, glycosylation status, and interactions of plasma serine protease inhibitors

Di Wu, Manman Guo, and Carol V. Robinson

Table of contents

I. Supplemental experimental procedures ………………………………………………. 1
Detailed Methods ………………………………………………………………. 1
Figure S1 ………………………………………………………………………. 5
Figure S2 ………………………………………………………………………. 6
Figure S3 ………………………………………………………………………. 7
Figure S4 ………………………………………………………………………. 8
Figure S5 ………………………………………………………………………. 9
Figure S6 ………………………………………………………………………. 10
Figure S7 ………………………………………………………………………. 11
Figure S8 ………………………………………………………………………. 12
Figure S9 ………………………………………………………………………. 13
Figure S10 ……………………………………………………………………… 14
Figure S11 ……………………………………………………………………… 15
Figure S12 ……………………………………………………………………… 16
Figure S13 ……………………………………………………………………… 17
Figure S14 ……………………………………………………………………… 18
Figure S15 ……………………………………………………………………… 19
Figure S16 ……………………………………………………………………… 20
Figure S17 ……………………………………………………………………… 21
Figure S18 ……………………………………………………………………… 22
Figure S19 ……………………………………………………………………… 23
Figure S20 ……………………………………………………………………… 23
Figure S21 ……………………………………………………………………… 24
Figure S22 ……………………………………………………………………… 25
Figure S23 ……………………………………………………………………… 26
Figure S24 ……………………………………………………………………… 27

II. Supplemental references …………………………………………………………. 28

Supplemental experimental procedures

Detailed Methods

Sample preparation of human plasma. Fresh human plasma (reference number: RDN-6711552, Research Donors Ltd.) was separated from fresh whole blood from a female donor collected in a vacuette tube containing sodium citrate anticoagulant. This human plasma was then centrifuged at 12000g for 10 min and the supernatant diluted with 1 M ammonium acetate to a final protein concentration of 1 mg/ml and buffer-exchanged into 200 mM ammonium acetate (pH 7.0) using a 10 kD MWCO centrifugal filter (Amicon Ultra-0.5 ml, Millipore) prior to native MS analysis.

Preparation for native MS. The isolated SERPINA1, SERPINA3, elastase and chymotrypsin were reconstituted in 50 mM Tris, pH7.4 at a concentration of 1 mg/ml for further treatment. Glycoproteins were buffer-exchanged into 200 mM ammonium acetate (pH 7.0) using a 10 kD MWCO centrifugal filter (Amicon Ultra-0.5 ml, Millipore) prior to native MS analysis.

Exoglycosidase treatment. The exoglycosidase digestion protocol was adapted from Chen et al [1]. Briefly, 200 µg glycoprotein (1 mg/ml) was buffer-exchanged into 50 mM sodium citrate buffer (pH 5.5) using a 10 kD MWCO centrifugal filter (Amicon Ultra-0.5 ml, Millipore). The glycoproteins were incubated first desialylated with neuraminidase (α2-3,6,8,9) then further incubated with galactosidase (β1-4) to remove the Gal residues and then incubated β-*N*-acetylglucosaminidase to release the GlcNAc residues (all three reactions performed at 37 ºC overnight). The exoglycosidase-treated glycoproteins were buffer-exchanged to 200 mM ammonium acetate for MS analysis.

Resolving isotopic peaks of human plasma proteins using native MS. Plasma proteins and SERINA1 were analysed on a modified Orbitrap Eclipse platform. The human plasma proteins and SERPINA1 were extensively desalted before the analysis. For native MS analysis, the mass spectra were firstly acquired using a resolution of 17500 to ensure complete removal of sodium and potassium ions, and then acquired using a resolution of 500000 with the spectra averaging function for resolving isotopic peaks.

Native top-down MS analysis of SERPINA1. The desalted SERPINA1 was analysed on a modified Orbitrap Eclipse platform. The major proteoform of SERPINA1 with three sialylated bi-antennary N-glycans was selected (m/z 3928 with a window of m/z 20, charge state +13) and further fragmented with HCD (20 V). The MS/MS spectra were manually analysed using Xcalibur (version 4.1, ThermoFisher).

Proteomics analysis of glycoproteins and plasma samples. Glycoproteins were buffer-exchanged to 100 mM Tris buffer (pH 8.0) containing 8 M urea and 5 mM dithiothreitol (DTT) then incubated at 56 ºC for 20 min and buffer-exchanged to 100 mM Tris buffer (pH 8.0) with 20 mM iodoacetamide (IAA).

The samples were then alkylated at room temperature for 20 min in the dark and buffer-exchanged to 50 mM NH_4HCO_3 (pH 8.0). The glycoproteins were then transferred to a new Eppendorf tube and digested with trypsin at 37 ºC overnight for proteolytic digestion. The digested peptides were dried and reconstituted with 1% formic acid for LC-MS/MS analysis. The tryptic peptides (100 ng) were analyzed on a Dionex Ultimate 3000 UHPLC coupled to an Orbitrap Eclipse Tribrid mass spectrometer (Thermo Fisher Scientific). The peptides were firstly loaded onto a 75 μm×2 cm pre-column and separated on a 75 μm×15 cm Pepmap C18 analytical column (Thermo Fisher Scientific) with a binary buffer system. Buffer A was 0.1% formic acid (FA) in 100% H_2O and buffer B was 0.1% FA in 80% acetonitrile with 20% H_2O. The Eclipse mass spectrometer was operated in data-dependant acquisition mode with one full MS scan followed by MS/MS scans with higher-energy collision-induced dissociation (HCD) fragmentation.

Proteomics data analysis. The RAW data were processed with Maxquant (version 1.6.2) [2] for protein identification and PGlyco (version 2.0) [3] for glycopeptide identification. Quantification of the site-specific microheterogeneity was performed manually using Xcalibur (version 4.1). The extracted ion chromatogram (XIC) of each glycopeptide was processed with 50 ppm mass tolerance and a 7-point Gaussian smoothing. The area under the curve (AUC) was integrated for glycopeptide quantification.

Protein structure modelling. Protein structures of SERPINA1 (PDB: 3NE4), SERPINA3 (PDB: 6HGE), trypsin (PDB: 1QPA and 1OPH), elastase (PDB: 1BMA), chymotrypsin (PDB: 4CHA), Thrombin-SERPINC1-heparin complex (PDB: 1TB6), SERPIND1 (PDB: 1JMJ), SERPINE2 (PDB: 4DY0), SERPINA6 (PDB: 2V95), cathepsin G (PDB: 1KYN), chymase (PDB:1KLT) were retrieved from the PDB. SERPINA1, 2, 3, 5, 10, 12 in Figure S21 and TMPRSS2 in Figure S23 were retrieved from the AlphaFold protein structural database (alphafold.ebi.ac.uk). The protein structures were processed using University of California, San Francisco Chimera program (version 1.15 and version X 1.2.5) [4].

Sequence alignment. The protein sequences were retrieved from UNIPROT database. The protein sequences were introduced to Jalview program (version 2.11.2.2) [5] and aligned using T-coffee algorithm with default settings [6]. The aligned sequences were highlighted using Clustalx colour scheme.

Molecular dynamics simulation. The crystal structure of human SERPINA1 (PDB: 3NE4) was used as a template for the M1V variant. The M3 (E400D) variant structure was generated using PyMOL and the glycoprotein structures for MD simulation were generated using CHARM-GUI (http://www.charm-gui.org) [7,8]. The protein N-terminus and C-terminus were patched with acetylation and methylamidation, respectively. The bi-antennary N-glycans with α2,6-linked *N*-acetylneuraminic acid residues (the most abundant N-glycan on SERPINA1) were modelled on Asn46, Asn83 and Asn247 using Glycan Reader and Modeler [9]. The glycoproteins were then placed in a periodic box of TIP3P water molecules with 150 mM KCl. The box boundaries are 1.5 nm away from the glycoprotein. The

CHARMM36m force field was used for the polypeptide chain and carbohydrate residues. All simulations were performed at 303.15 K. After 5000 steps of energy minimization, all atoms were equilibrated for 200 ps under constant particle number, volume and temperature (NVT) conditions. The simulations were then performed using under constant particle number, pressure and temperature (NPT) conditions using GROMACS (version 2021) [10]. The temperature was maintained at 303.15 K using a Nose-Hoover thermostat with a time constant of 1 ps. A Parrinello-Raham barostat was employed for pressure regulation. Van der Waals interactions were treated using a forced-based switching function between 10 and 12Å. Long-range electrostatics were treated with the particle-mesh Ewald (PME) method. SHAKE was used to constrain all bonds involving hydrogen atoms. The data analysis (RMSD, RMSF and H-bonding) was performed using built-in functions GROMACS and VMD [11].

Glycan modelling on SERPINA3. A sialylated bi-antennary N-glycan was modelled onto Asn271 in SERPINA3 using GlycoSHIELD (version 0.1) following the protocol described in Park, S. J. *et al.* [12].

Glycan modelling on SERPINA1-TMPRSS2, SERPINA3-Chymas and SERPINA3-Cathepsin G complexes. Sialylated bi-antennary N-glycans were modelled on Asn70, Asn107 and Asn271 in SERPINA1 and Asn83, Asn106, Asn127, Asn186 and Asn271 in SERPINA3. Two sialylated bi-antennary N-glycans were modelled on Asn213 and Asn249 in TMPRSS2 extracellular domain. An M2 tetrasaccharide Manα1-6Manβ1-4GlcNAcβ1-4GlcNAc was modelled on Asn71 in cathepsin G. Two sialylated bi-antennary N-glycans were modelled on Asn80 and Asn103 in chymase. The protein structure files in PDB format were prepared using Chimera and CHARM-GUI. All glycan modelling was performed using GlycoSHIELD. The visualization of the glycoprotein complexes was performed using Chimera.

Database search for the SERPINA1 variants with E400D mutation (rs1303) in COVID-19 proteomics dataset. We analysed two single amino acid variants (SAVs) of SERPINA1, namely E400 (canonical) and D400 (rs1303) in a well-established plasma dataset from COVID-19 patients [13]. In brief, plasma samples from 13 patients diagnosed as severe symptoms, 16 patients diagnosed as mild symptoms, and 13 healthy donors at Wuhan Jinyintan Hospital were used in this study. The plasma samples were digested with trypsin protease, labelled with 11-plex tandem mass tag (TMT), fractionated with strong cation exchange chromatography (SCX) and processed with liquid chromatography with tandem mass spectrometry (LC-MS/MS). To probe the SERPINA1 protein expression level in each sample, we first searched the quantitative MS dataset using Maxquant (version 2.0.3) [2] with an in-house constructed human proteome database (UNIPROT:UP000005640) with SARS-CoV-2 proteome [13]. We identified 860 human proteins in this dataset. We then retrieved the SAV information of these 860 identified human proteins from the Single Nucleotide Polymorphism database (dbSNP) and constructed a tailored FASTA file containing the sequences of these 860 human proteins with SAV information. We performed a second-round database search using Maxquant with the tailored FASTA file. The "variation mode" was enabled in Maxquant to search the SAV peptides. We manually

checked the identification of SAV peptides of SERPINA1 (E400 and D400) and extracted the TMT ratios for the following analysis.

Haplotyping SERPINA1 variants in the COVID-19 dataset. We determined the SERPINA1 haplotypes of each donor in COVID-19 dataset based on the TMT ratios of SAV peptides. We focused on two tryptic peptides containing the mutation site (E400D), namely FNKPFVFLMIEQNTK (E400) and FNKPFVFLMIDQNTK (D400). Both Glu (E) and Asp (D) carry a carboxy group in the side chain. The mutation of E to D does not introduce a significant change to the MS responses of these two peptides. Therefore, the ratio of FNKPFVFLMIEQNTK (E400) and FNKPFVFLMIDQNTK (D400) represents the relative abundances of M1V and M3 variants in each sample. The ratio of E400 to D400 can be calculated as:

$$\mathrm{r} = \log_2\left(\frac{\text{TMT ratio}_{\text{FNKPFVFLMIEQNTK}}}{\text{TMT ratio}_{\text{FNKPFVFLMIDQNTK}}}\right)$$

We haplotyped SERPINA1 in each individual based on the ratio of E400 and D400 (r). We used an empirical value of 0.585 ($log_2 1.5$) as the threshold:

	SERPINA1 haplotype
r < -0.585	D400/D400
-0.585< r < 0.585	E400/D400
r > 0.585	E400/E400

Using this approach, we determined the 14 individuals with SERPINA1$^{E400/E400}$, 17 individuals with SERPINA1$^{D400/D400}$ and 3 heterozygous individuals with SERPINA1$^{E400/D400}$. We further extracted the SERPINA1 abundance information from homozygous individuals (SERPINA1$^{E400/E400}$ and SERPINA1$^{D400/D400}$) to investigate E400 and D400 expression levels in COVID-19 patients.

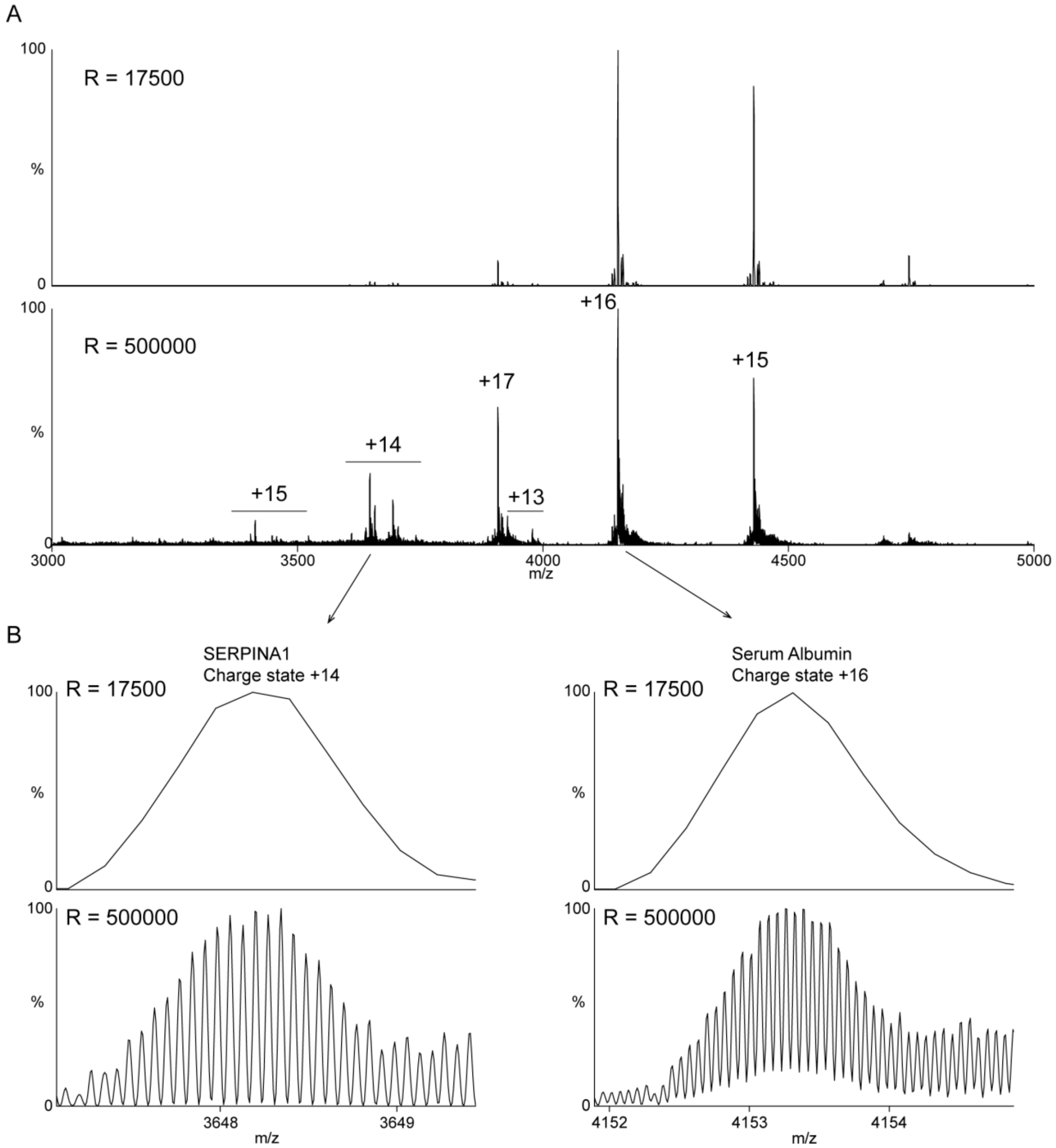

Figure S1. High-resolution native MS analysis of human plasma proteins. A) High-resolution native mass spectra of non-depleted human plasma sample from an individual donor with resolving power (R) of 17500 and 500000. B) High-resolution native MS (resolving power of 500000) recorded isotopic peaks of SERPINA1 and serum albumin.

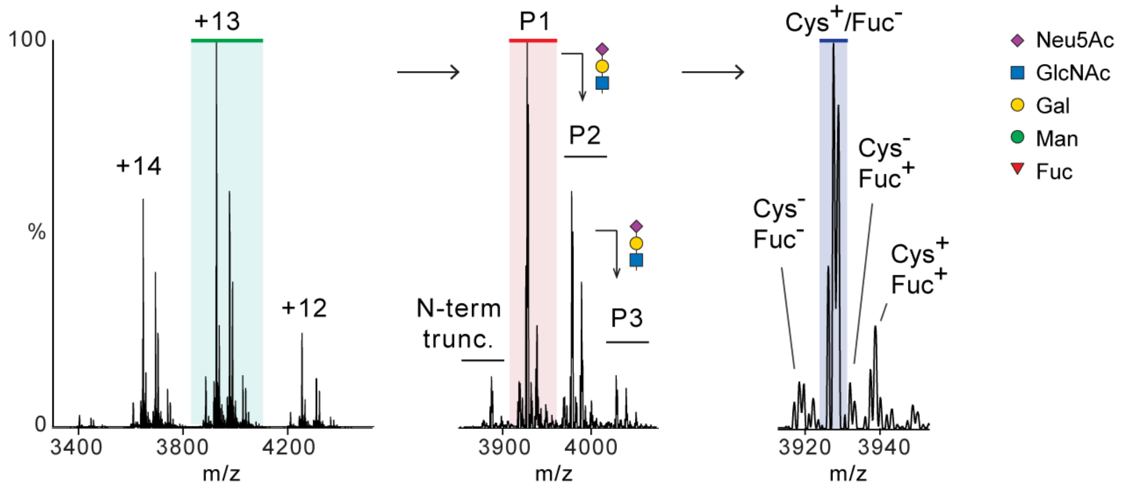

Figure S2. Native MS analysis of SERPINA1. Native MS revealed peaks consistent with N-glycan branching (addition of GlcNAc-Gal-Neu5Ac units, P1 to P3 series), N-terminal truncation, fucosylation (Fuc) and cysteinylation (Cys) status.

A

Variant	Theoretical Mass
M2	51028.10
M1A	51033.12
M3	51047.15
M1V	51061.17

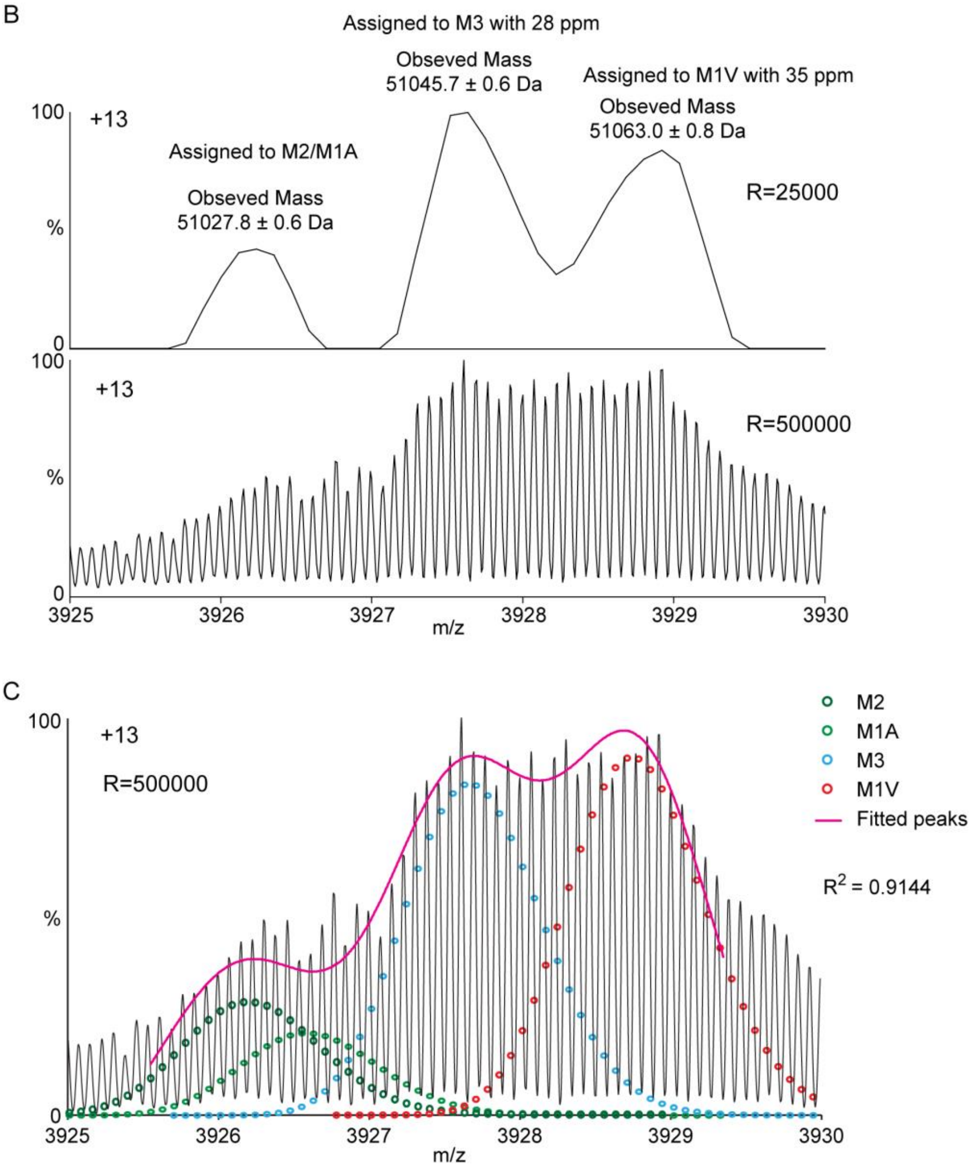

Figure S3. High-resolution native MS analysis of SERPINA1. A) Average masses of SERPINA1 main proteoforms. B) High-resolution mass spectra of SERPINA1 with resolving power of 25000 and 500000. Two major peaks (51045.7 Da and 51063.0 Da) were assigned to M3 and M1V variants, respectively. The smallest peak (51027.8 Da) was assigned to the overlapped M2 and M1A variants. C) theoretical isotopic peaks of M1V, M1A, M2 and M3 were fitted to the high-resolution native mass spectrum of SERPINA1. The coefficient of determination (R^2) of the fitting is 0.9144, suggesting a good correlation between the recorded and fitted peaks.

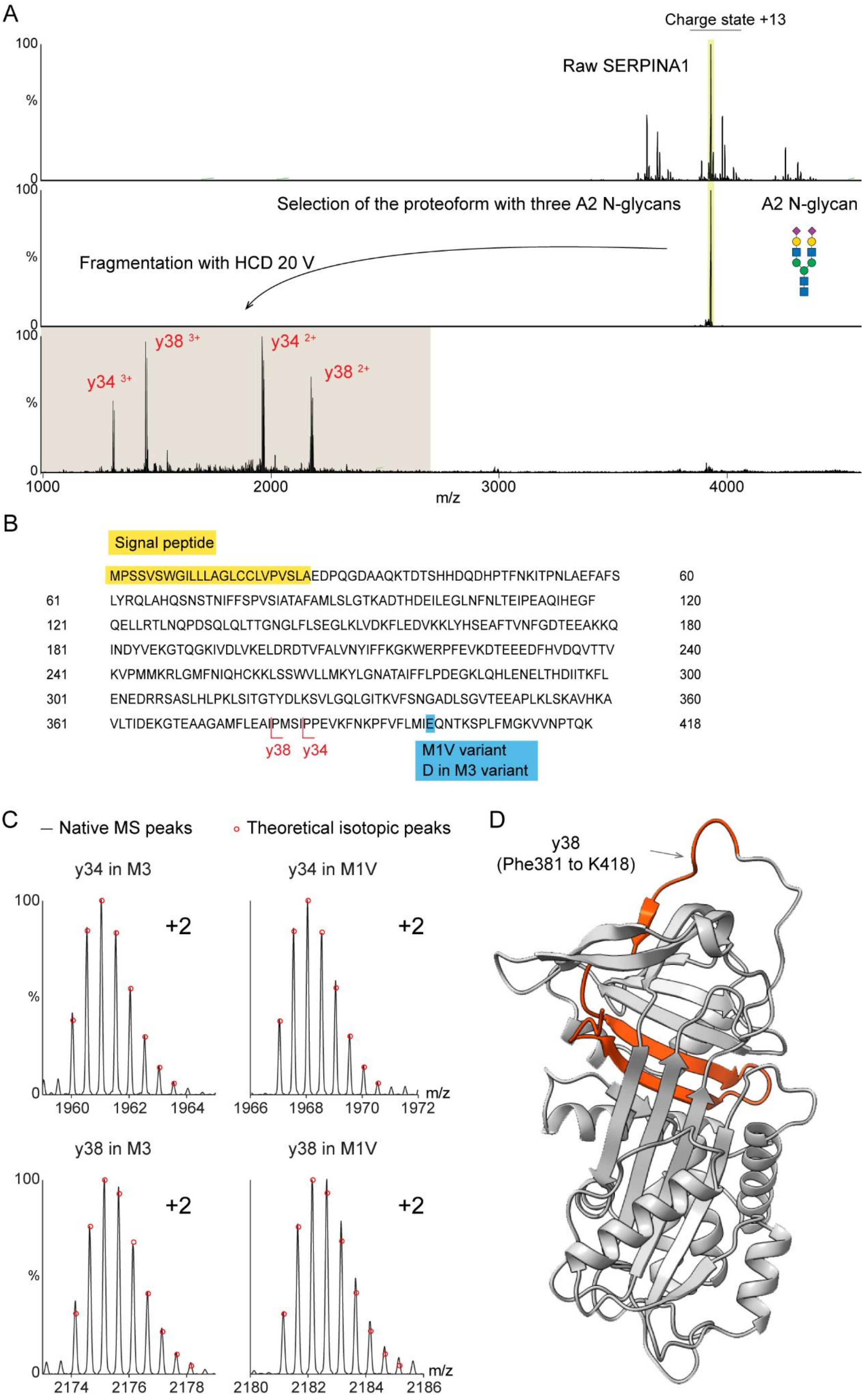
A
Charge state +13
Raw SERPINA1
Selection of the proteoform with three A2 N-glycans
A2 N-glycan
Fragmentation with HCD 20 V
y34 3+
y38 3+
y34 2+
y38 2+
100
%
0
1000
2000
3000
4000
m/z
B
Signal peptide
MPSSVSWGILLLAGLCCLVPVSLAEDPQGDAAQKTDTSHHDQDHPTFNKITPNLAEFAFS 60
61 LYRQLAHQSNSTNIFFSPVSIATAFAMLSLGTKADTHDEILEGLNFNLTEIPEAQIHEGF 120
121 QELLRTLNQPDSQLQLTTGNGLFLSEGLKLVDKFLEDVKKLYHSEAFTVNFGDTEEAKKQ 180
181 INDYVEKGTQGKIVDLVKELDRDTVFALVNYIFFKGKWERPFEVKDTEEEDFHVDQVTTV 240
241 KVPMMKRLGMFNIQHCKKLSSWVLLMKYLGNATAIFFLPDEGKLQHLENELTHDIITKFL 300
301 ENEDRRSASLHLPKLSITGTYDLKSVLGQLGITKVFSNGADLSGVTEEAPLKLSKAVHKA 360
361 VLTIDEKGTEAAGAMFLEAIPMSIPPEVKFNKPFVFLMIEQNTKSPLFMGKVVNPTQK 418
y38 y34
M1V variant
D in M3 variant
C
— Native MS peaks
Theoretical isotopic peaks
y34 in M3
y34 in M1V
y38 in M3
y38 in M1V
+2
100
%
0
1960 1962 1964
1966 1968 1970 1972
2174 2176 2178
2180 2182 2184 2186
m/z
D
y38
(Phe381 to K418)

Figure S4. Native top-down MS analysis of SERPINA1. A) The SERPINA1 proteoform with three fully sialylated bi-antennary N-glycan (A2) was selected and further fragmented with HCD (20 V). The y34 and y38 fragment ions were detected. No glycan fragments were observed. B) The sequence of SERPINA1. Both y34 and y38 fragments cover the C-terminal residues, including the D400/E400 which is the only different amino acid between M1V and M3 variants. C) Mass spectra of SERPINA1 fragments (y34 and y38) from the native top-down analysis. Theoretical ion distributions are indicated by the red dots. D) Structure of SERPINA1. The y38 fragment is highlighted in orange. It covers part of the RCL.

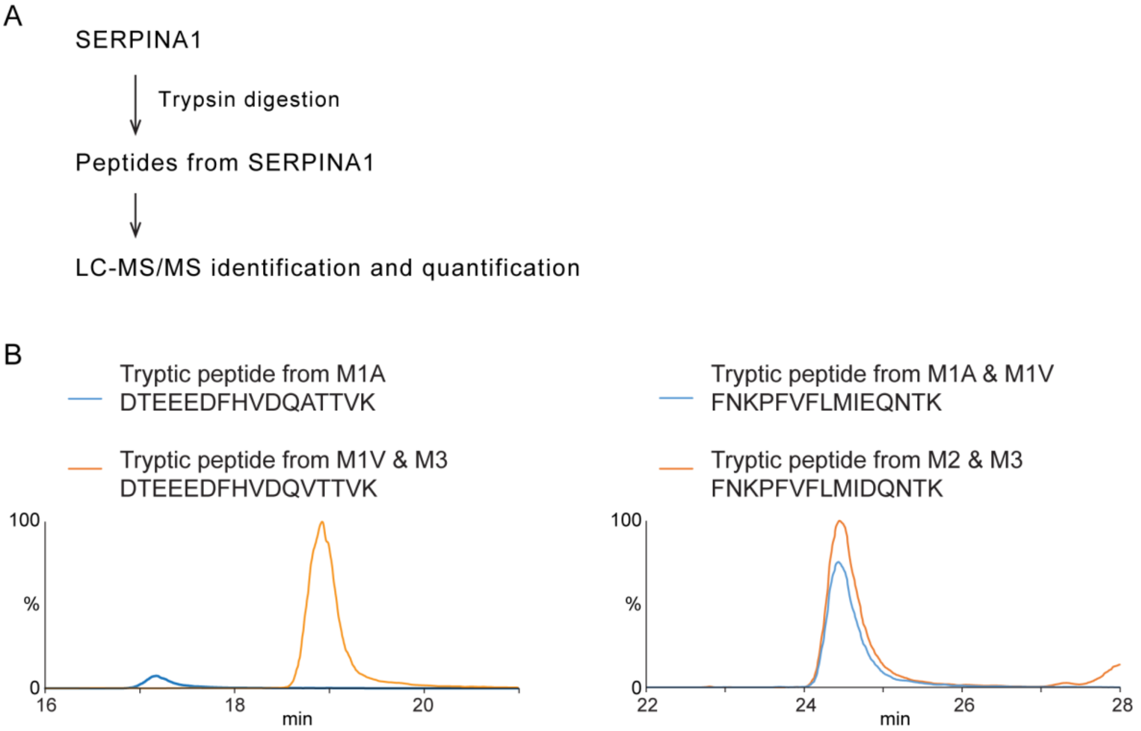

C

Variant	Mutations	dbSNP ID	Amino acid (Codon)	Frequency
M1V	Wild-type	rs6647	V237A (GTG>GCG)	GCG=0.22
M1A	V237A	rs709932	R125H (CGT>CAT)	CAT=0.16
M2	R125H/E400D	rs1303	E400D (GAA>GAC)	GAC=0.25
M3	E400D			

Figure S5. Proteomics analysis of SERPINA1. A) Flow chart of the proteomics analysis of SERPINA1. B) Extracted ion chromatograms (EICs) of the corresponding tryptic peptides to each variant. The tryptic peptide with Val237Ala mutation (DTEEEDFHVDQATTVK) is unique to M1A variant. The other tryptic peptides are not unique to each variant. C) The dbSNP ID and frequency of M1V, M1A, M2 and M3 variants.

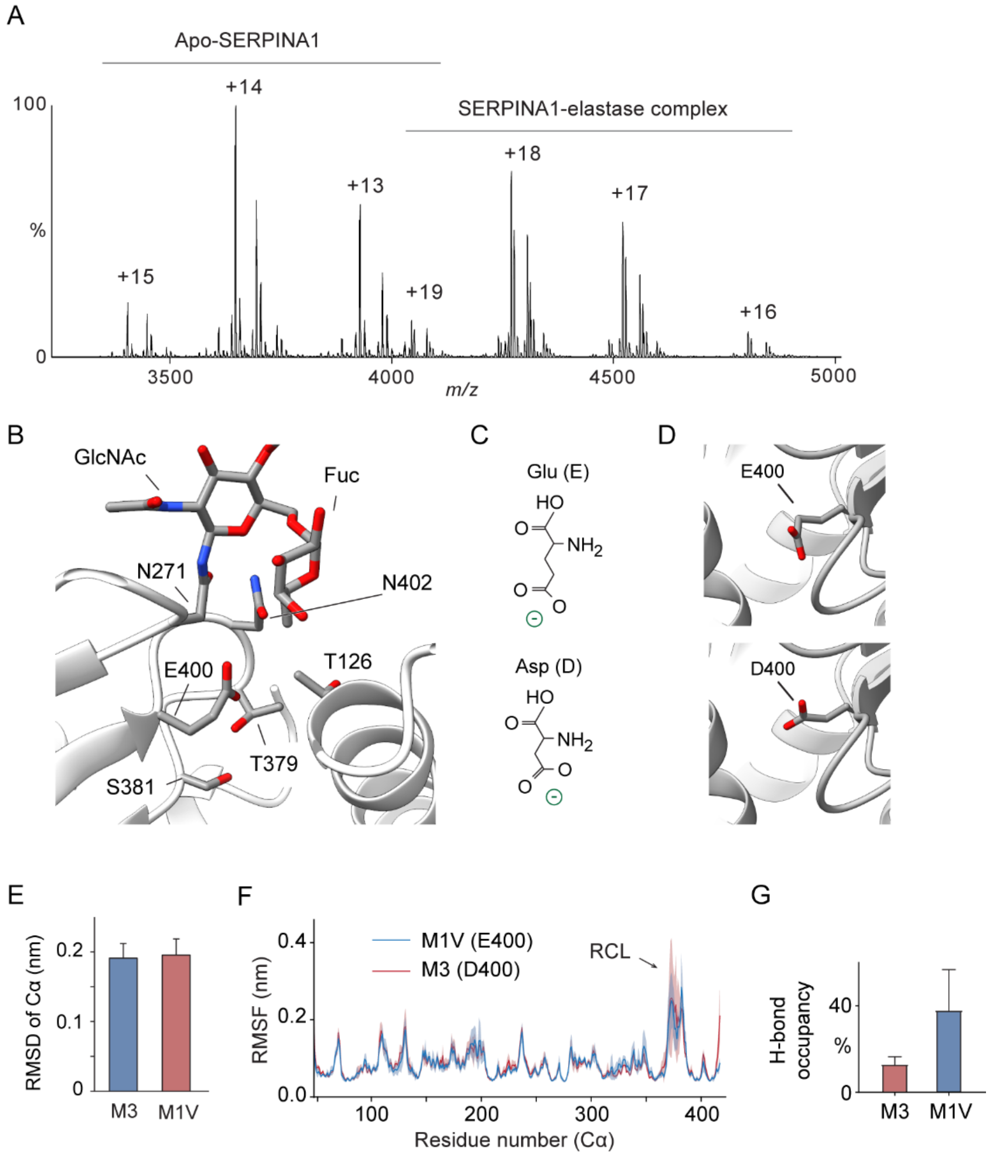

Figure S6. Structural analysis of SERPINA1. A) Native mass spectrum of SERPINA1-elastase complexes. The apo-SERPINA1 and SERPINA1-elastase complex peaks are labelled with the corresponding charge states. B) The side chain of E400 (M1V variant) can form hydrogen bonds with the surrounding amino acid residues (T126, T379, S381, T402). C) Structure of glutamic acid (Glu) and aspartic acid (Asp). D) Structure comparison of Glu400 (M1V variant) and Asp400 (M3 variant). E) The Cα atom root-mean-square deviation (RMSD) plot of M1V and M3 in MD simulations. F) The Cα atom root-mean-square fluctuation (RMSF) plot of M1V and M3 in MD simulations. The 95% confidence intervals for M1V and M3 variants are highlighted in light red and light blue respectively. G) The hydrogen bond (H-bond) occupancies of the side chains of Glu400 (M1V variant) and Asp400 (M3 variant) interacting with Thr126, Thr379, Ser381 and Asn402 in MD simulation.

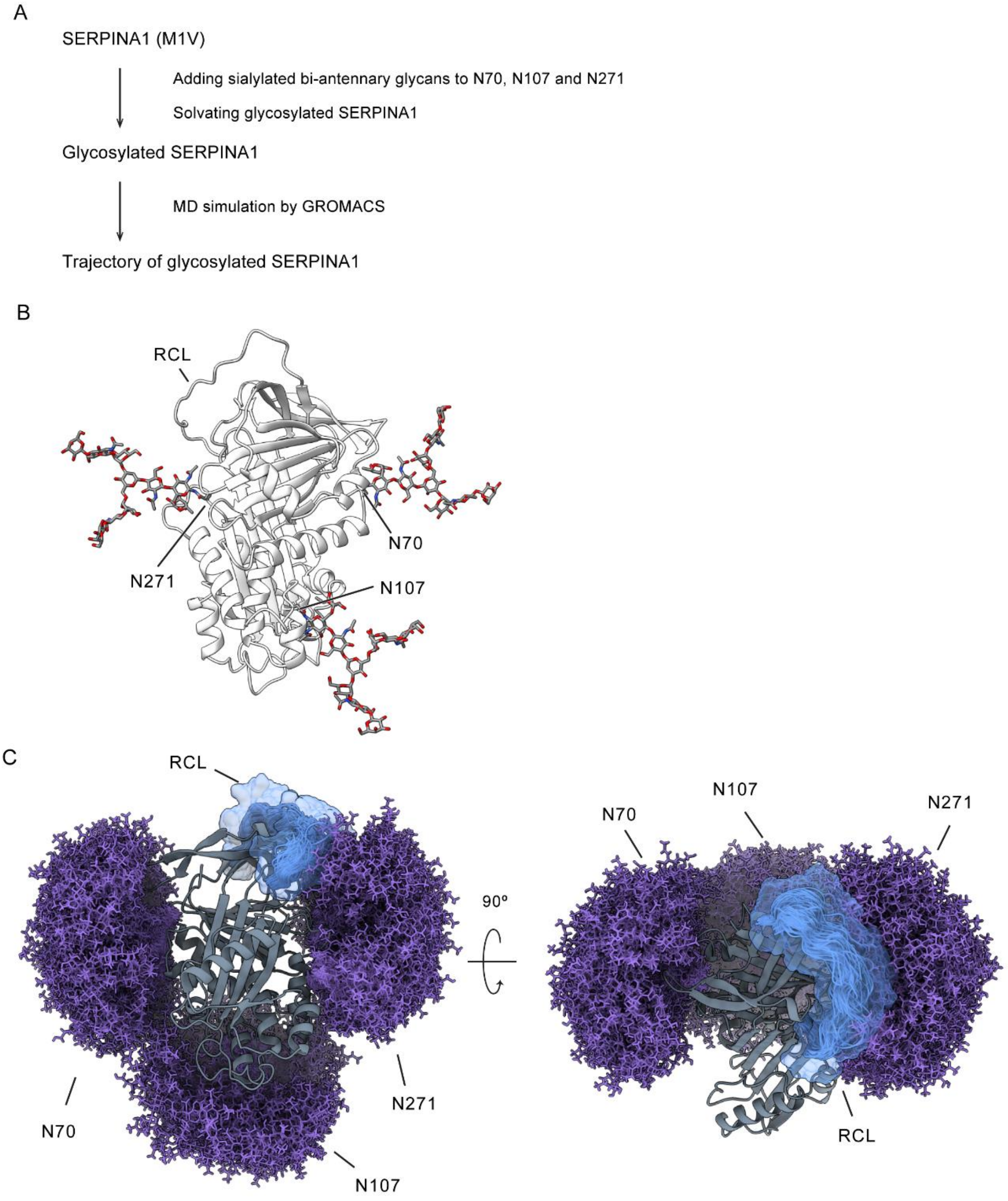

Figure S7. MD simulations of SERPINA1. A) Flow chart of molecular dynamics simulation for glycosylated SERPINA1. B) a modeled SERPINA1 structure with three sialylated bi-antennary N-glycans on N70, N107 and N271. C) Stereo-view of the structure of glycosylated SERPINA1 from the 150 ns MD simulation trajectory simulation. The snapshots of the three N-glycans and RCL conformations (1 frame per ns) are extracted and overlayed.

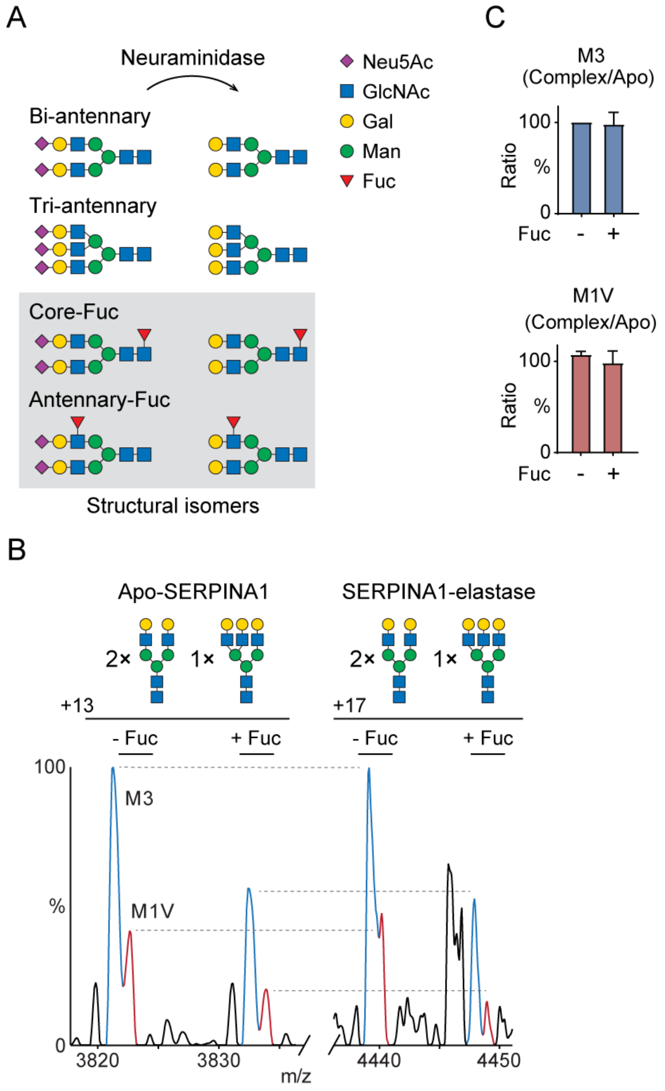

Figure S8. Native MS analysis of desialylated SERPINA1. A) Neuraminidase treatment releases all terminal Neu5Ac residues from N-glycans. However, this treatment cannot distinguish isomeric core- and antennary fucosylated N-glycans. B) Native mass spectra of the desialylated SERPINA1 variants M3 and M1V in apo forms and complex forms with elastase. The N-glycan composition and variants are labeled. C) Bar graphs of the ratio of desialylated SERPINA1-elastase complex to desialylated apo-SERPINA1, with and without fucosylation. Bars show mean ± standard deviation from three individual replicates.

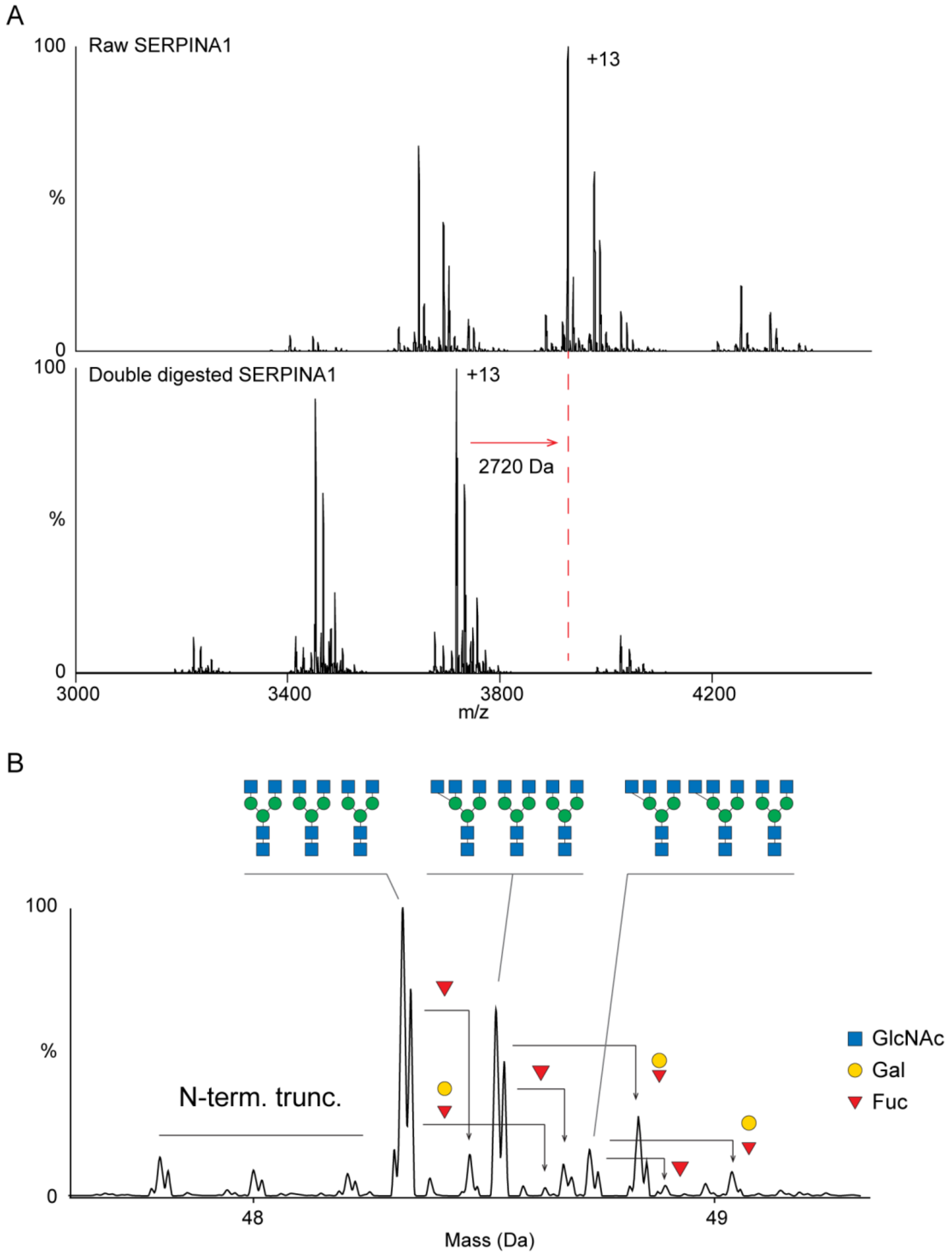

Figure S9. Native MS analysis of double digested SERPINA1. A) Native mass spectra of double exoglycosidase digested SERPINA1 (neuraminidase and galactosidase treated) and raw SERPINA1. All peaks in the double digested SERPINA1 spectrum shift to lower m/z values compared to the untreated form. All Neu5Ac and Gal residues from SERPINA1, are removed during double digestion resulting in a loss of 2720 Da (six Neu5Ac-Gal units) to the main species (base peak). B) Annotation of the zero-charge spectrum of double exoglycosidase digested SERPINA1. We assigned major peaks to the corresponding proteoforms and confirmed complete double digestion.

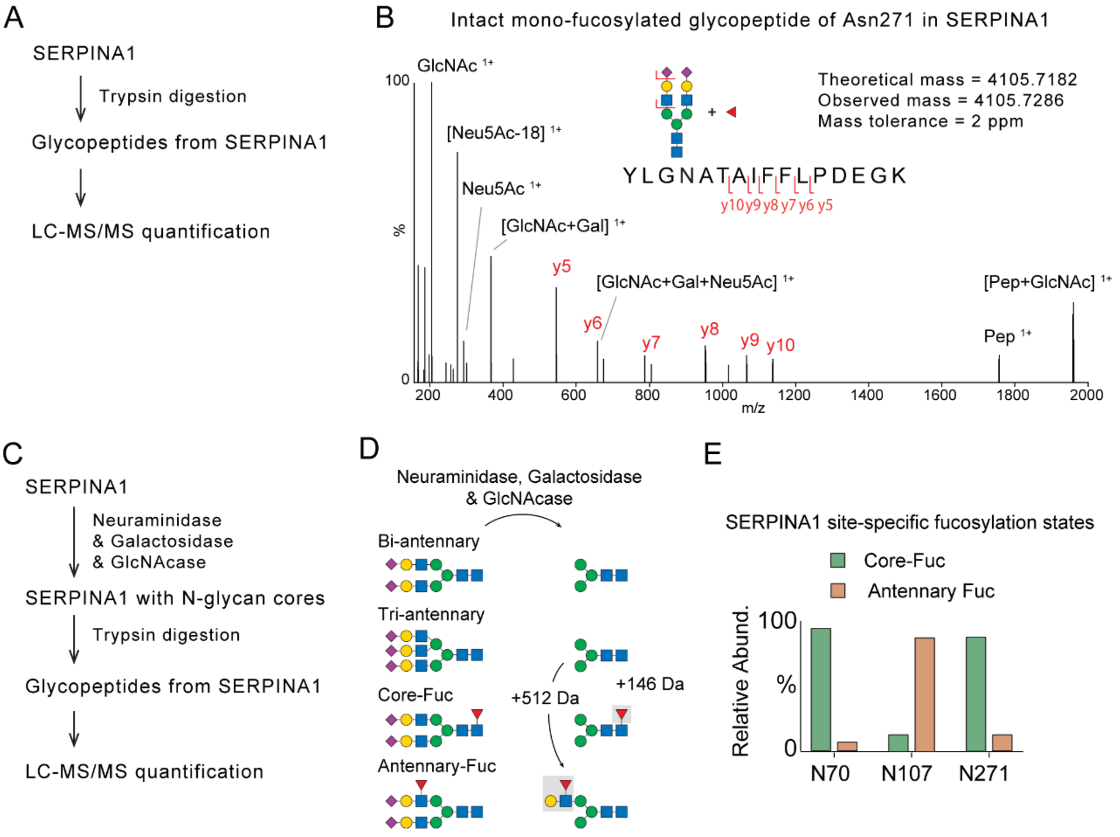

Figure S10. Glycoproteomics analysis of SERPINA1 site-specific fucosylation. Firstly, we performed glycoproteomics of raw SERPINA1 (panel A). We identified the intact mono-fucosylated glycopeptide of Asn271 in SERPINA1 based on the MS/MS spectrum (panel B). We confirmed the presence of fucosylation on Asn271. However, we couldn't distinguish core- or antennary fucosylation based on the MS/MS spectrum. Therefore, we digested SERPINA1 with neuraminidase, galactosidase and GlcNAcase sequentially, and performed glycoproteomics analysis with the triple exoglycosidase digested SERPINA (panel C). The triple exoglycosidase digestion transformed the core and antennary fucosylated N-glycans to different structures (panel D). This enables us to identify and quantify core and antennary fucosylation on each glycosylation site in SERPINA1. Using this strategy, we identified and quantified the core- and antennary fucosylation across all three sites (panel E). To compare the fucosylation levels across all three sites, the total fucosylated (core and antennary fucosylated) tryptic peptide intensities of each site were normalized to 100%. Bar graphs show the relative abundances of core- and antennary fucosylation on Asn70, Asn107 and Asn271. This suggests all three N-glycosylation sites can be either core or antennary fucosylated. Moreover, core-fucosylation dominates Asn70 and Asn271.

A

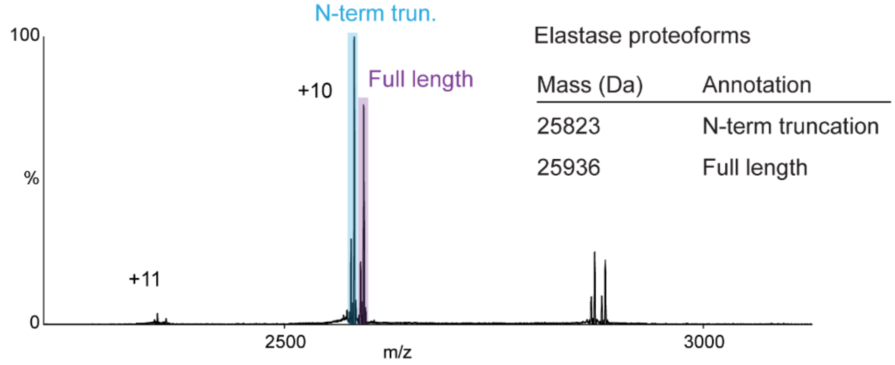

B

SERPINA1 proteoforms

Mass (Da)	Annotation
48327	HexNAc12Hex9
48473	HexNAc12Hex9Fuc1
48635	HexNAc12Hex10Fuc1
48530	HexNAc13Hex9
48676	HexNAc13Hex9Fuc1
48838	HexNAc13Hex10Fuc1
48984	HexNAc13Hex10Fuc2
48733	HexNAc14Hex9
48879	HexNAc14Hex9Fuc1
49041	HexNAc14Hex10Fuc1

C

SERPINA1 with N-term trunc Elastase

Mass (Da)	Annotation
74150	HexNAc12Hex9
74296	HexNAc12Hex9Fuc1
74458	HexNAc12Hex10Fuc1
74353	HexNAc13Hex9
74499	HexNAc13Hex9Fuc1
74661	HexNAc13Hex10Fuc1
74807	HexNAc13Hex10Fuc2
74556	HexNAc14Hex9
74702	HexNAc14Hex9Fuc1
74864	HexNAc14Hex10Fuc1

8 Da

8 Da

SERPINA1 with full length Elastase

Mass (Da)	Annotation
74263	HexNAc12Hex9
74409	HexNAc12Hex9Fuc1
74571	HexNAc12Hex10Fuc1
74466	HexNAc13Hex9
74612	HexNAc13Hex9Fuc1
74774	HexNAc13Hex10Fuc1
74920	HexNAc13Hex10Fuc2
74669	HexNAc14Hex9
74815	HexNAc14Hex9Fuc1
74977	HexNAc14Hex10Fuc1

Figure S11. Native MS analysis of elastase. A) Native mass spectrum of elastase. The N-terminal truncated and full-length forms are highlighted in blue and purple, respectively. B) Theoretical molecular weight and monosaccharide composition of double exoglycosidase digested SERPINA1 glycoforms. C) Theoretical molecular weight of double exoglycosidase digested SERPINA1 complexed with elastase. The major glycoforms suitable for native MS quantification are labelled in red. We highlighted two pairs of unresolvable glycoforms in yellow and green, respectively. Native MS cannot resolve two 74 kD proteins differing with 8 Da [14]. Therefore, we cannot accurately quantify fucosylation regulation to double exoglycosidase treated SERPINA1-elastase complexes using native MS analysis.

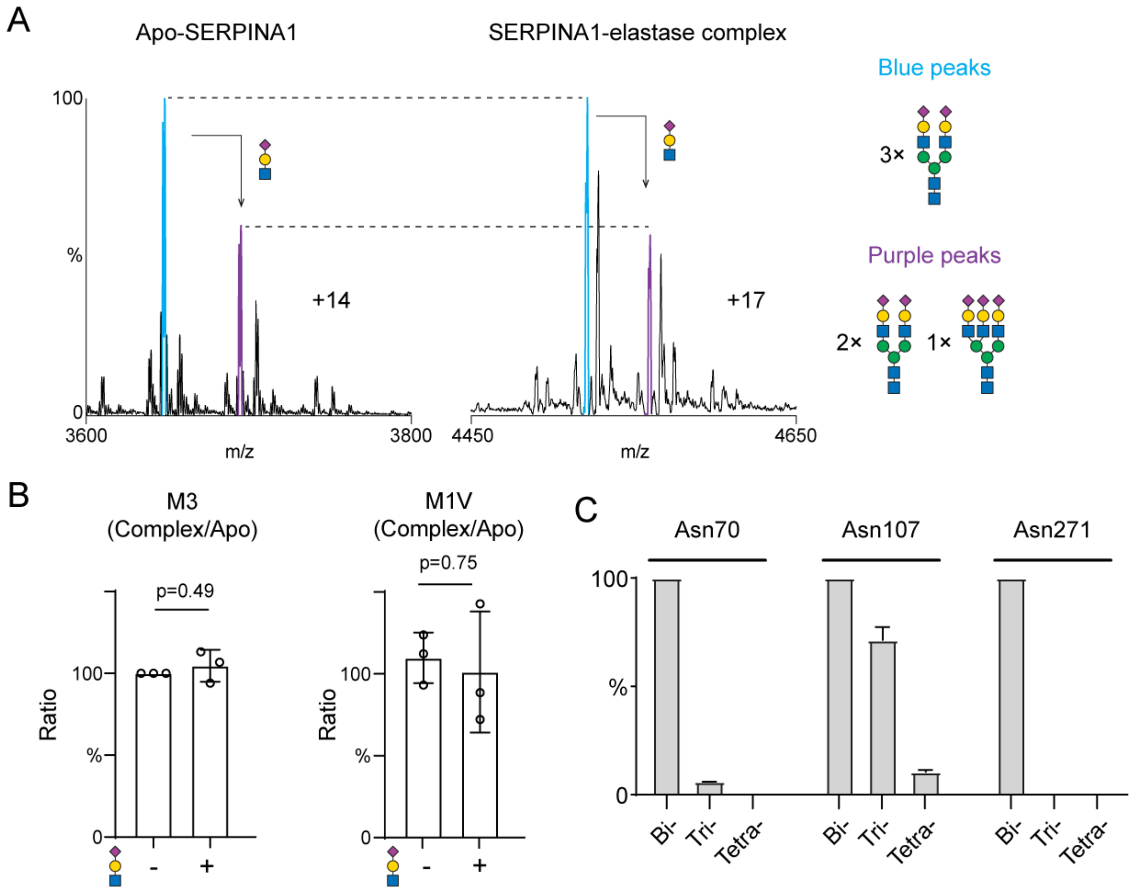

Figure S12. MS analysis of N-glycan branching regulation to SERPINA1-elastase interactions. A) Native mass spectrum of apo-SERPINA1 and SERPINA1-elastase complexes. The peaks are labelled with the corresponding charge states. apo-SERPINA1 and SERPINA1-elastase complex peaks with three bi-antennary N-glycans are highlighted in blue. The peaks with two bi-antennary and one tri-antennary N-glycan are highlighted in purple. B) Bar graphs of the ratio of the SERPINA1-elastase complex to apo-SERPINA1, with and without the tri-saccharide Neu5Ac-Gal-GlcNAc unit. Bars show mean ± standard deviation with dots from three independent experiments. A student's t-test was performed to calculate the p value. C) Glycoproteomics analysis of the site-specific N-glycan branching level. Bar graphs of the relative abundance of bi-, tri- and tetra- antennary N-glycans at Asn70, Asn107 and Asn271. Bars show mean ± standard deviation from three independent glycoproteomics experiments.

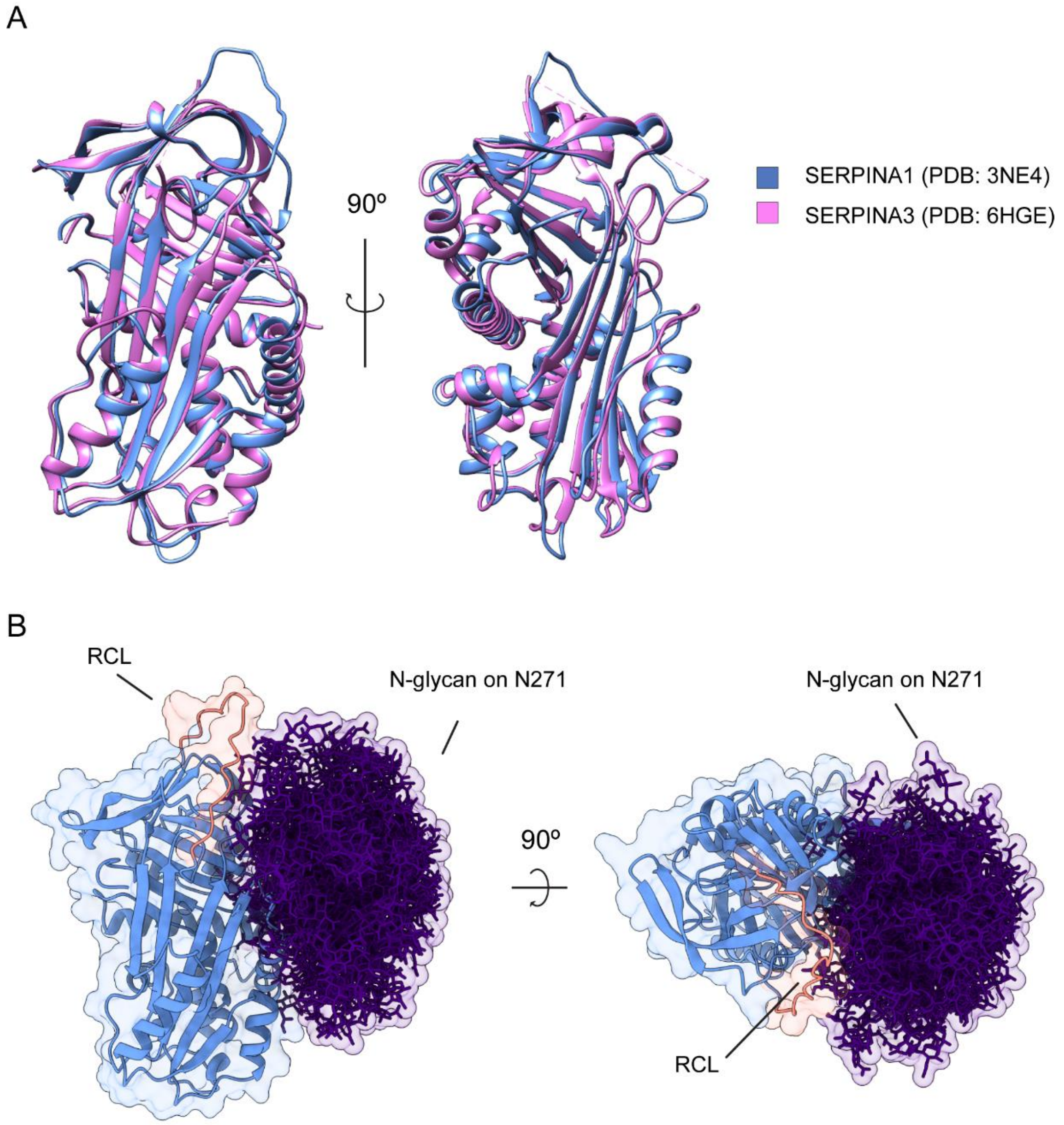

Figure S13. Structural analysis of SERPINA3. A) Structural comparison of SERPINA1 and SERPINA3. The structure superimposing of SERPINA1 (PDB: 3NE4) and SERPINA3 (PDB: 6HGE) were performed using UCSF Chimera program. B) The relative positions of reactive center loop (RCL) and N-glycan on Asn271 in SERPINA3. The RCL is highlighted in orange. The N-glycan conformers on Asn271 were simulated with GlycoSHIELD program using a sialylated bi-antennary N-glycan as a model. The possible N-glycan conformers on Asn271 are overlayed and shown in purple.

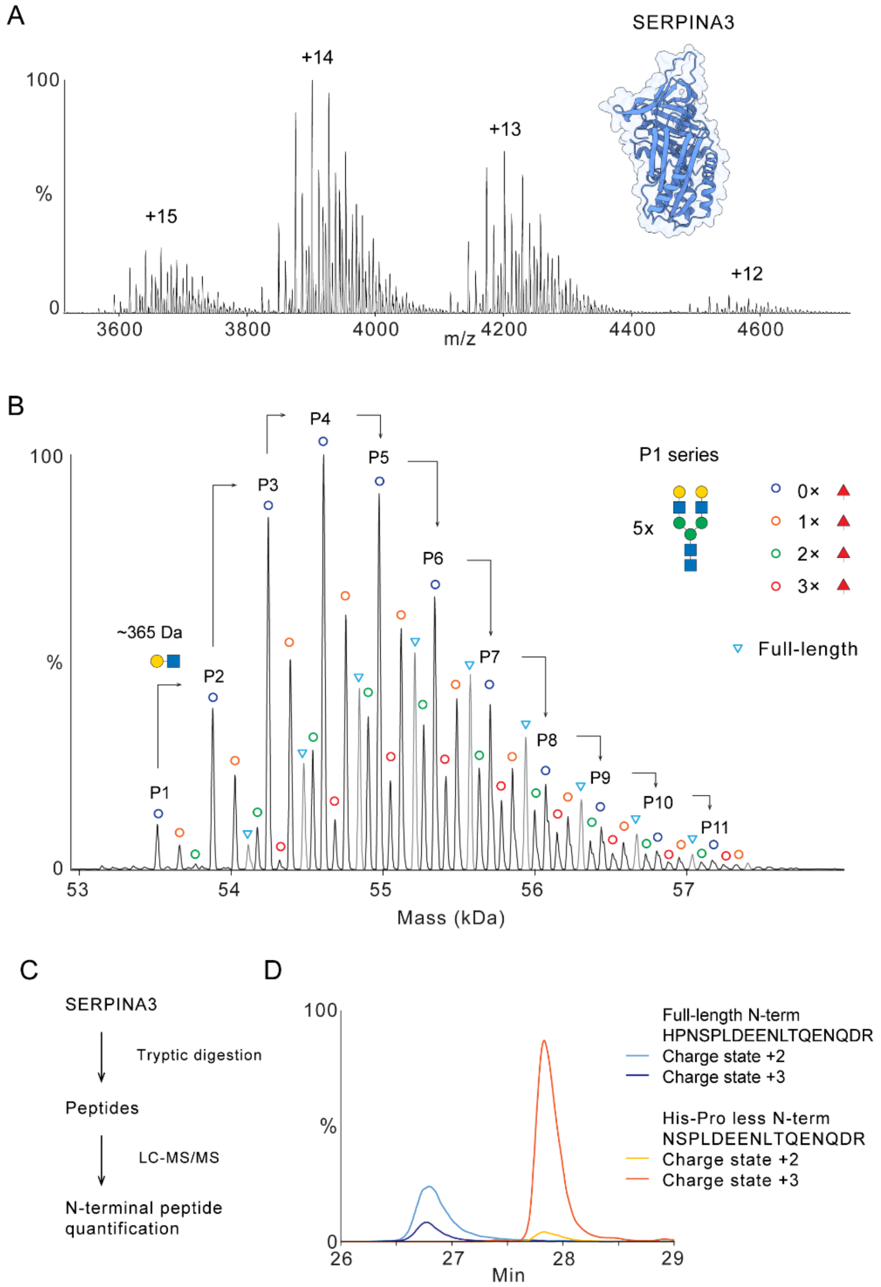

Figure S14. MS analysis of SERPINA3. A) Native mass spectrum of desialylated SERPINA3. B) Annotation of the desialylated SERPINA3 proteoforms. The N-glycan branching (addition of Gal-GlcNAc units, P1 to P11 peak series) and fucosylation (addition of fucose residues) are labelled. C) The flow chart of proteomics analysis of SERPINA3 N-terminal truncation. D) The extracted ion chromatograms of the tryptic peptides from full-length and N-terminal truncated (His-Pro less variant) SERPINA1.

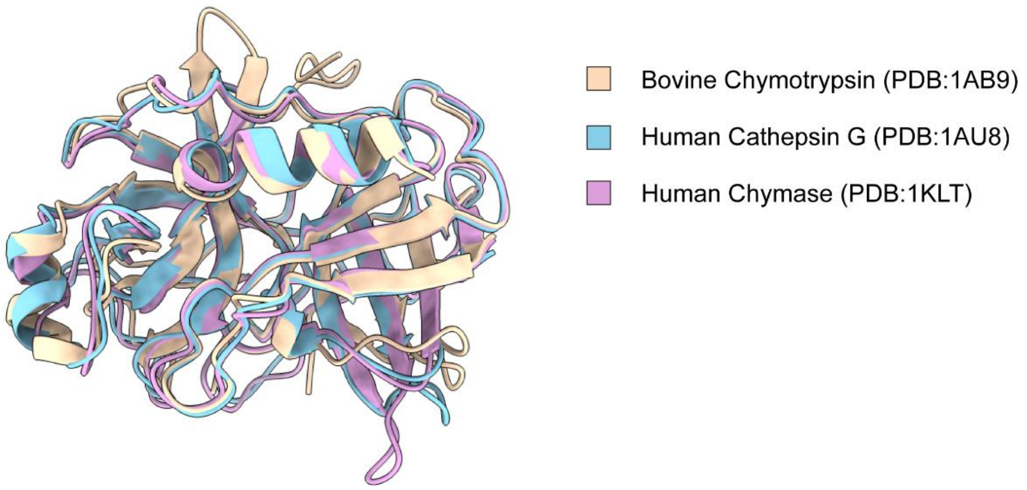

Figure S15. Structural comparison of bovine chymotrypsin (PDB:1AB9), human cathepsin G (PDB: 1AU8) and human chymase (PDB: 1KLT). The structural alignment was performed using UCSF Chimera program.

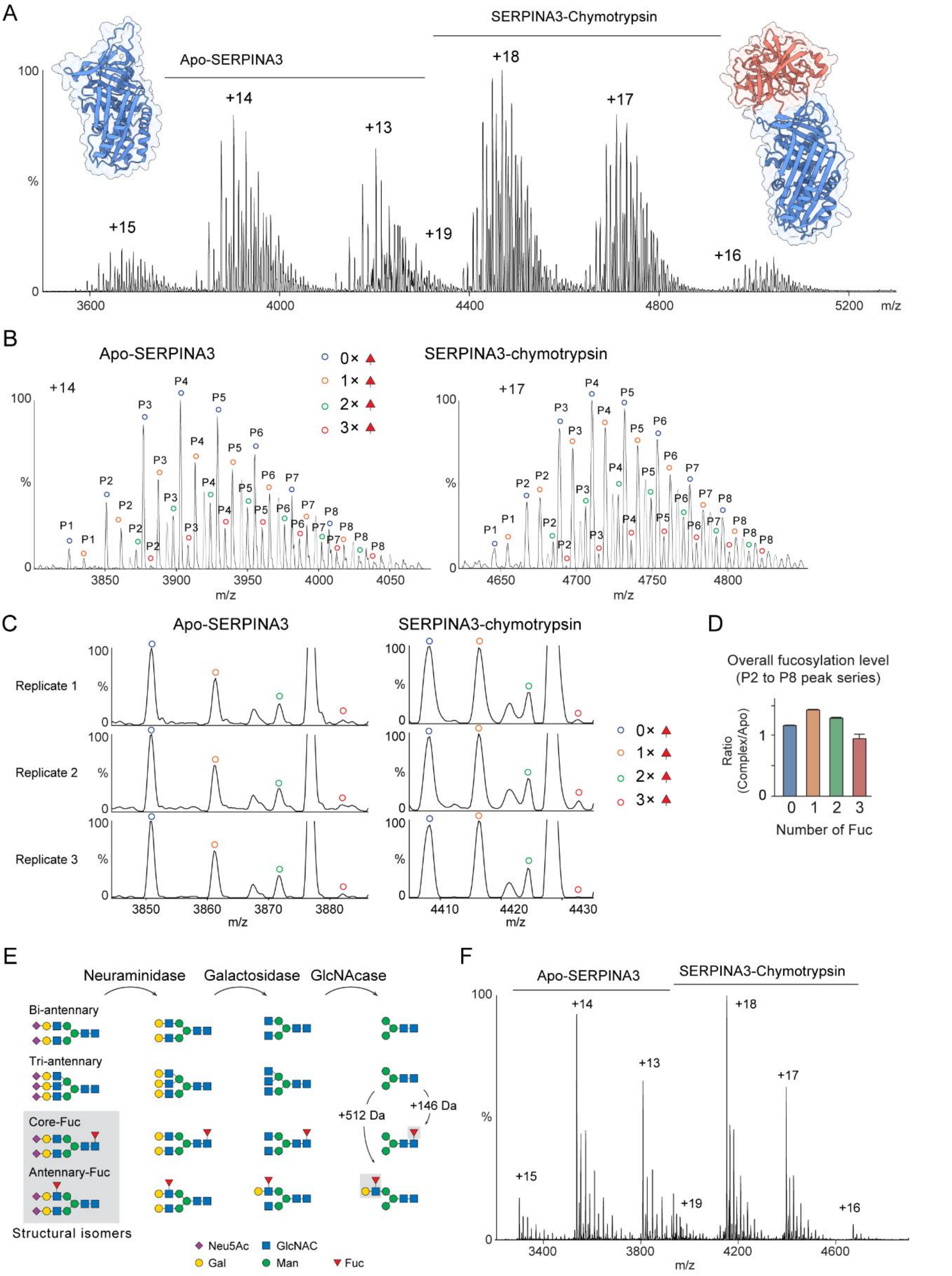

Figure S16. Native MS analysis of SERPINA3-chymotrypsin complexes. A) Native mass spectrum of SERPINA3-chymotrypsin complexes. B) annotation of the proteoforms of SERPINA3 and SERPINA3-chymotrypsin complexes. Fucosylation statuses, namely non-, mono-, bi- and tri- fucosylation are labelled with blue, orange, green and red circles, respectively. Peaks with the same hexose (Hex) and

N-acetylhexosamine (HexNAc) numbers are labeled P1 to P8. C) Native mass spectra of P2 series of apo-SERPINA3 and SERPINA3-chymotrypsin complexes. The spectra from three replicates are shown. Fucosylation statuses, namely non-, mono-, bi- and tri- fucosylation are labelled with blue, orange, green and red circles, respectively. D) Bar graph of the ratio of the complex to apo protein with 0 to 3 fucose residues. Bars show mean±s.d. from three independent experiments. E) Triple exoglycosidase treatment of SERPINA3 to distinguish core- and antennary fucosylation. F) The native MS spectrum of triple exoglycosidase treated SERIPNA3 and SERPINA3-chymotrypsin complexes.

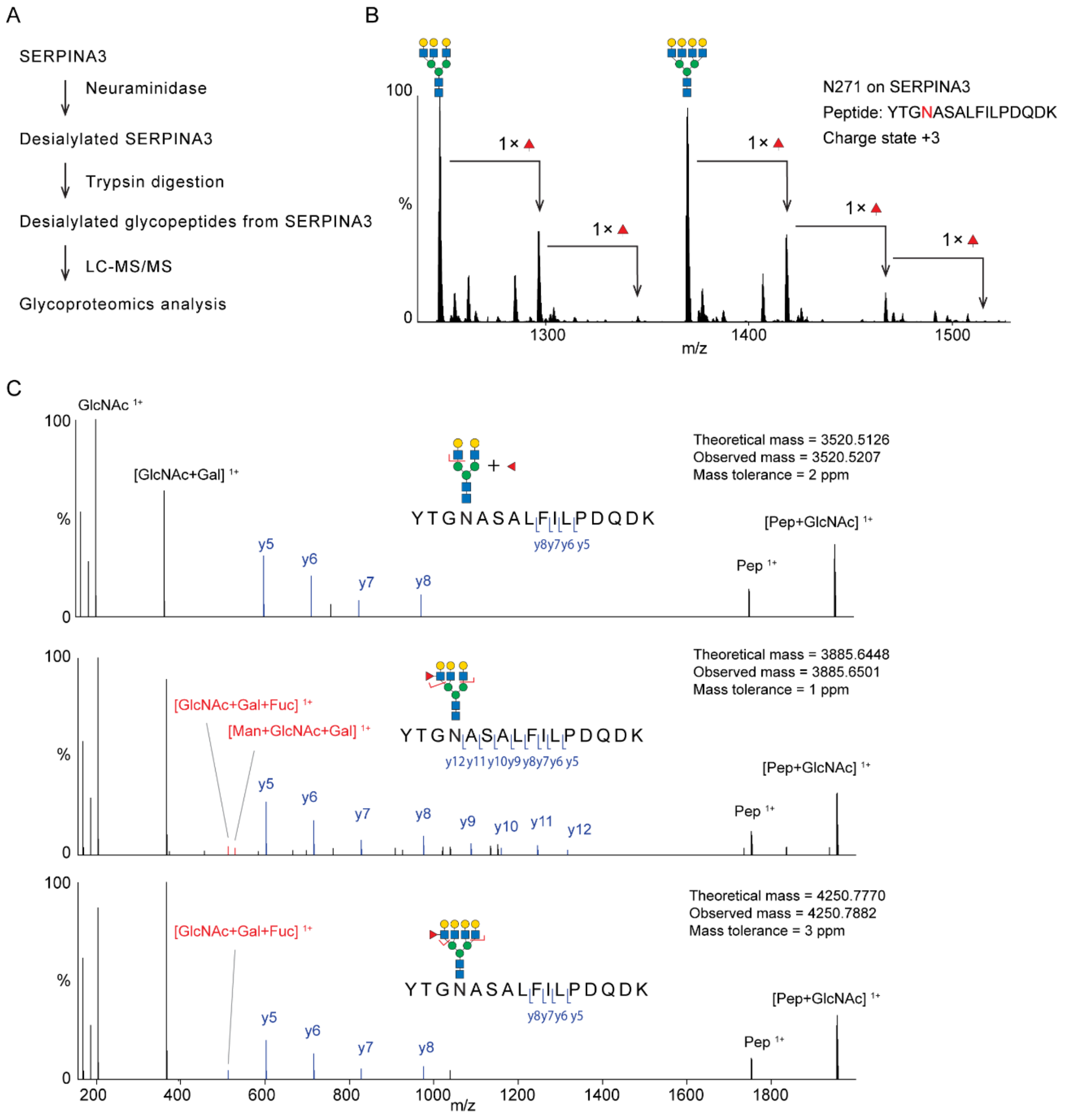

Figure S17. Glycoproteomics analysis of SERPINA3. A) Flow chart of the glycoproteomics analysis of desialylated SERPINA3. B) The mass spectrum of the glycopeptide (YTGNASALFILPDQDK) primarily carries tri- and tetra- antennary N-glycans without/with fucose residues on Asn271. C)

Identification of mono-fucosylated bi-, tri- and tetra- antennary N-glycans on Asn271. We identified the diagnostic ion of $[GlcNAc+Gal+Fuc]^{1+}$ in the MS/MS spectra of Asn271 peptide with tri- and tetra-antennary N-glycans. This suggests the presence of antennary fucosylation on tri- and tetra- antennary N-glycans. However, we cannot rule out the presence of core-fucosylation on tri- and tetra-antennary N-glycans. Moreover, we didn't observe this diagnostic ion in the MS/MS spectrum of Asn271 peptide with bi-antennary N-glycan. It implies that the bi-antennary N-glycan on Asn271 may be principally core-fucosylated.

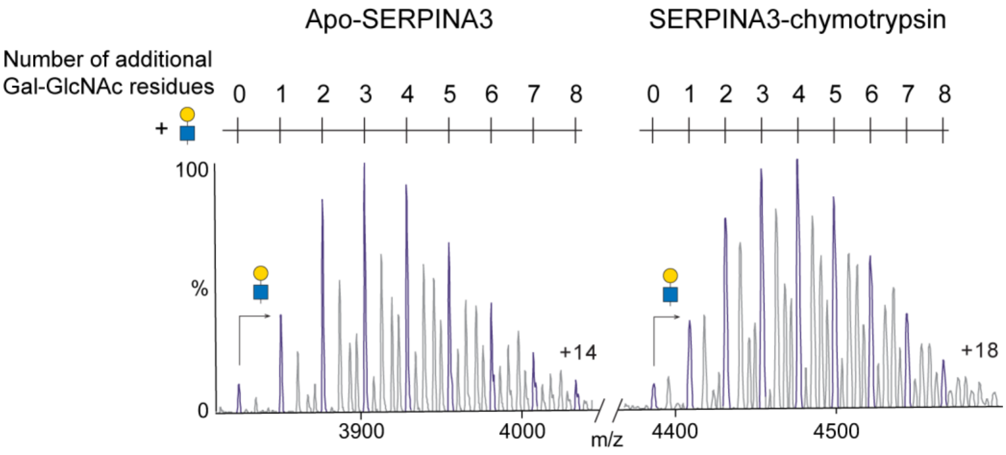

Figure S18. Annotation of N-glycan branching on apo-SERPINA3 and SERPINA3-chymotrypsin complexes.

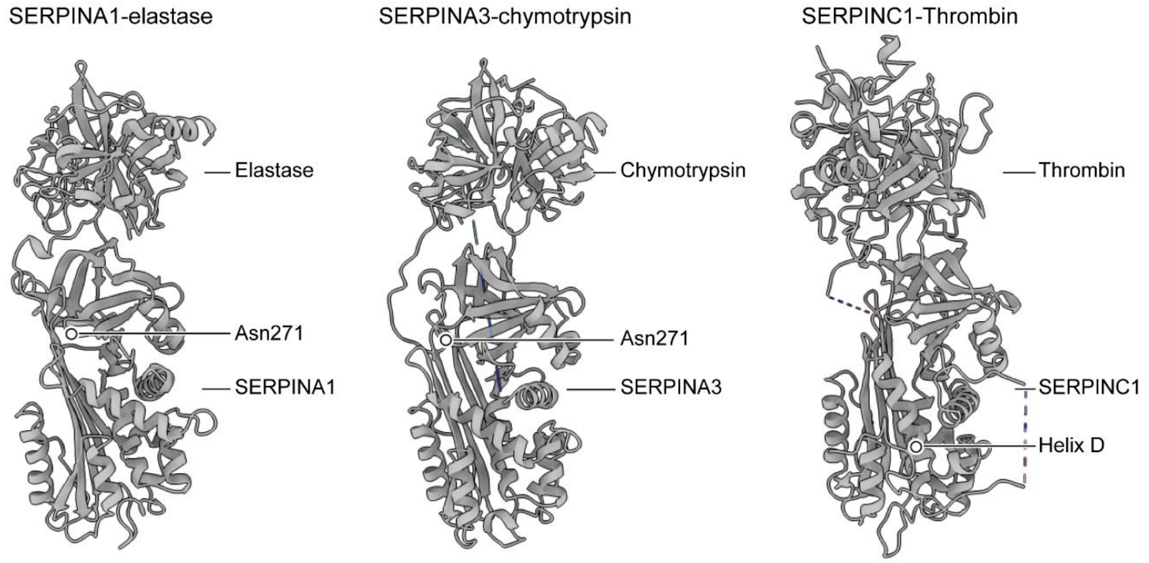

Figure S19. Structures of SERPINA1-elastase, SERPINA3-chymotrypsin, and SERPINC1-thrombin. The structures of SERPINA1-elastase and SERPINA3-chymotrypsin complexes were modelled using SERPINA1 (PDB: 3NE4), elastase (PDB: 1BMA), SERPINA3 (PDB: 6HGE) and chymotrypsin (PDB: 4CHA) using SERPINA1-trypsin complex (PDB: 1OPH) as a template using UCSF Chimera program. The structure of SERPINC1-thrombin was extracted from Thrombin-SERPINC1-heparin complex (PDB: 1TB6). The Asn271 in SERPINA1 and SERPINA3, and the helix D in SERPINC1 are highlighted, respectively.

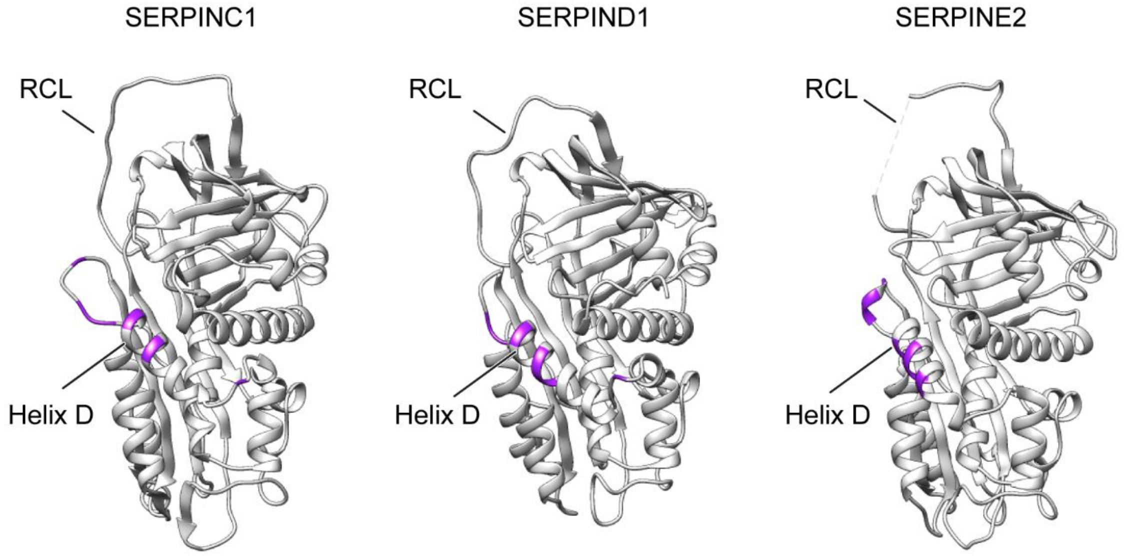

Figure S20. Structures of SERPINC1 (PDB:1OPH), SERPIND1 (PDB: 1JMJ) and SERPINE2 (PDB: 4DY0). The positively charged amino acids in helix D are highlighted in pink.

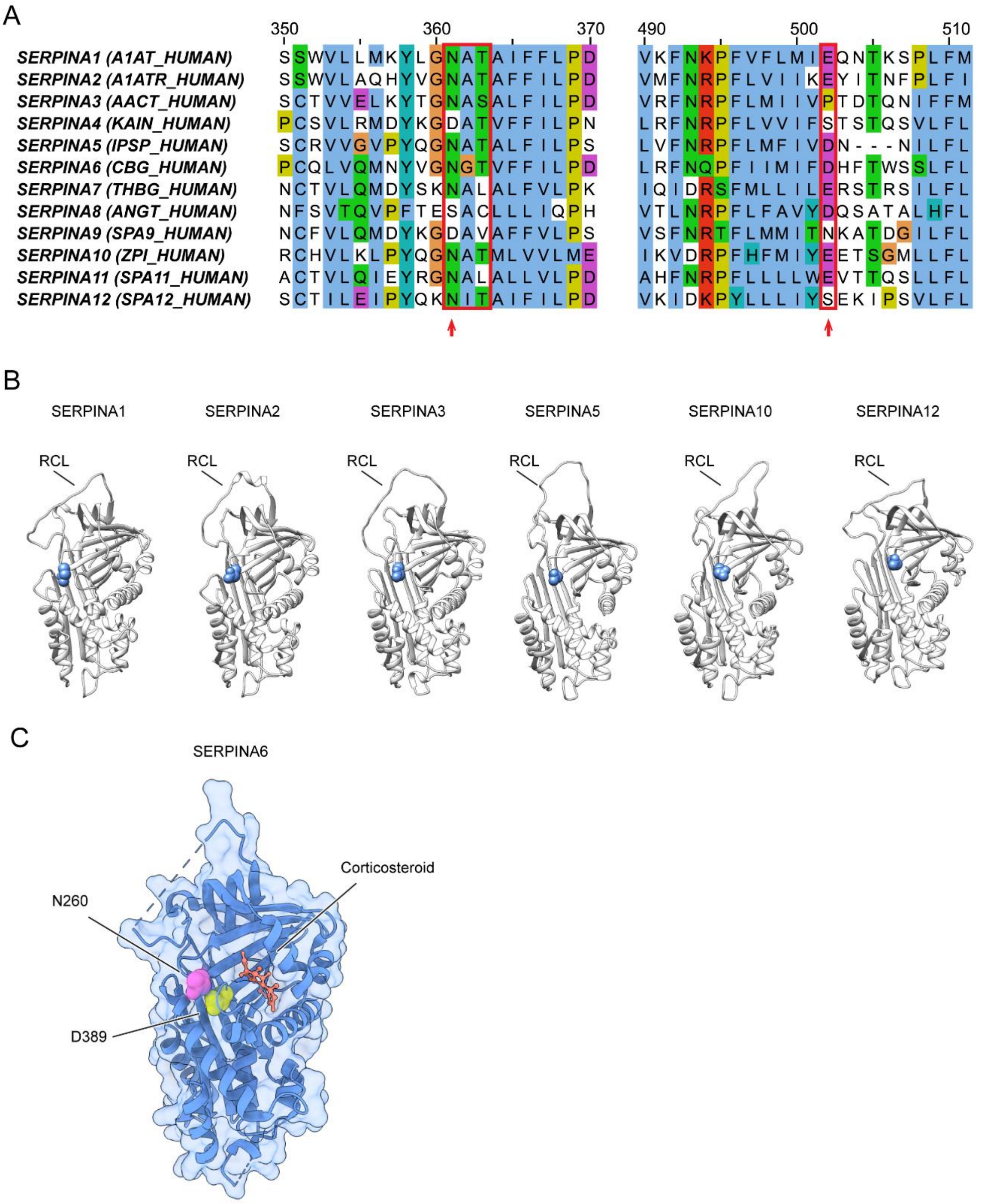

Figure S21. Structural analysis of SERPINs. A) Sequence alignment of the twelve members in SERPIN clade A. The sequence alignment was performed using Jalview program (B) Structures of SERPINA1, A2, A3, A5, A6 and A12. The N-glycosylation sites corresponding to N271 in SERPINA1/A3 are highlighted in blue. The SERPIN structures were retrieved from AlphaFold protein structural database. C) Structure of SERPINA6 (PDB: 2V95). The N260 (corresponding to N271 in SERPINA1/A3) is highlighted in pink. The D389 (corresponding to E400 in SERPINA1) is highlighted in yellow. The ligand, corticosteroid is in orange.

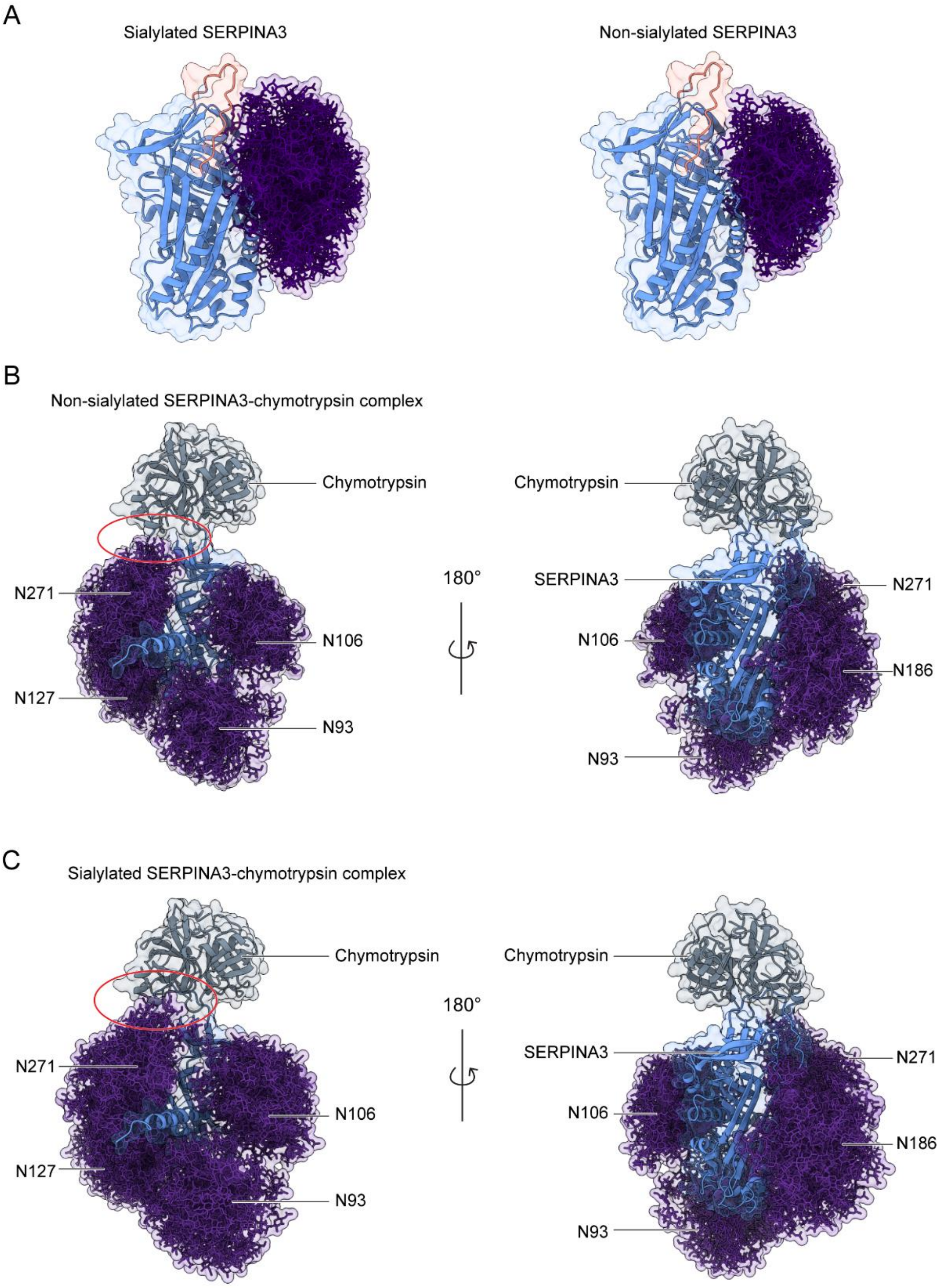

Figure S22. Structural analysis of glycosylated SERPINA3-chymotrypsin complexes. A) SERPINA3 with possible conformers of sialylated bi-antennary N-glycan and non-sialylated bi-antennary N-glycan on Asn271. The SERPINA3-chymotrypsin complexes with possible conformers of non-sialylated and sialylated bi-antennary N-glycans are shown in panel B and C, respectively. The red circles highlight that some N-glycan conformers on Asn271 are proximal to chymotrypsin.

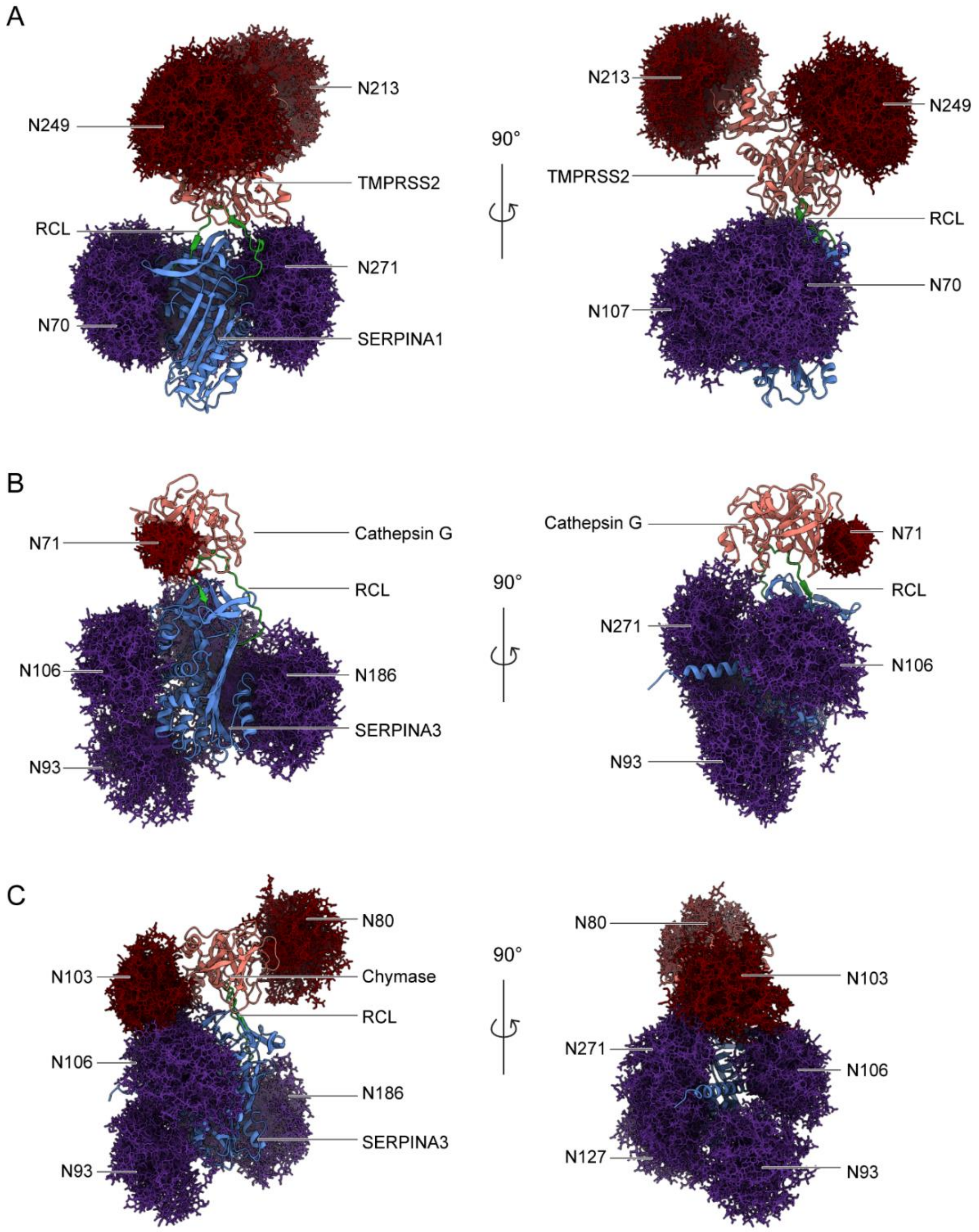

Figure S23. Modelling of the interactions between SERPINA1-TMPRSS2, SERPINA3-Cathepsin G and SERPINA3-Chymase complexes with all possible conformers of the sialylated bi-antennary N-glycans (panels A, B and C, respectively). Sialylated bi-antennary N-glycans were modelled at Asn70, Asn107 and Asn271 in SERPINA1, and Asn93, Asn106, Asn127, Asn186 and Asn271 in SERPINA3. According to previous biochemical studies, sialylated bi-antennary N-glycans were modelled at Asn213 and Asn219 in TMPRSS2 extracellular domain [15], and Asn80 and Asn103 in Chymase [16]. A small M2 glycan (Manα1-6Manβ1-4GlcNAcβ1-4GlcNAcβ) was modelled at Asn71 in Cathepsin G [17]. The N-glycans on TMPRSS2, Cathepsin G and Chymase are highlighted in dark red. The N-glycans on SERPINA1 and SERPINA3 are labelled in dark purple. The RCLs in SERPINA1 (Gly368 to Lys392) and SERPINA3 (Gly369 to Arg394) are highlighted in green in all models.

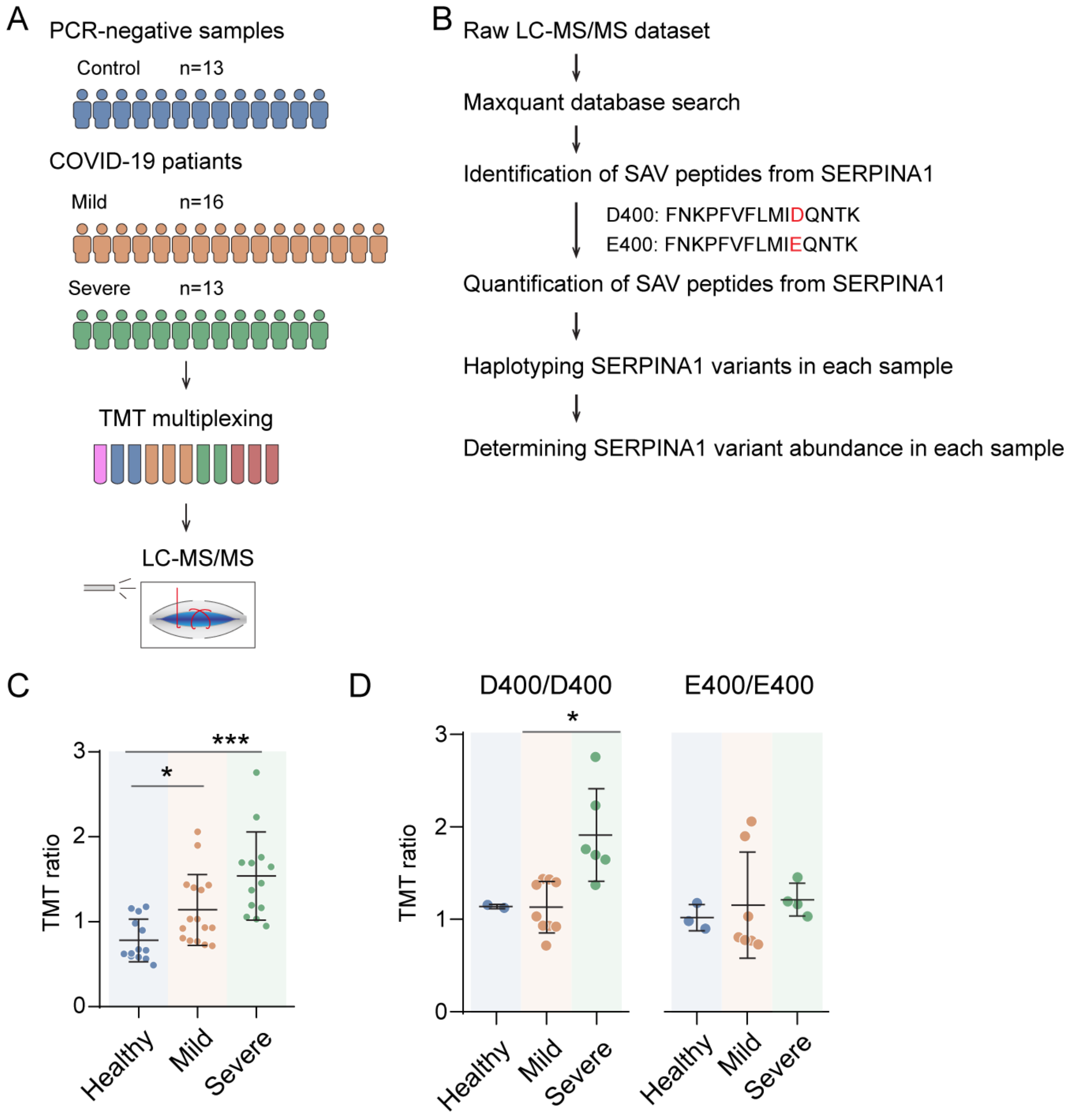

Figure S24. Analysis of SERPINA1 single amino acid variants (SAVs) in COVID-19. A) Overview of plasma sample information and proteomics workflow of COVID-19 patients, including 16 individuals with mild symptoms and 13 individuals with severe symptoms. Analysis of these plasma samples was performed by another research group using trypsin digestion, labelling with TMT 11-plex, fractionation and analysis with LC-MS/MS and reported elsewhere [13]. B) SAV analysis of a COVID-19 proteomics dataset. Briefly, we processed the LC-MS/MS raw data with Maxquant using a tailored human plasma protein database with SAV information. We identified SAV peptides from SERPINA1 (E400 and D400) and quantified their abundances. We performed phasing of E400 and D400 based on the relative abundances (TMT ratio) of the corresponding peptides in each sample. Detailed data processing steps are described in the Supplementary Methods. C) Relative abundances of SERPINA1 in healthy, mild and severe groups. Means and standard deviations of three groups were plotted (dots represent data from each sample). Nonparametric Mann-Whitney tests were performed to calculate p value (*, $p<0.05$; ***, $p<0.001$). Bonferroni correction was applied for post hoc analysis. D) Relative abundances of

SERPINA1$^{D400/D400}$ and SERPINA1$^{E400/E400}$ variants in healthy, mild and severe groups. Plots represent the means and standard deviations of three groups with dots representing data from each individual. Nonparametric Mann-Whitney tests were performed to calculate p value (*, $p<0.05$). Bonferroni correction was performed for post hoc analysis.

Supplemental references

1. Chen, S., Wu, D., Robinson, C. V, and Struwe, W.B. (2021). Native Mass Spectrometry Meets Glycomics: Resolving Structural Detail and Occupancy of Glycans on Intact Glycoproteins. Anal. Chem. *93*, 10435–10443. 10.1021/acs.analchem.1c01460.

2. Cox, J., Neuhauser, N., Michalski, A., Scheltema, R.A., Olsen, J. V, and Mann, M. (2011). Andromeda: a peptide search engine integrated into the MaxQuant environment. J. Proteome Res. *10*, 1794–1805. 10.1021/pr101065j.

3. Liu, M.-Q., Zeng, W.-F., Fang, P., Cao, W.-Q., Liu, C., Yan, G.-Q., Zhang, Y., Peng, C., Wu, J.-Q., Zhang, X.-J., et al. (2017). pGlyco 2.0 enables precision N-glycoproteomics with comprehensive quality control and one-step mass spectrometry for intact glycopeptide identification. Nat. Commun. *8*, 438. 10.1038/s41467-017-00535-2.

4. Pettersen, E.F., Goddard, T.D., Huang, C.C., Couch, G.S., Greenblatt, D.M., Meng, E.C., and Ferrin, T.E. (2004). UCSF Chimera--a visualization system for exploratory research and analysis. J. Comput. Chem. *25*, 1605–1612. 10.1002/jcc.20084.

5. Waterhouse, A.M., Procter, J.B., Martin, D.M.A., Clamp, M., and Barton, G.J. (2009). Jalview Version 2--a multiple sequence alignment editor and analysis workbench. Bioinformatics *25*, 1189–1191. 10.1093/bioinformatics/btp033.

6. Notredame, C., Higgins, D.G., and Heringa, J. (2000). T-coffee: A novel method for fast and accurate multiple sequence alignment. J. Mol. Biol. *302*, 205–217. 10.1006/jmbi.2000.4042.

7. Jo, S., Kim, T., Iyer, V.G., and Im, W. (2008). CHARMM-GUI: A web-based graphical user interface for CHARMM. J. Comput. Chem. *29*, 1859–1865. 10.1002/jcc.20945.

8. Lee, J., Cheng, X., Swails, J.M., Yeom, M.S., Eastman, P.K., Lemkul, J.A., Wei, S., Buckner, J., Jeong, J.C., Qi, Y., et al. (2016). CHARMM-GUI Input Generator for NAMD, GROMACS, AMBER, OpenMM, and CHARMM/OpenMM Simulations Using the CHARMM36 Additive Force Field. J. Chem. Theory Comput. *12*, 405–413. 10.1021/acs.jctc.5b00935.

9. Park, S.J., Lee, J., Qi, Y., Kern, N.R., Lee, H.S., Jo, S., Joung, I., Joo, K., Lee, J., and Im, W. (2019). CHARMM-GUI Glycan Modeler for modeling and simulation of carbohydrates and

glycoconjugates. Glycobiology *29*, 320–331. 10.1093/glycob/cwz003.

10. Abraham, M.J., Murtola, T., Schulz, R., Páll, S., Smith, J.C., Hess, B., and Lindahl, E. (2015). GROMACS: High performance molecular simulations through multi-level parallelism from laptops to supercomputers. SoftwareX *1–2*, 19–25. 10.1016/j.softx.2015.06.001.

11. Humphrey, W., Dalke, A., and Schulten, K. (1996). VMD: Visual molecular dynamics. J. Mol. Graph. *14*, 33–38. 10.1016/0263-7855(96)00018-5.

12. Gecht, M., von Bülow, S., Penet, C., Hummer, G., Hanus, C., and Sikora, M. (2021). GlycoSHIELD: a versatile pipeline to assess glycan impact on protein structures. Preprint at bioRxiv, 2021.08.04.455134. 10.1101/2021.08.04.455134.

13. Shu, T., Ning, W., Wu, D., Xu, J., Han, Q., Huang, M., Zou, X., Yang, Q., Yuan, Y., Bie, Y., et al. (2020). Plasma Proteomics Identify Biomarkers and Pathogenesis of COVID-19. Immunity *53*, 1108-1122.e5. 10.1016/j.immuni.2020.10.008.

14. Lössl, P., Snijder, J., and Heck, A.J.R. (2014). Boundaries of Mass Resolution in Native Mass Spectrometry. J. Am. Soc. Mass Spectrom. *25*, 906–917. 10.1007/s13361-014-0874-3.

15. Chen, Y.-W., Lee, M.-S., Lucht, A., Chou, F.-P., Huang, W., Havighurst, T.C., Kim, K., Wang, J.-K., Antalis, T.M., Johnson, M.D., et al. (2010). TMPRSS2, a Serine Protease Expressed in the Prostate on the Apical Surface of Luminal Epithelial Cells and Released into Semen in Prostasomes, Is Misregulated in Prostate Cancer Cells. Am. J. Pathol. *176*, 2986–2996. 10.2353/ajpath.2010.090665.

16. Chen, R., Jiang, X., Sun, D., Han, G., Wang, F., Ye, M., Wang, L., and Zou, H. (2009). Glycoproteomics analysis of human liver tissue by combination of multiple enzyme digestion and hydrazide chemistry. J. Proteome Res. *8*, 651–661. 10.1021/pr8008012.

17. Loke, I., Packer, N., and Thaysen-Andersen, M. (2015). Complementary LC-MS/MS-Based N-Glycan, N-Glycopeptide, and Intact N-Glycoprotein Profiling Reveals Unconventional Asn71-Glycosylation of Human Neutrophil Cathepsin G. Biomolecules *5*, 1832–1854. 10.3390/biom5031832.
