# Supplementary material for: Connecting single-nucleotide polymorphisms, glycosylation status, and interactions of plasma serine protease inhibitors
Source: Chem. 2023 Mar 9;9(3):665–81. doi: 10.1016/j.chempr.2022.11.018 (PMC10914678; doi:10.1016/j.chempr.2022.11.018)
Supplement: Document S2. Article plus supplemental information [file mmc2.pdf]

Chem

CellPress
OPEN ACCESS

Article

Connecting single-nucleotide polymorphisms, glycosylation status, and interactions of plasma serine protease inhibitors

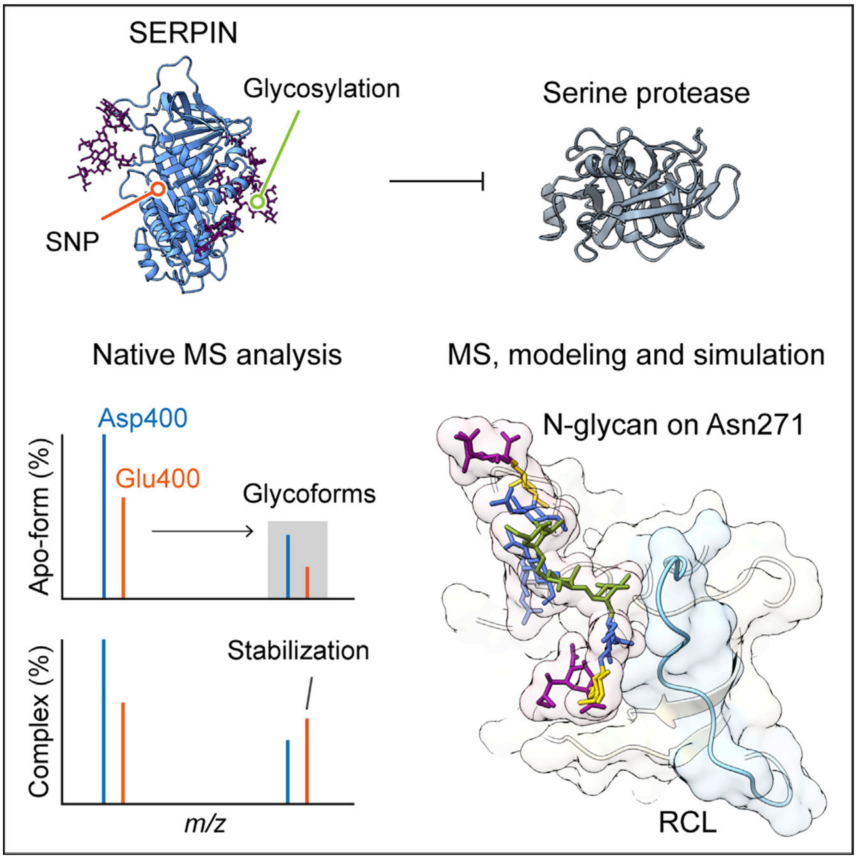

Di Wu, Manman Guo, Carol V. Robinson

carol.robinson@chem.ox.ac.uk

Highlights

Native mass spectrometry uncovers proteoform and SNP-specific protein interactions

Core and antennary fucosylation play opposing roles in SERPIN interactions

Core fucosylation and E400D mutation cooperatively modulates SERPINA1 interactions

Understanding the combined effects of single-nucleotide polymorphisms (SNPs) and post-translational modifications (PTMs) on protein interactions is challenging. Wu et al. developed and applied a high-resolution native mass spectrometry (MS) approach to monitor individual SNPs and glycoforms on serine protease inhibitors (SERPINs) in complexes with proteases. To do this, they combined MS, modeling, and molecular dynamics simulations to relate site-specific glycan heterogeneity to SERPIN-protease interactions.

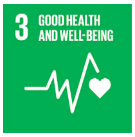

Wu et al., Chem 9, 665–681
March 9, 2023 © 2022 The Authors. Published by Elsevier Inc.
https://doi.org/10.1016/j.chempr.2022.11.018

Chem

CellPress
OPEN ACCESS

Article

Connecting single-nucleotide polymorphisms, glycosylation status, and interactions of plasma serine protease inhibitors

Di Wu,[1,2] Manman Guo,[3] and Carol V. Robinson[1,2,4,*]

SUMMARY

Understanding the combined impacts of genetic variances and post-translational modifications requires new approaches. Here, we delineate proteoforms of plasma serine protease inhibitors and relate specific proteoforms to their interactions in complexes through the use of native mass spectrometry (MS). First, we dissect the proteoform repertoire of an acute-phase plasma protein, serine protease inhibitor A1 (SERPINA1), resolving four SERPINA1 variants (M1V, M1A, M2, and M3) with common single-nucleotide polymorphisms (SNPs). Investigating the glycosylation status of these variants and their ability to form complexes with a serine protease, elastase, we find that fucosylation stabilizes the interaction of the SERPINA1 M1V variant through its core fucosylation on Asn271. In contrast, antennary fucosylation on Asn271 destabilizes SERPINA1-elastase interactions. We unveil the same opposing effects of core and antennary fucosylation on SERPINA3 interactions with chymotrypsin. Together, our native MS results highlight the modulating effects of fucosylation with different linkages on glycoprotein interactions.

THE BIGGER PICTURE

The most common type of genetic variation among people is single-nucleotide polymorphisms (SNPs). Occurring throughout a person's DNA, SNPs can influence modifications on proteins (post-translational modifications [PTMs]). The combination of genetic variance and protein modification results in myriad variations. Understanding the combined impact of these two variations on protein activity and function is challenging.

Selecting serine proteases due to their roles in diverse physiological processes from blood coagulation to immunity, researchers considered how their activity is regulated by binding to inhibitory proteins (serine protease inhibitors [SERPINs]). Maintaining SERPIN-protease complexes intact and characterizing their PTMs/SNPs required high-resolution mass spectrometry. By developing such an approach, the authors revealed synergistic effects of SNPs and PTMs on SERPIN-protease interactions. More broadly, this approach connects genomic discovery with protein function.

INTRODUCTION

Proteoforms are distinct variants of protein molecules, derived from combinations of individual genetic variances, translational, and post-translational modifications (PTMs).[1] Several diseases are driven by aberrant expression of these proteoforms making it imperative to understand how proteoforms regulate protein interactions and influence downstream signaling events. Recent genomics and proteomics studies discovered thousands of disease-related proteoforms and informed the importance of proteoforms in personalized medicine and oncology.[2,3] A wealth of information exists for disease-related proteoforms in human blood,[4] and many plasma protein proteoforms have been implicated in the predisposition or onset of disease.[5,6] However, missing to date is the link between aberrant proteoforms or SNPs, their glycosylation status and how these combine to regulate plasma protein interactions.

Human serine protease inhibitors (SERPINs) belong to a family of 37 evolutionarily conserved members that regulate the activity of over 180 serine proteases.[7] Therefore, the interplay of SERPINs and serine proteases controls many biological processes, including immune responses, coagulation, and cell growth/maturation. Notably, the expression levels and PTMs of SERPINs serve as biomarkers for susceptibility and pathogenesis of lung cancer, hepatocellular carcinoma, and coronavirus disease 2019 (COVID-19).[6,8,9] Moreover, recent genome-wide association studies

This is an open access article under the CC BY license (http://creativecommons.org/licenses/by/4.0/).

(GWASs) demonstrated that nonsynonymous mutations of SERPINs are related to their expression levels, function, and metabolic interactome.[5,10]

A number of SERPINs are present in human plasma, and all are structurally similar with two main features: a five-stranded β-sheet A and a flexible reactive center loop (RCL) containing a protease cleavage site. The two most abundant plasma SERPINs, SERPINA1 (α1-antitrypsin) and SERPINA3 (α1-antichymotrypsin) are acute-phase proteins, upregulated during inflammation.[7] SERPINA1 is the major endogenous inhibitor of elastase, a serine protease secreted by activated neutrophils to degrade foreign molecules and microorganisms.[11] SERPINA1 is also the endogenous inhibitor of the transmembrane protease serine 2 (TMPRSS2), critical for spike protein shedding on the SARS-CoV-2 virion.[12] SERPINA3 is an important regulator of the renin-angiotensin system and neutralizes mast cell chymase and neutrophil cathepsin G activities.[13]

More than a hundred SERPINA1 variants have been identified, many of which are related to α1-antitrypsin deficiency (AATD), an autosomal-codominant disorder characterized by reduced protein levels in plasma. Conflicting reports exist however about whether other common variants are benign or pathogenic with these discrepancies arising from genomics, biochemical and biophysical observations.[5,10,14,15] Genomics studies revealed associations of SNPs in SERPINA1 to specific diseases. Nevertheless, SERPINA1 has three heterogeneous covalently linked N-glycans, with differences in occupancy, composition, linkage, and structure that are anticipated to fine-tune protein structure and function.[16] The apparent activity and function of endogenous SERPINA1, containing all proteoforms, is therefore likely highly variable. While recombinant protein expression eliminates this variability, proteins expressed in immortalized cell lines are often different from their endogenous counterparts derived from human plasma/tissue.[17] Therefore, capturing proteoform-specific interactions of endogenous proteins from human plasma/tissue is critical in connecting genomic discoveries to protein function.

Mass spectrometry (MS)-based proteomics, involving sequencing of either digested or intact proteins, plays an important role in proteoform discovery, particularly in identifying novel PTMs[18] and unveiling the regulation of biological systems.[19] Proteomics is not yet capable of directly informing proteoform-specific interactions.[20] Recently, native MS has demonstrated great promise in defining the extent of proteoforms within protein complexes.[21] Here, we investigated how SNPs and PTMs synergize to impact SERPIN interactions using a native MS approach.

RESULTS

Resolving SERPINA1 proteoforms by high-resolution native MS

We began our investigation using human plasma directly from a single donor on a modified Orbitrap Eclipse mass spectrometer.[22] Since SERPINA1 is an abundant plasma glycoprotein and the *SERPINA1* gene is highly polymorphic, we anticipated detection of one or more of the twenty frequently observed SNPs in the coding exon region. The M1V (canonical sequence), M1A (Ala237), M2 (His125/Asp400), and M3 (Asp400) are the four most common variants, encoded by wild-type *SERPINA1* sequences (Figure 1A). SERPINA1 also carries three N-glycosylation sites at Asn70, Asn107, and Asn271, and a cysteinylation site at C256 (Figure 1B). The serine protease cleavage site (the cleavage occurs between Met382 and Ser383) is located on the RCL (Gly368 to Lys392). The native mass spectrum of this donor plasma is

[1]Department of Chemistry, University of Oxford, Oxford OX1 3QZ, UK
[2]Kavli Institute for Nanoscience Discovery, University of Oxford, Oxford OX1 3QU, UK
[3]Botnar Research Centre, NIHR Biomedical Research Unit Oxford, Nuffield Department of Musculoskeletal Sciences, University of Oxford, Oxford OX3 7LD, UK
[4]Lead contact
*Correspondence: carol.robinson@chem.ox.ac.uk
https://doi.org/10.1016/j.chempr.2022.11.018

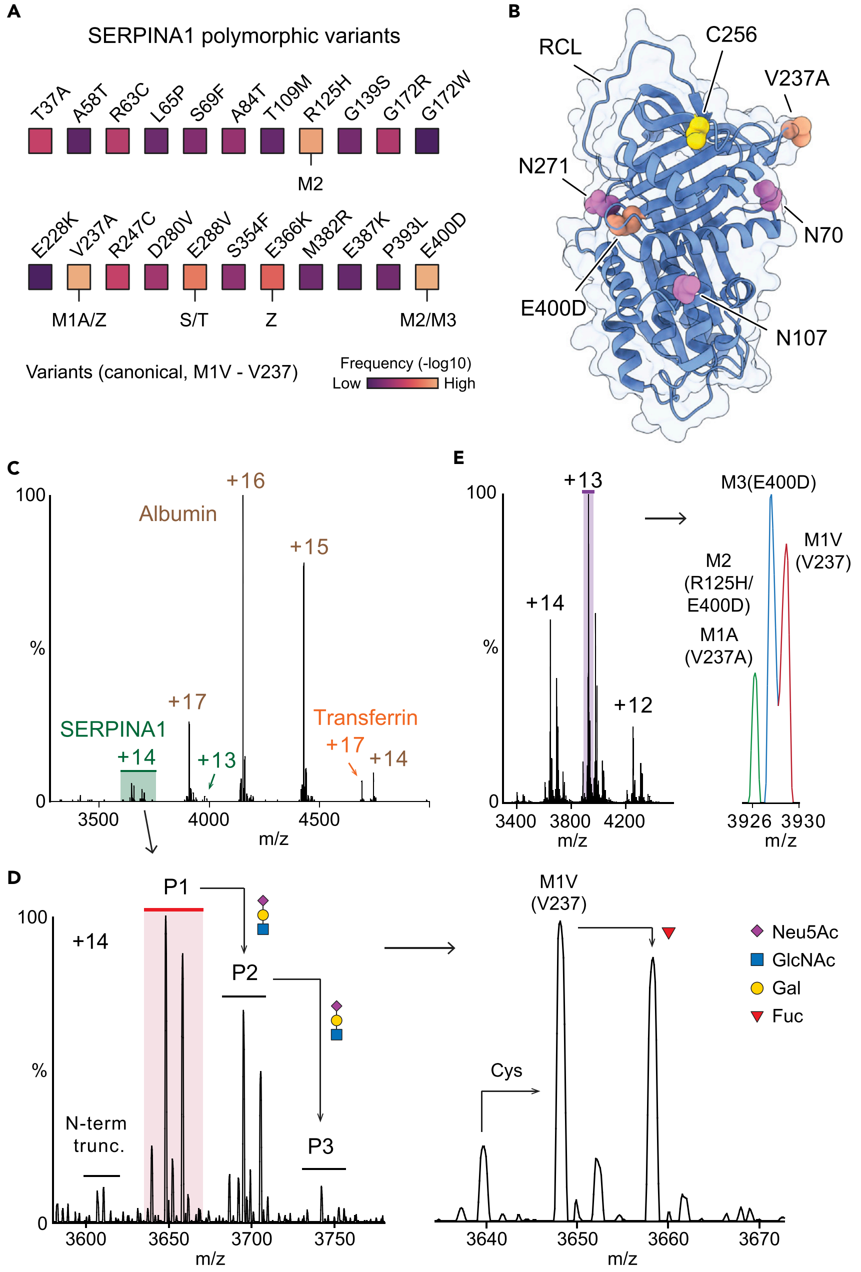

Figure 1. Probing SERPINA1 heterogeneity using native MS

(A) The frequency of common SERPINA1 polymorphisms. The M1A, M1V, M2, and M3 variants are the four most abundant isoforms encoded by the wild-type SERPINA1 sequence. S, T, and Z are pathogenic variants related to alpha-1 antitrypsin deficiency.

(B) SERPINA1 carries three N-glycosylation sites on Asn70, Asn107, and Asn271 (highlighted in pink) and one cysteinylation site on C256 (highlighted in yellow). Two mutations (Val237Ala and Glu400Asp) are highlighted in orange.

(C) Native MS of an undepleted human plasma sample from an individual donor. Albumin, SERPINA1, and transferrin peaks are labeled with the corresponding charge states. The albumin peaks with charge state +17 overlap with the SERPINA1 peaks with charge states +13.

(D) SERPINA1 peaks with charge state +14. Three main peak series, namely P1, P2, and P3 are observed that correspond to additions of Neu5Ac-Gal-GlcNAc units. Within those series, further heterogeneity is assigned to cysteinylation (Cys) and fucosylation. An N-terminal truncated form (N-term trunc.) missing Asp25 to Gly29 was also detected.

Figure 1. *Continued*

(E) Native mass spectrum of SERPINA1 from pooled plasma with peaks assigned to single amino acid mutations. Three peaks are annotated to M2/M1A, M3, and M1V variants, respectively. The mass spectra in (C) and (D) were acquired with R values of 30,000 and R = 25,000 for (E).

dominated by serum albumin with represents >50% of the plasma protein content (Figure 1C). Its different charge state peaks overlap those of other proteins, leading to ambiguous mass assignments for other plasma proteins. Therefore, we applied high-resolution conditions on the Orbitrap Eclipse platform (resolving power of 500,000 at *m/z* 200) to assign SERPINA1 peaks using a single charge state (Figure S1). The spectrum revealed the SERPINA1 proteoforms at the intact protein level, including the genetic variant (M1V), N-terminal truncation status, glycosylation heterogeneity (N-glycan branching and fucosylation), as well as the stoichiometry of cysteinylation (Figure 1D), in agreement with recent MS studies of SERPINA1.[23] N-glycan branching events (addition of a trisaccharide: Neu5Ac-Gal-GlcNAc; Neu5Ac, *N*-acetylneuraminic acid; Gal, galactose; GlcNAc, *N*-acetylglucosamine) account for the major heterogeneity of the SERPINA1 peaks spanning the molecular weight range from 51 to 53 kD.

To eliminate the interference from serum albumin and transferrin, and fully resolve SERPINA1 and SERPINA1-elastase complexes, we then analyzed isolated SERPINA1 from pooled human plasma. The native mass spectrum of SERPINA1 from pooled human plasma shows similar PTM patterns to the non-purified SERPINA1 from the individual donor (cysteinylation, glycosylation, and N-terminal truncation) (Figure S2), suggesting no bias to specific proteoforms was introduced during purification. Importantly, the four most common variants, M2 (His125/Asp400), M1A (Ala237), M3 (Asp400), and M1V (canonical sequence) could be observed with isotopic resolution under high-resolution conditions on the Orbitrap Eclipse platform (Figures 1E and S3). We further confirmed the presence of these variants by native top-down MS analysis (Figure S4) and MS-based bottom-up proteomics (Figure S5). Importantly this confirmation, together with the relative abundance determined from native MS, enables us to compare the protease binding events of these variants simultaneously and directly.

Probing proteoform-specific regulation of SERPINA1-elastase complexes

SERPINA1 traps elastase by a two-step process (Figure 2A). First, it captures elastase and forms a reversible Michaelis-Menten complex, subsequently, it is proteolytically cleaved and undergoes a large conformational change to form a stable complex with elastase. Several missense mutations and glycoforms in SERPINA1 reduce this protease inhibitory activity by unknown mechanisms.[9,24,25] To explore how these distinct proteoforms regulate the SERPINA1-elastase interactions, we first analyzed SERPINA1 mixed with sub-stoichiometric quantities of elastase, using our modified Eclipse mass spectrometer. The resulting spectrum shows peaks assigned to both apo-SERPINA1 and the SERPINA1-elastase complex (Figure S6A). Unexpectedly, high resolution was maintained within these ~80 kDa SERPINA1-elastase complexes at *m/z* > 4,500 enabling us to distinguish the single amino acid difference between M1V (canonical sequence) and M3 (Glu400Asp). We compared the relative abundances of peaks assigned to the SERPINA1-elastase complex with two bi-antennary and one tri-antennary N-glycans with the corresponding peaks of apo-SERPINA1 (Figure 2B). Interestingly, mono-fucosylation stabilizes the M1V variant-elastase complex but has no impact on the M3 variant-elastase complex (Figure 2C). Therefore, we hypothesize that this single amino acid difference (Glu400Asp) may cause variant-specific regulation through fucosylation.

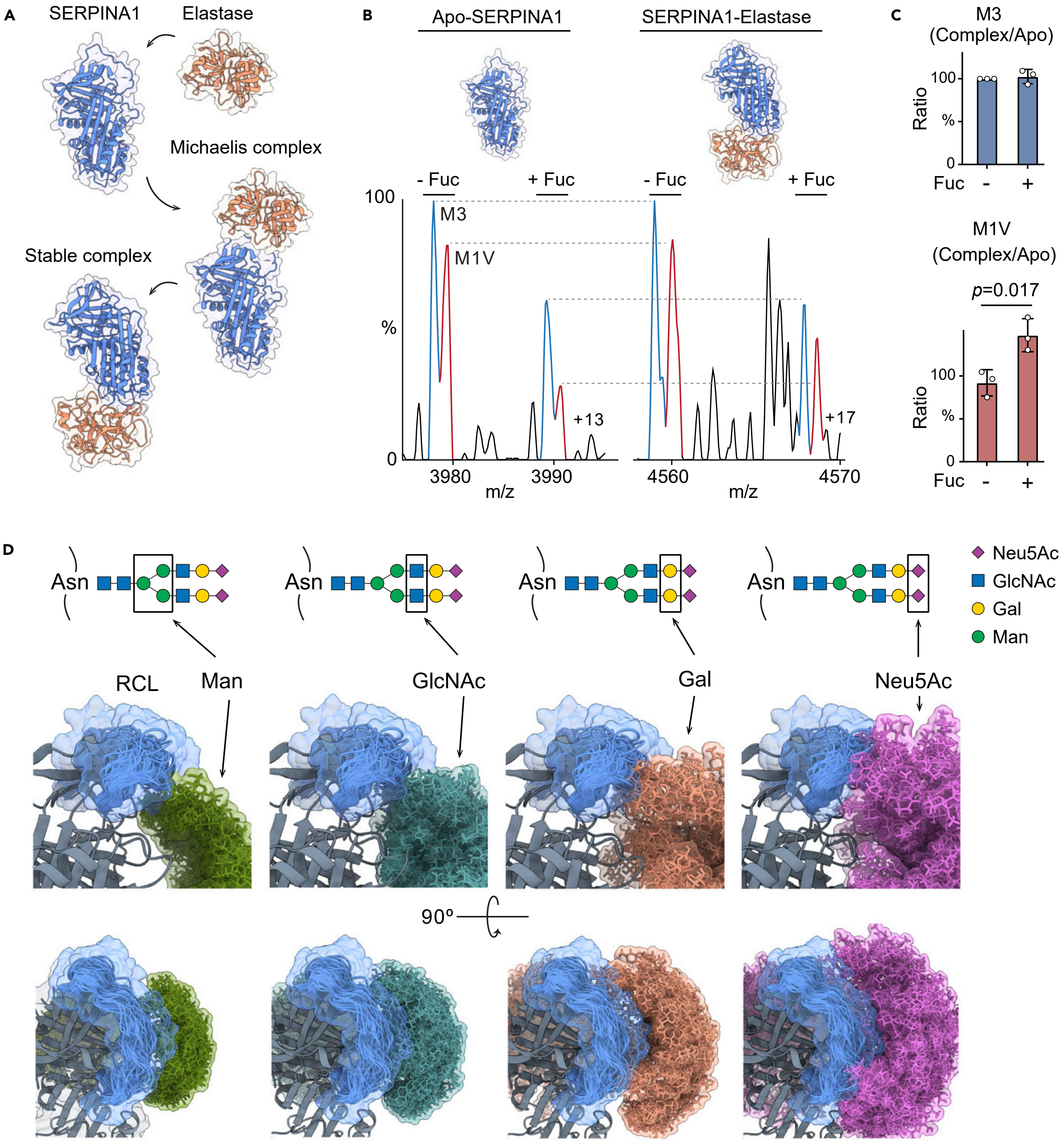

Figure 2. Analysis of SERPINA1-elastase complexes using native MS and MD simulations

(A) Illustrative mechanism of the SERPINA1-elastase interaction. Elastase recognizes the RCL and forms a Michaelis-Menten complex with SERPINA1. After hydrolysis of the enzymatic cleavage sites, elastase and SERPINA1 form a stable complex.

(B) Mass spectra of the SERPINA1 variants M3 and M1V in apo forms and in complex with elastase. The N-glycan composition and variants are labeled.

(C) Bar graphs of the ratio of the SERPINA1-elastase complex to apo-SERPINA1, with and without fucosylation. Bars show mean ± standard deviation with dots from three independent experiments. A Student's t test was performed to calculate the p value.

(D) Stereoview of the superimposed conformers of four different monosaccharide residues on Asn271 and the RCL from the 150 ns MD simulation trajectory. The snapshots of the monosaccharide residues and RCL conformations are extracted (one frame ns^{-1}) and overlayed.

To investigate the effects of this possible SNP regulation, we compared the structural differences between M1V and M3 variants (Figure S6B). The Glu400 in M1V (corresponding to Asp400 in M3) is proximal to the glycosylation site in the structure (Asn271) such that the side chain of Glu400 can form hydrogen bonds with the side chains of Thr126, Thr379, Ser381, and Asn402. The Glu400Asp mutation introduces a shorter negatively charged side chain to the M3 variant, which might affect its hydrogen bonding to other amino acids (Figures S6C and S6D). To investigate this, we performed molecular dynamics (MDs) simulations of the M1V and M3 variants. We found that while the Glu400Asp mutation does not impact protein backbone stability or conformation (Figures S6E and S6F), it does reduce hydrogen-bonding interactions to side chains (Figure S6G). As a consequence, the flexible side chains of Thr126, Thr379, and Asn402 might be anticipated to affect the N-glycan dynamics of the proximal Asn271.

We next investigated whether or not the N-glycans on SERPINA1 can influence the RCL, which is critical for activity (Figure 1B). We performed MD simulation of a fully glycosylated SERPINA1 (M1V variant) with three bi-antennary N-glycans with α2-6 sialylation on Asn70, Asn107, and Asn271 (Figures S7A and S7B). We then extracted snapshots of the N-glycans and RCL conformations over the simulation trajectory (one frame per 1 ns) and overlayed those conformations (Figure S7C). We found only the flexible N-glycan on Asn271 is proximal and able to interact with the RCL. To probe further the N-glycan (Asn271)-RCL interactions, we then extracted and overlayed snapshots of the individual monosaccharide residues (Figure 2D). Notably, RCL can contact the antennary monosaccharide residues (α2-6 Neu5Ac and β1-4 Gal) rather than the core α1-3,6 Man and β1-2 GlcNAc residues. Therefore, we propose that the terminal monosaccharide residues, namely α2-6 Neu5Ac and/or β1-4 Gal on the Asn271 N-glycan interact with the RCL and may regulate SERPINA1-elastase binding. We further released the negatively charged Neu5Ac residues from SERPINA1 using neuraminidase treatment to investigate whether we can suppress fucosylation regulation. We probed the desialylated SERPINA1-elastase complexes using native MS (Figure S8). Interestingly, we found the mono-fucosylation stabilization effect on M1V variant is completely abolished after the removal of the terminal Neu5Ac residues. Together, these results suggest that mono-fucosylation regulates M1V variant, and the terminal Neu5Ac residues are essential for this regulation.

Core fucosylation stabilizes SERPINA1 interactions with elastase

For SERPINA1 fucosylation, the fucose residue can be either α1-6 linked to the innermost GlcNAc residue (core fucosylation) or α1-3/4 linked to the branched GlcNAc residue (antennary fucosylation) (Figure 3A).[26] However, the core- and antennary fucosylated N-glycans are isomers that could not be distinguished by native MS. Therefore, we applied a double exoglycosidase treatment strategy to release Neu5Ac and Gal residues using neuraminidase and galactosidase (Figure 3A).[27] Briefly, either core- or antennary fucose residues do not have any impact on neuraminidase treatment. Nevertheless, antennary fucosylation sterically inhibits galactosidase activity and leaves one terminal galactose residue after digestion. This treatment transforms the fucosylated isomers into two smaller glycans with a difference of one galactose residue (162 Da). Following this treatment, we analyzed the double exoglycosidase digested SERPINA1 with native MS. The spectrum shows that all peaks of the trimmed SERPINA1 move to a lower *m/z* region compared with untreated SERPINA1 (Figure S9). After annotating all major peaks, we confirmed that all antennary Neu5Ac and Gal residues were removed from SERPINA1. Furthermore, the spectrum shows the M1V and M3 variants carry both

CellPress
OPEN ACCESS

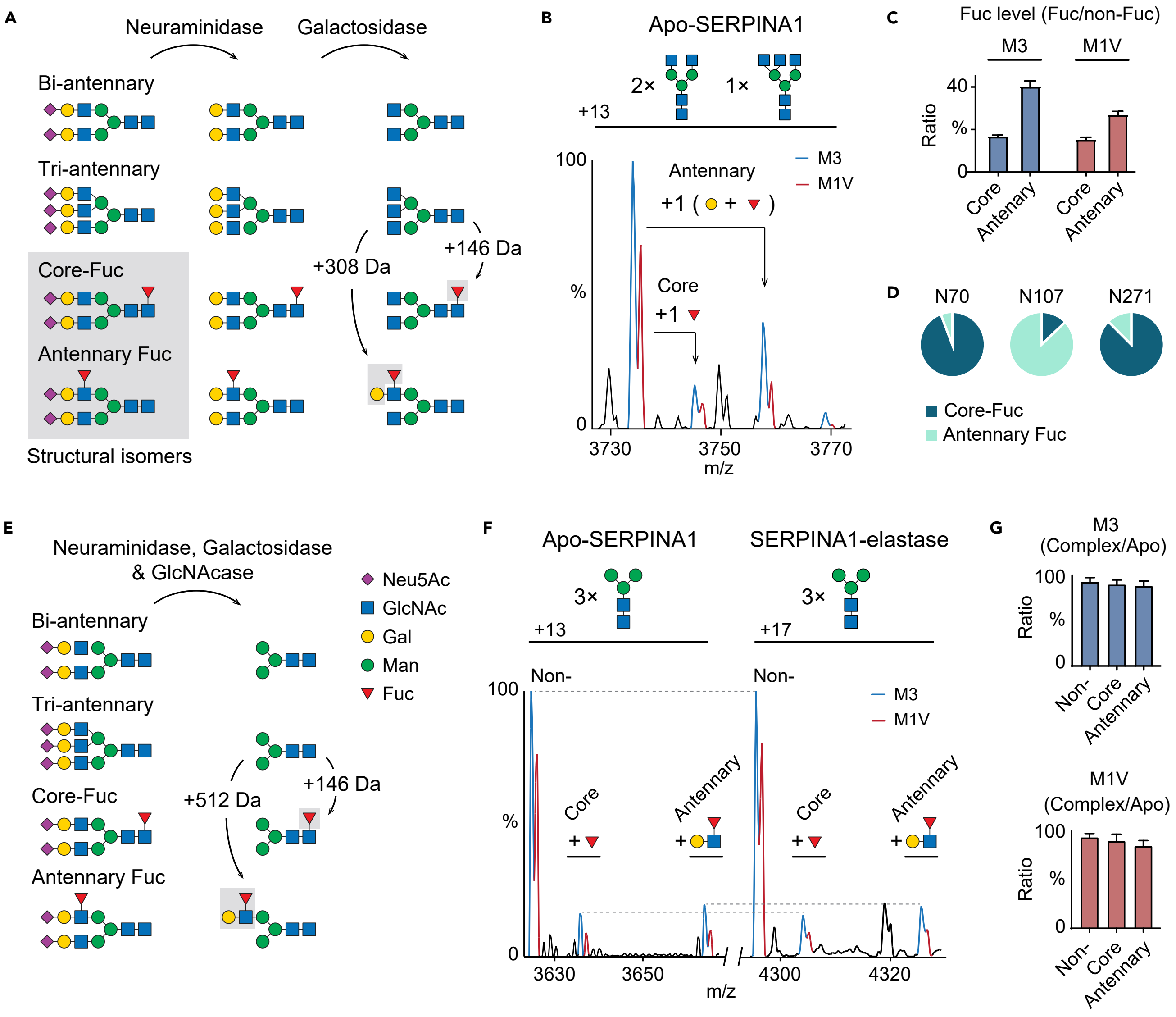

Figure 3. Analysis of core and antennary fucosylation on SERPINA1 and SERPINA1-elastase complexes

(A) Schematic illustration of neuraminidase and galactosidase digestion of N-glycans. Core-fucosylated and antennary fucosylated N-glycans are structural isomers. After double exoglycosidase digestion, the isomeric fucosylated N-glycans are transformed into two different structures with a mass difference of 162 Da (a galactose residue).

(B) Native mass spectrum of SERPINA1 digested with neuraminidase and galacosidase.

(C) Core- and antennary fucosylation levels of double exoglycosidase digested M3 and M1V variants. Bars show mean ± standard deviation.

(D) Quantification of site-specific core- and antennary fucosylation on Asn70, Asn107, and Asn271.

(E) Schematic illustration of triple exoglycosidase digestion for N-glycans using neuraminidase, galactosidase and GlcNAcase. The triple exoglycosidase digestion removes all non-fucosylated N-glycan antennae.

(F) Native mass spectra of triple exoglycosidase treated apo-SERPINA1 and SERPINA1-elastase complexes.

(G) Bar graphs of the ratios of the triple exoglycosidase treated SERPINA1 (M3 and M1V variants)-elastase complex to apo-SERPINA1. The ratios for non-, core- and antennary fucosylated forms are plotted. Bars show mean ± standard deviation.

core- and antennary fucose residues (Figures 3B and 3C). The ratio of the mass spectral peaks shows that the both variants bear similar core-fucosylation levels.

Next, we performed glycoproteomics of SERPINA1 to examine the site-specific fucosylation level (Figure S10). All three N-glycosylation sites (Asn70, Asn107, and Asn271) can be either core- or antennary fucosylated (Figure 3D). Importantly, the

fucosylation on Asn271 is dominated by core-fucosylated glycan (90%) with only 10% antennary fucosylation. Moreover, the antennary fucosylation is primarily present on Asn107, distal from RCL. A previous study revealed antennary and core fucosylation have different impacts on N-glycan conformation.[28] Hence, we hypothesize that the core-fucosylation level on the M1V variant may stabilize the M1V-elastase complexes via changing the N-glycan conformations and/or dynamics on Asn271.

To test this hypothesis, we first attempted to release the core-fucose residue on SERPINA1 with α1-6 fucosidase digestion. No de-fucosylated species was observed since core-fucose residues on intact glycoproteins are typically shielded by the extended sugar chain. This steric hindrance largely reduces the α1-6 fucosidase activity to folded glycoproteins.[9] Additionally, we found that the double exoglycosidase treatment of SERPINA1 generates an interfering GlcNAc adduct peak series, which overlaps with the fucosylated species in native MS analysis (Figure S11). To this end, we performed a triple exoglycosidase treatment to remove Neu5Ac, β1-4 Gal, and β1-2 GlcNAc residues using neuraminidase, galactosidase, and *N*-acetylglucosaminidase (GlcNAcase). This treatment completely removes antennary sugar residues on non-fucosylated glycans, leaving the trimannosyl pentasaccharide cores ($Man_3GlcNAc_2$) on SERPINA1 (Figure 3E). The core and antennary fucosylated glycans are transformed to the cores ($Man_3GlcNAc_2$) with a single core-fucose residue and a Galβ1-4(Fucα1-3/4)GlcNAc trisaccharide, respectively. We then analyzed the treated SERPINA1 with elastase to examine the extent of complex formation (Figure 3F). We found that there was no difference between the ratio of complexes for the two SNPs with core or antennary fucosylation. This allows us to conclude that core-fucose regulation is completely abolished when the outer arm residues (Neu5Ac-Gal-GlcNAc unit) are removed (Figure 3G). Moreover, the antennary fucosylation (Galβ1-4(Fucα1-3/4)GlcNAc unit), which primarily occurs on Asn107 does not play any function on SERPINA1-elastase interactions.

We further investigated whether N-glycan branching, in terms of additional Neu5Ac-Gal-GlcNAc units can modulate SERPINA1-elastase interactions. We compared the relative abundances of the SERPINA1-elastase complex with two bi- and one tri- antennary N-glycans with the complexes with three bi- antennary N-glycans (Figure S12A). However, we found that the additional Neu5Ac-Gal-GlcNAc unit on SERPINA1 does not significantly impact SERPINA1-elastase interactions (Figure S12B). We then analyzed the distribution of bi- and tri-antennary N-glycans across the three N-glycosylation sites in SERPINA1. We found that tri-antennary N-glycan is principally distributed on Asn107 (Figure S12C). Therefore, the additional Neu5Ac-Gal-GlcNAc unit on Asn107, which is distal to the RCL, does not have any significant effect on SERPINA1 interactions.

Collectively, we have shown that the N-glycan on Asn271 is critical for regulating SERPINA1-elastase binding via the antennary sugar moieties whereas additional Neu5Ac-Gal-GlcNAc units have little impact. Moreover, we attributed the stabilization effect on the M1V variant-elastase complex to core fucosylation on Asn271. However, the antennary fucosylation level on SERPINA1 Asn271 is relatively low. We, therefore selected another SERPIN, SERPINA3, which has higher levels of antennary fucosylation than SERPINA1, to understand further the regulation of SERPIN-protease interactions.

Core and antennary fucosylation play different roles in SERPINA3 interactions

SERPINA3 is structurally homologous to SERPINA1, but more highly glycosylated with six potential N-glycosylation sites (Asn93, Asn106, Asn127, Asn133, Asn186,

and Asn271) (Figures 4A and S13A). In contrast to SERPINA1, SERPINA3 carries multiple fucose residues.[29] This provides an opportunity to examine stoichiometric regulation via fucosylation. Similar to SERPINA1, SERPINA3 carries a complex type of N-glycan on Asn271, which is proximal to the RCL, such that it may regulate interactions with proteases (Figure S13B). To investigate this, we first analyzed desialylated SERPINA3 from human plasma and observed N-terminal truncated proteoforms, with highly branched N-glycans and fucosylation, in line with a previous native MS study (Figures S14A and S14B).[29] We also found a minor peak series corresponding to the full-length proteoform and further confirmed this observation by MS-based proteomics (Figures S14B–S14D).[30]

SERPINA3 is the endogenous inhibitor for cathepsin G secreted by neutrophils, chymase derived from mast cells and the angiotensin-converting enzyme.[31] Since both human chymase and cathepsin G are glycosylated, native MS of SERPINA3-protease complexes would be overly complicated. We therefore selected bovine chymotrypsin, a non-glycosylated structural homolog of human chymase and cathepsin G (Figure S15) to probed SERPINA3-chymotrypsin complexes by native MS. We quantified proteoforms of both unliganded apo forms and the complexes (Figures 4B, S16A, and S16B). Interestingly, we found that mono-fucosylation stabilizes the complex while further additions of fucose residues to the mono-fucosylated form, attenuate the interaction (Figures 4C, S16C, and S16D). Since fucose residues on SERPINA3 can be either core- or antennary linked, we hypothesized that core- and antennary fucosylation may have opposite effects on SERPINA3-protease interactions.

To test the hypothesis, we applied the triple exoglycosidase digestion strategy outlined above to transform the fucosylated isomers into two distinct glycans with a mass difference of 366 Da (Figure S16E). Native MS analysis of the treated SERPINA3-chymotrypsin mixture revealed SERPINA3 primarily carries one core fucosylation and up to six antennary fucose residues (Figures 4D and S16F). We then compared the ratios of complex to apo-form SERPINA1 with different antennary fucosylation statuses. We found the ratios are inversely proportional to the number of antennary fucose residues (Figure 4E), implying that antennary fucosylation stoichiometrically impairs SERPINA3-chymotrypsin interactions. We further examined the fucosylation status of Asn271 by glycoproteomics and confirmed the existence of multi-antennary fucosylation at this site (Figure S17). Given that antennary fucosylation attenuates SERPINA3-chymotrypsin binding, we attributed the stabilization effect of mono-fucosylation to core-fucose residues on SERPINA3. Since the N-glycan on Asn271 is proximal to the RCL, we ascribe the stoichiometric regulation of fucosylation to the multiple fucose residues on Asn271.

In summary, these results suggest that antennary fucosylation (Galβ1-4(Fucα1-3/4) GlcNAc trisaccharide) attenuates SERPINA3-chymotrypsin binding while core fucosylation (Fucα1-6) enhances its interactions. As the N-glycan antenna (Galβ1-4GlcNAc disaccharide) is essential for core-fucose stabilization, we interrogated whether N-glycan branching, in terms of Galβ1-4GlcNAc disaccharide, regulates SERPINA3-chymotrypsin interactions (Figure 4F). We further probed the N-glycan branching events on desialylated apo-SERPINA3 and SERPINA3-chymotrypsin complexes (Figures 4G and S18). Comparing the ratios of complex to apo forms of SERPINA3 with different N-glycan branching levels (number of additional Gal-GlcNAc units) (Figure 4H), we find that the ratio of complex to apo SERPINA3 is proportional to the number of additional Gal-GlcNAc residues on SEPINA3. These

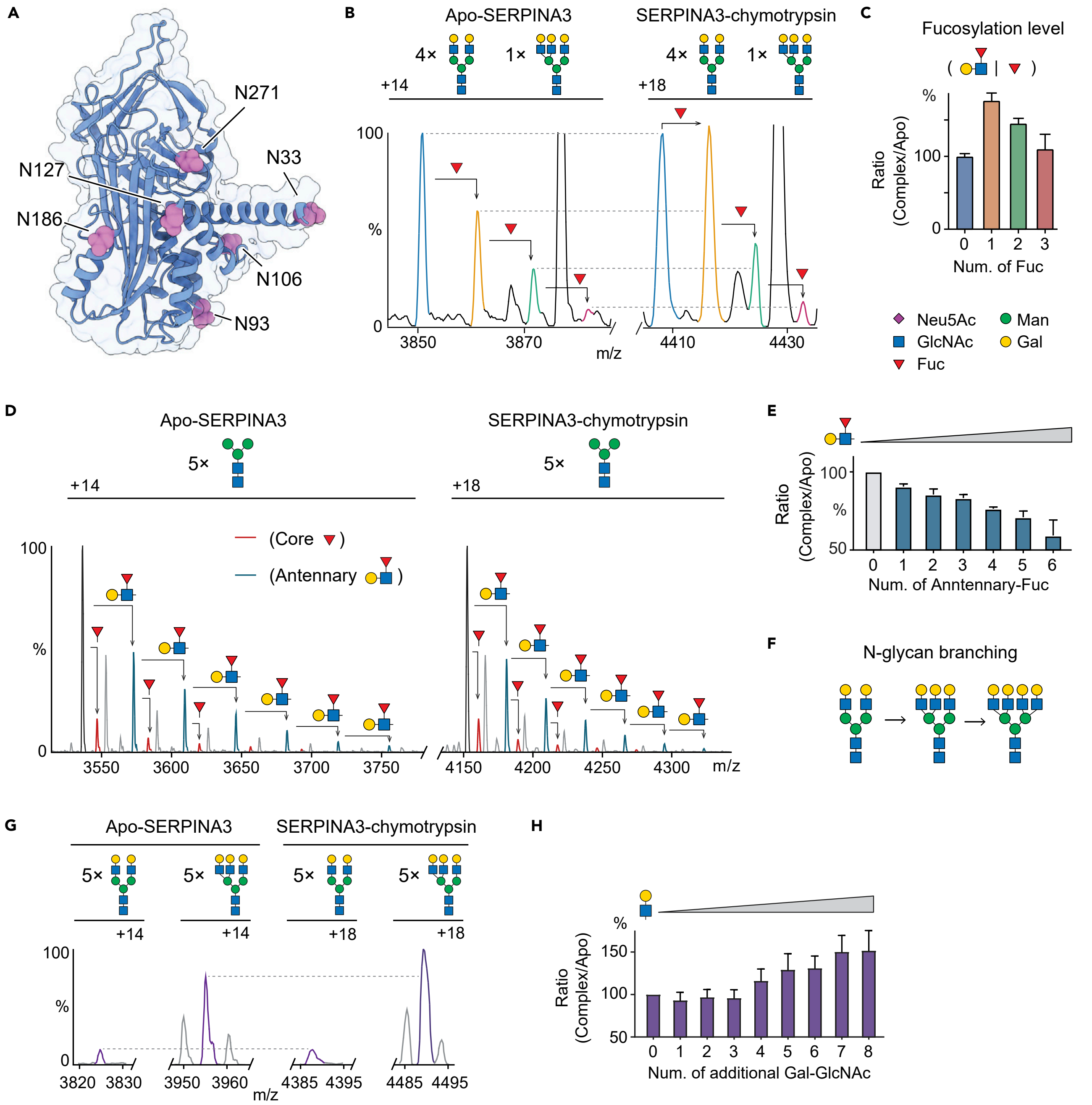

Figure 4. Probing N-glycan heterogeneity on SERPINA3-chymotrypsin complexes using native MS analysis

(A) Structure of SERPINA3. Six potential N-glycosylation sites, including Asn33, Asn93, Asn106, Asn127, Asn186, and Asn271 are highlighted in pink.

(B and C) (B) Native MS analysis of the desialylated SERPINA3-chymotrypsin complexes. Native mass spectra show the fucosylation status (0 to 3 fucose residues) of apo-form SERPINA3 and SERPINA3-chymotrypsin complexes. The ratios of their relative abundances are plotted as a bar graph in (C). Error bars represent the standard deviation of three individual replicates.

(D) Native MS analysis of triple exoglycosidase treated SERPINA3-chymotrypsin complexes reveals the stoichiometry and relative abundances of core- and antennary fucosylation on apo-SERPINA3 and SERPINA3-chymotrypsin complexes.

(E) The ratio shown in a bar graph of the triple exoglycosidase treated SERPINA3-chymotrypsin complex to apo-SERPINA3 with different numbers of antennary fucose residues. Bars show mean ± standard deviation from three independent experiments.

(F) Illustration of N-glycan branching events on SERPINA3.

(G and H) (G) Native MS analysis shows the N-glycan branching status on apo-SERPINA3 and SERPINA3-chymotrypsin complexes. The ratios of the relative abundances for the corresponding proteoforms (same numbers of additional Gal-GlcNAc units) are plotted as a bar graph in (H). Bars show mean ± standard deviation from three independent experiments.

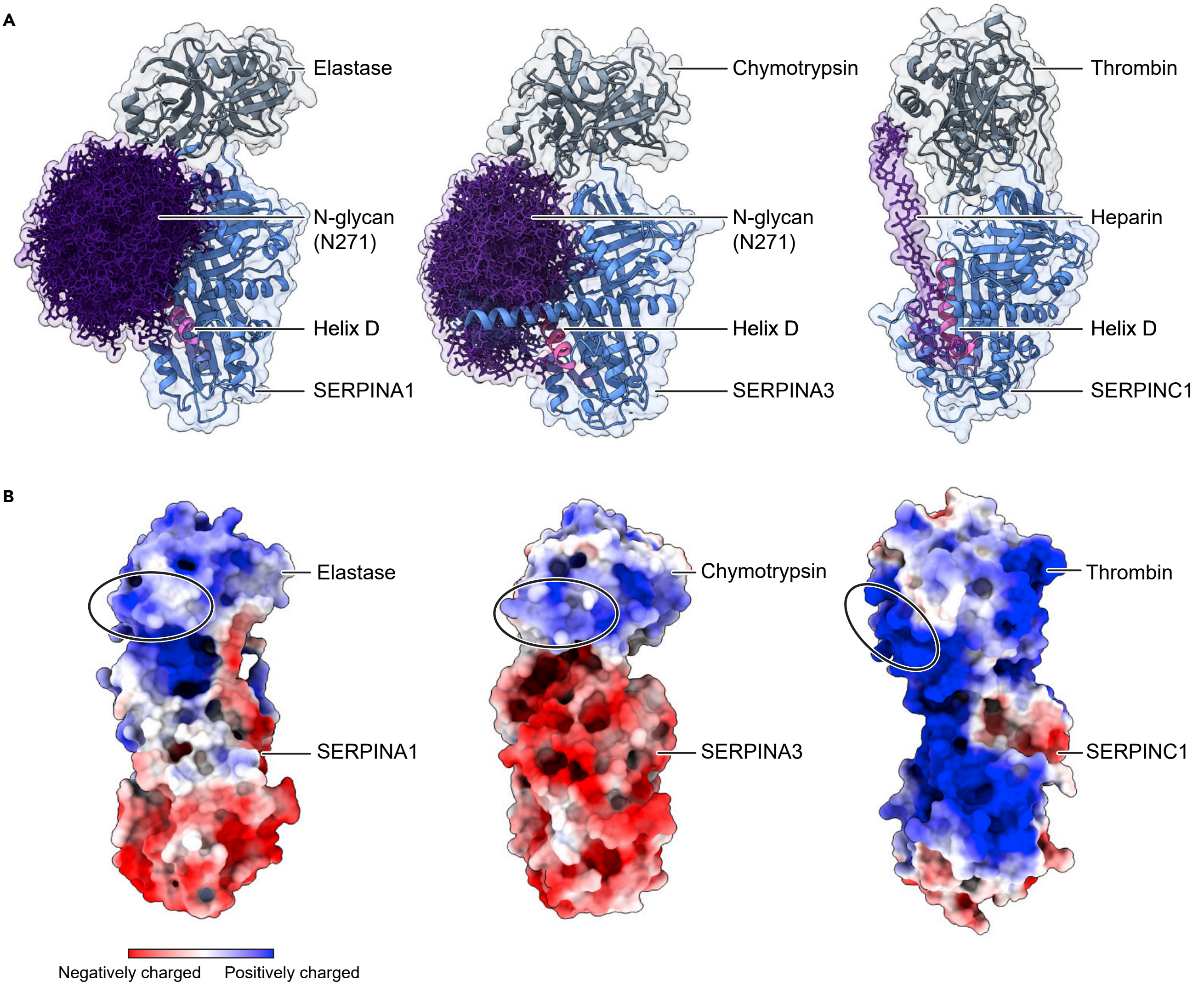

Figure 5. Structural analysis of SERPIN-protease complexes

(A) Structural comparison of SERPINA1-elastase, SERPINA3-chymotrypsin, and SERPINC1-thrombin-heparin complexes (PDB:1TB6). N-glycan on Asn271 is modeled on SERPINA1/A3. The positively charged helix D in SERPINA1/A3 and SERPINC1 are highlighted in pink.

(B) The surface electrostatic potential of SERPINA1-elastase, SERPINA3-chymotrypsin, and SERPINC1-thrombin complexes. The loop 3 and 6 of proteases at the interfacial domain are highlighted in black circles.

results imply that N-glycan branching stabilizes SERPINA3-chymotrypsin interactions in a stoichiometric manner (Figure 4H).

Implications for oligosaccharide mediated SERPIN interactions

Since the bulky N-glycan on Asn271 of SERPINA1/A3 regulates interactions via contacts with the RCL, we simulated conformations of the sialylated bi-antennary N-glycan on Asn271 in the SERPINA1/A3-protease complexes (Figures 5 and S19). We noted that the glycan on Asn271 shields helix D and the serine protease. Helix D is a well-defined oligosaccharide binding domain in several SERPINs, including SERPINC1 (Antithrombin), SERPIND1 (Heparin cofactor 2), and SERPINE2 (Glia-derived nexin) (Figure S20).[32] Therefore, we examined the crystal structure of the SERPINC1-thrombin-heparin complex to inform the oligosaccharide regulation of the SERPINA1/A3-protease interactions (Figure 5).[33] SERPINC1 is an endogenous

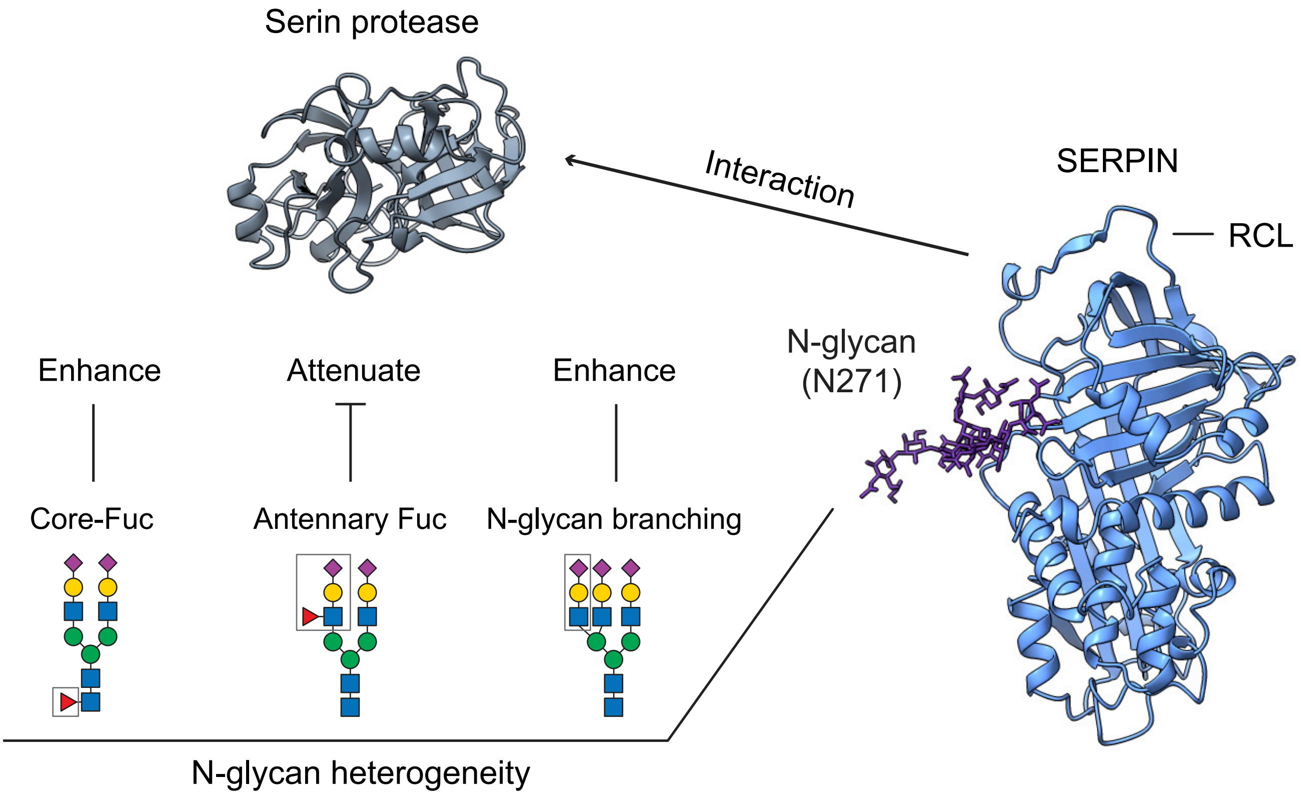

Figure 6. A general model for N-glycan regulation of SERPIN-serine protease interactions

The N-glycan on Asn271 plays a regulatory role for SERPIN-protease interactions. Core fucosylation and N-glycan branching enhance SERPIN-protease complex formation while antennary fucosylation attenuates the interactions.

inhibitor of thrombin, involved in the coagulation cascade, its positively charged helix D is available for oligosaccharide binding, since the corresponding Asn271 glycan is absent. Negatively charged heparin is known to bind tightly to helix D to regulate SERPINC1-thrombin interactions via two mechanisms.[33] The binding of heparin to SERPINC1 rearranges the RCL conformation and exposes the reactive sites. Alternatively, heparin bridges the protease and inhibitor to stabilize the non-covalently linked complex before enzymatic reaction. Therefore, it follows that the Asn271 glycan on SERPINA1/A3 may regulate the protease inhibitor interactions via similar mechanism as heparin to SERPINC1.

DISCUSSION

Depiction of proteoform-specific interactions is crucial to understanding how diverse proteoforms regulate protein functions. Here, we developed a native MS protocol to monitor individual SNPs on SERPIN-protease complexes and to relate this to site-specific glycan heterogeneity in SERPIN-protease interactions. We introduced sequential exoglycosidase treatments to differentiate core and antennary fucosylated structural isomers in glycoprotein complexes and performed MD simulation to distinguish site-specific glycan shielding effects on the reactive domain. We revealed that the N-glycan on Asn271 modulates SERPINA1/A3-protease interactions, and the position of the fucose residue (core and antennary) and N-glycan branching determines the regulatory role for SERPINA1/A3 interactions (Figure 6). Importantly, this glycosylation site (Asn271) is conserved in seven members of the SERPIN clade A, including six inhibitory proteins, namely SERPINA1, A2, A3, A5,

A10, and A12, and one non-inhibitory protein, SERPINA6 (Figure S21A). For SERPINA2, A5, A10, and A12, the N-glycan on this site is also close to the RCL (Figure S21B). Therefore, we propose that this N-glycosylation may play a regulatory role for all four inhibitory SERPINs. Indeed, previous reports described the corresponding N-glycosylation sites, namely Asn243 in SERPINA5 and Asn267 in SERPINA12, as important for protease inhibitory activity.[34,35] The non-inhibitory member, SERPINA6 is a corticosteroid-binding globulin that transports glucocorticoids and progestins in human blood.[13] Notably, the corresponding glycosylation site Asn260 is proximal to the corticosteroid-binding site and regulates corticosteroid-binding, while the conserved Glu389 in SERPINA6 (Glu400 in SERPINA1) is essential for corticosteroid-binding (Figure S21C).[36]

In this study, exoglycosidase treatment is key to reducing the complexity of native mass spectra for proteoform assignment and to distinguishing fucosylated isomers. We removed all Neu5Ac residues on SERPINA3 to avoid ambiguous assignment of two fucose residues (292.29 Da) and one Neu5Ac residue (291.26 Da). To explore the role of Neu5Ac residues, we modeled fully glycosylated apo-SERPNA3 and SERPINA3-chymotrypsin complexes with and without Neu5Ac residues (Figure S22). We found that some N-glycan conformers on Asn271 in SERPINA3 are proximal to chymotrypsin. Therefore, sialylation may affect fucosylation regulation of SERPINA3-serine protease interactions. Interestingly, several serine proteases, namely TMPRSS2, cathepsin G and chymase have also been reported to be modified with N-glycans. Therefore, we modeled fully glycosylated SERPINA1-TMPRSS2, SERPINA3-cathepsin G, and SERPINA3-chymase complexes to investigate how glycans on these serine proteases may impact SERPIN-protease interactions (Figure S23). We found that the glycans on TMPRSS2 and cathepsin G are distal to SERPINs. Interestingly, the N-glycan on Asn103 in chymase clashes with Asn106 N-glycan in SERPINA3 (Figure S23C). This steric constraint between N-glycans may be also involved in SERPINA3-chymase interactions.

Of recent interest, genomic and glycoproteomic studies of recovered COVID-19 patients revealed that fucosyltransferases and plasma glycoprotein fucosylation levels are associated with critical illness from COVID-19.[37–39] Based on our results, we speculate that additional fucose residues on SERPINA1 may regulate SERPINA1-TMPRSS2 interactions. As a consequence, these additional residues may therefore affect TMPRSS2-mediated SARS-CoV-2 spike protein priming and viral infection.[40] Interestingly, we found that regulation of the SERPINA1-serine protease interaction by fucosylation could be affected by a single amino acid variant (E400D) in SERPINA1, which is proximal to the N-glycan on Asn271 (Figure S6B). This finding implies that the variants, namely E400 or D400, may influence the severity of COVID-19. To our knowledge, there is no report of proteoform profiling of SERIPNA1 variants in COVID-19. We performed data analysis of a well-established proteomics dataset from COVID-19 patients to examine the expression levels of the E400 and D400 variants of SERPINA1.[41] We found significantly higher expression levels of SERPINA1$^{D400/D400}$, but not SERPINA1$^{E400/E400}$ in patients with severe symptoms (Figure S24). While it is tempting to speculate that the D400 mutation would affect hydrogen-bonding interactions with the N-glycan on Asn271, thereby impacting the RCL and its interactions with TMPRSS2, further details about the synergic effects of glycosylation and SNPs on SERPINA1 functions will be required to make definite conclusions.

In this study, we probed the circulating plasma SERPINs that are primarily secreted by hepatocytes. Interestingly, alveolar macrophages and monocytes

that are responsible for local SERPIN-protease balance in lung also secret SERPINs.[42] Probing the proteoforms of SERPINs from alveolar immune cells will inform how inflammatory stress reshapes the SERPIN proteoforms in lung diseases and affects local SERPIN-protease balance. Augmentation therapy using supplementary SERPINA1 to elevate circulating levels in patients is the only approved therapy for AATD-related lung diseases, including chronic obstructive pulmonary diseases and emphysema.[43] Moreover, recent clinical studies suggested that SERPINA1 is also a promising anti-inflammatory therapeutic to treat obesity-associated insulin resistance, cystic fibrosis, and COVID-19.[44,45] To date, all FDA-approved SERPINA1 products are directly purified from the pooled plasma of healthy donors. Previous studies of therapeutic recombinant SERPINA1 focused on protein engineering of the protein backbone to stabilize the protein structure and improve the circulation half-life.[46] Our results elucidate the regulation of N-glycan on Asn271 to SERPINA1-protease interactions and highlight an opportunity to design glycoengineered recombinant SERPINA1 to enhance protease inhibition activity.

Structural biology tools, namely cryoelectron microscopy and X-ray crystallography provide the structural basis to link disease-related mutations to protein structure and interactions. However, these structural biology approaches are limited in elucidating proteoforms of endogenous protein complexes with compositional and structural heterogeneities, especially the flexible glycosylation. Therefore, a combination of structural and MS-based approaches is essential to map proteoform information to structural models.[47–49] Importantly, the native MS approach provides a unique quantitative readout of the repertoire of proteoforms on protein complexes and connects specific proteoforms to protein interactions. Here, we elucidated site-specific proteoform regulation of SERPIN-protease complexes by combining native MS platform and computational simulations. Our approach has widespread applicability providing opportunities to characterize not only plasma proteins but also endogenous membrane receptor/transporter complexes to thereby inform the interactions and glycosylation status of disease-related proteoforms.

EXPERIMENTAL PROCEDURES

Resource availability

Lead contact

Further information and requests for resources and reagents should be directed to and will be fulfilled by the lead contact, Professor Carol V. Robinson (carol.robinson@chem.ox.ac.uk).

Material availability

This study did not generate new unique reagents.

Data and code availability

The raw MS data have been deposited at Figshare under the (https://doi.org/10.6084/m9.figshare.21206150) and are publicly available as of the date of publication.

Detailed experimental procedures can be found in the supplemental information.

Materials

Human plasma was from a healthy female donor (Cambridge Biosciences). Human SERPINA1 affinity-purified from pooled plasma was purchased from Sigma-Aldrich (Steinheim, Germany). Human SERPINA3, human elastase, formic acid, ammonium

acetate (7.5 M solution), and ammonium bicarbonate were purchased from Sigma-Aldrich (Steinheim, Germany). Liquid chromatography (LC) grade water, acetonitrile, and Tris were purchased from Merck. MS grade trypsin and chymotrypsin were purchased from Promega (Madison, WI, USA). Neuraminidase (α2-3,6,8,9), galactosidase (β1-4), and β-*N*-acetylglucosaminidase were from New England Biolabs.

Sample preparation

SERPINs were buffer-exchanged to 100 mM sodium citrate (pH 5.5), and incubated with glycosidase (glycoprotein:glycosidase = 50 μg:1 unit) at 37°C overnight for glycosidase digestion.[27] All SERPINs and proteases were extensively desalted with 1 M ammonium acetate using Amicon centrifugal filters (MWCO 10 K, Millipore) and then diluted into 200 mM ammonium acetate for native MS analysis.

Native MS analysis

SERPINs and proteases were stored on ice, mixed, and loaded into gold-coated needles prepared in-house and analyzed by native MS on a modified Orbitrap Eclipse Tribrid mass spectrometer in intact protein detection mode (Thermo Fisher Scientific).[22] Briefly, ion transmission was tuned for high *m/z* ions, higher-energy collisional dissociation (HCD) collision energies were optimized in the ion routing multipole for higher charge state species and the ion trap was optimized to trap, isolate and activate high *m/z* ions (up to 8,000 *m/z*). Typical MS settings were spray voltage 1.2 kV, and source temperature 150°C. The source fragmentation voltage was kept at 0–50 V to minimize in-source dissociation of glycoprotein complexes.[50]

Data processing

Native MS raw data processing and visualization were performed with Xcalibur 4.1 (Thermo Fisher Scientific). Protein and glycan mass calculations were based on amino acid and monosaccharide average residue masses. Glycoproteomics data were processed with Xcalibur 4.1 and PGlyco (version 2.0).[51] Glycoprotein structures were retrieved from PDB database and modeled with the Solution Builder module of CHARM-GUI[52] and GlycoSHIELD (version 0.1).[53] MDs simulation was performed with GROMACS 2020 with CUDA feature.[54] The glycoprotein molecular graphics and analysis were performed with UCSF ChimeraX (version 1.2.5)[55] and VMD (version 1.9.3).[56]

SUPPLEMENTAL INFORMATION

Supplemental information can be found online at https://doi.org/10.1016/j.chempr.2022.11.018.

ACKNOWLEDGMENTS

This research was funded in whole, or in part, by the Wellcome Trust grant no. 221795/Z/20/Z. For the purpose of Open Access, the author has applied a CC BY public copyright license to any Author Accepted Manuscript version arising from this submission.

AUTHOR CONTRIBUTIONS

D.W. and C.V.R. designed the research. D.W. performed MS experiments. D.W. and M.G. performed data analysis. D.W., M.G., and C.V.R. wrote the manuscript.

DECLARATION OF INTERESTS

The authors declare no competing interests.

Received: June 15, 2022
Revised: October 6, 2022
Accepted: November 22, 2022
Published: December 15, 2022

REFERENCES

1. Aebersold, R., Agar, J.N., Amster, I.J., Baker, M.S., Bertozzi, C.R., Boja, E.S., Costello, C.E., Cravatt, B.F., Fenselau, C., Garcia, B.A., et al. (2018). How many human proteoforms are there? Nat. Chem. Biol. *14*, 206–214. https://doi.org/10.1038/nchembio.2576.

2. Chen, L., Liu, S., and Tao, Y. (2020). Regulating tumor suppressor genes: post-translational modifications. Signal Transduct. Target. Ther. *5*, 90. https://doi.org/10.1038/s41392-020-0196-9.

3. Rodriguez, H., Zenklusen, J.C., Staudt, L.M., Doroshow, J.H., and Lowy, D.R. (2021). The next horizon in precision oncology: proteogenomics to inform cancer diagnosis and treatment. Cell *184*, 1661–1670. https://doi.org/10.1016/j.cell.2021.02.055.

4. Geyer, P.E., Holdt, L.M., Teupser, D., and Mann, M. (2017). Revisiting biomarker discovery by plasma proteomics. Mol. Syst. Biol. *13*, 942. https://doi.org/10.15252/msb.20156297.

5. Sun, B.B., Maranville, J.C., Peters, J.E., Stacey, D., Staley, J.R., Blackshaw, J., Burgess, S., Jiang, T., Paige, E., Surendran, P., et al. (2018). Genomic atlas of the human plasma proteome. Nature *558*, 73–79. https://doi.org/10.1038/s41586-018-0175-2.

6. Clerc, F., Reiding, K.R., Jansen, B.C., Kammeijer, G.S.M., Bondt, A., and Wuhrer, M. (2016). Human plasma protein N-glycosylation. Glycoconj. J. *33*, 309–343. https://doi.org/10.1007/s10719-015-9626-2.

7. Sanrattana, W., Maas, C., and de Maat, S. (2019). SERPINs—from trap to treatment. Front. Med. (Lausanne) *6*, 25. https://doi.org/10.3389/fmed.2019.00025.

8. Ahern, D.J., Ai, Z., Ainsworth, M., Allan, C., Allcock, A., Angus, B., Ansari, M.A., Arancibia-Cárcamo, C.V., Aschenbrenner, D., Attar, M., et al. (2022). A blood atlas of COVID-19 defines hallmarks of disease severity and specificity. Cell *185*. 916–938.e58. https://doi.org/10.1016/j.cell.2022.01.012.

9. McCarthy, C., Saldova, R., Wormald, M.R., Rudd, P.M., McElvaney, N.G., and Reeves, E.P. (2014). The role and importance of glycosylation of acute phase proteins with focus on Alpha-1 antitrypsin in acute and chronic inflammatory conditions. J. Proteome Res. *13*, 3131–3143. https://doi.org/10.1021/pr500146y.

10. Inouye, M., Ripatti, S., Kettunen, J., Lyytikäinen, L.-P., Oksala, N., Laurila, P.-P., Kangas, A.J., Soininen, P., Savolainen, M.J., Viikari, J., et al. (2012). Novel loci for metabolic networks and multi-tissue expression studies reveal genes for atherosclerosis. PLoS Genet. *8*, e1002907. https://doi.org/10.1371/journal.pgen.1002907.

11. Sun, Z., and Yang, P. (2004). Role of imbalance between neutrophil elastase and α1-antitrypsin in cancer development and progression. Lancet Oncol. *5*, 182–190. https://doi.org/10.1016/S1470-2045(04)01414-7.

12. Wettstein, L., Weil, T., Conzelmann, C., Müller, J.A., Groß, R., Hirschenberger, M., Seidel, A., Klute, S., Zech, F., Prelli Bozzo, C., et al. (2021). Alpha-1 antitrypsin inhibits TMPRSS2 protease activity and SARS-CoV-2 infection. Nat. Commun. *12*, 1726. https://doi.org/10.1038/s41467-021-21972-0.

13. Law, R.H.P., Zhang, Q., McGowan, S., Buckle, A.M., Silverman, G.A., Wong, W., Rosado, C.J., Langendorf, C.G., Pike, R.N., Bird, P.I., and Whisstock, J.C. (2006). An overview of the serpin superfamily. Genome Biol. *7*, 216. https://doi.org/10.1186/gb-2006-7-5-216.

14. Malik, R., Dau, T., Gonik, M., Sivakumar, A., Deredge, D.J., Edeleva, E.V., Götzfried, J., Van Der Laan, S.W., Pasterkamp, G., Beaufort, N., et al. (2017). Common coding variant in SERPINA1 increases the risk for large artery stroke. Proc. Natl. Acad. Sci. USA *114*, 3613–3618. https://doi.org/10.1073/pnas.1616301114.

15. Bolton, J.L., Hayward, C., Direk, N., Lewis, J.G., Hammond, G.L., Hill, L.A., Anderson, A., Huffman, J., Wilson, J.F., Campbell, H., et al. (2014). Genome wide association identifies common variants at the SERPINA6/SERPINA1 locus influencing plasma cortisol and corticosteroid binding globulin. PLoS Genet. *10*, e1004474. https://doi.org/10.1371/journal.pgen.1004474.

16. Varki, A. (2017). Biological roles of glycans. Glycobiology *27*, 3–49. https://doi.org/10.1093/glycob/cww086.

17. Lin, Y.H., Franc, V., and Heck, A.J.R. (2018). Similar albeit not the same: in-depth analysis of proteoforms of human serum, bovine serum, and recombinant human fetuin. J. Proteome Res. *17*, 2861–2869. https://doi.org/10.1021/acs.jproteome.8b00318.

18. Melani, R.D., Gerbasi, V.R., Anderson, L.C., Sikora, J.W., Toby, T.K., Hutton, J.E., Butcher, D.S., Negrão, F., Seckler, H.S., Srzentić, K., et al. (2022). The Blood Proteoform Atlas: a reference map of proteoforms in human hematopoietic cells. Science *375*, 411–418. https://doi.org/10.1126/science.aaz5284.

19. Smith, L.M., and Kelleher, N.L. (2018). Proteoforms as the next proteomics currency. Science *359*, 1106–1107. https://doi.org/10.1126/science.aat1884.

20. Bludau, I., and Aebersold, R. (2020). Proteomic and interactomic insights into the molecular basis of cell functional diversity. Nat. Rev. Mol. Cell Biol. *21*, 327–340. https://doi.org/10.1038/s41580-020-0231-2.

21. Wu, D., and Robinson, C.V. (2021). Connecting 'multi-omics' approaches to endogenous protein complexes. Trends Chem. *3*, 445–455. https://doi.org/10.1016/j.trechm.2021.03.007.

22. Gault, J., Liko, I., Landreh, M., Shutin, D., Bolla, J.R., Jefferies, D., Agasid, M., Yen, H.-Y., Ladds, M.J.G.W., Lane, D.P., et al. (2020). Combining native and 'omics' mass spectrometry to identify endogenous ligands bound to membrane proteins. Nat. Methods *17*, 505–508. https://doi.org/10.1038/s41592-020-0821-0.

23. Jager, S., Cramer, D.A.T., Hoek, M., Mokiem, N.J., van Keulen, B.J., van Goudoever, J.B., Dingess, K.A., and Heck, A.J.R. (2022). Proteoform profiles reveal that alpha-1-antitrypsin in human serum and milk is derived from a common source. Front. Mol. Biosci. *9*, 858856. https://doi.org/10.3389/fmolb.2022.858856.

24. McCarthy, C., Dunlea, D.M., Saldova, R., Henry, M., Meleady, P., McElvaney, O.J., Marsh, B., Rudd, P.M., Reeves, E.P., and McElvaney, N.G. (2018). Glycosylation repurposes alpha-1 antitrypsin for resolution of community-acquired pneumonia. Am. J. Respir. Crit. Care Med. *197*, 1346–1349. https://doi.org/10.1164/rccm.201709-1954LE.

25. Blanchard, V., Liu, X., Eigel, S., Kaup, M., Rieck, S., Janciauskiene, S., Sandig, V., Marx, U., Walden, P., Tauber, R., and Berger, M. (2011). N-glycosylation and biological activity of recombinant human alpha1-antitrypsin expressed in a novel human neuronal cell line. Biotechnol. Bioeng. *108*, 2118–2128. https://doi.org/10.1002/bit.23158.

26. Kolarich, D., Weber, A., Turecek, P.L., Schwarz, H.-P., and Altmann, F. (2006). Comprehensive glyco-proteomic analysis of human α1-antitrypsin and its charge isoforms. Proteomics *6*, 3369–3380. https://doi.org/10.1002/pmic.2.

27. Chen, S., Wu, D., Robinson, C.V., and Struwe, W.B. (2021). Native mass spectrometry meets glycomics: resolving structural detail and occupancy of glycans on intact glycoproteins. Anal. Chem. *93*, 10435–10443. https://doi.org/10.1021/acs.analchem.1c01460.

28. Nishima, W., Miyashita, N., Yamaguchi, Y., Sugita, Y., and Re, S. (2012). Effect of bisecting GlcNAc and core fucosylation on conformational properties of biantennary complex-type N-glycans in solution. J. Phys.

Chem. B *116*, 8504–8512. https://doi.org/10.1021/jp212550z.

29. Čaval, T., Lin, Y., Varkila, M., Reiding, K.R., Bonten, M.J.M., Cremer, O.L., Franc, V., and Heck, A.J.R. (2021). Glycoproteoform profiles of individual patients' plasma alpha-1-antichymotrypsin are unique and extensively remodeled following a septic episode. Front. Immunol. *11*, 608466. https://doi.org/10.3389/fimmu.2020.608466.

30. Lindmark, B., Lilja, H., Alm, R., and Eriksson, S. (1989). The microheterogeneity of desialylated α1-antichymotrypsin: the occurrence of two amino-terminal isoforms, one lacking a His-Pro dipeptide. Biochim. Biophys. Acta *997*, 90–95. https://doi.org/10.1016/0167-4838(89)90139-8.

31. Kalsheker, N.A. (1996). α1-antichymotrypsin. Int. J. Biochem. Cell Biol. *28*, 961–964. https://doi.org/10.1016/1357-2725(96)00032-5.

32. Bhakuni, T., Ali, M.F., Ahmad, I., Bano, S., Ansari, S., and Jairajpuri, M.A. (2016). Role of heparin and non heparin binding serpins in coagulation and angiogenesis: a complex interplay. Arch. Biochem. Biophys. *604*, 128–142. https://doi.org/10.1016/j.abb.2016.06.018.

33. Li, W., Johnson, D.J.D., Esmon, C.T., and Huntington, J.A. (2004). Structure of the antithrombin–thrombin–heparin ternary complex reveals the antithrombotic mechanism of heparin. Nat. Struct. Mol. Biol. *11*, 857–862. https://doi.org/10.1038/nsmb811.

34. Fujita, M., Izutani, W., Takahashi, K., Nishizawa, K., Shirono, H., and Koga, J. (2002). Role of each Asn-linked glycan in the anticoagulant activity of human protein C inhibitor. Thromb. Res. *105*, 95–102. https://doi.org/10.1016/S0049-3848(01)00398-X.

35. Oertwig, K., Ulbricht, D., Hanke, S., Pippel, J., Bellmann-Sickert, K., Sträter, N., and Heiker, J.T. (2017). Glycosylation of human vaspin (SERPINA12) and its impact on serpin activity, heparin binding and thermal stability. Biochim. Biophys. Acta Proteins Proteom. *1865*, 1188–1194. https://doi.org/10.1016/j.bbapap.2017.06.020.

36. Emptoz-Bonneton, A. (2000). Novel human corticosteroid-binding globulin variant with low cortisol-binding affinity. J. Clin. Endocrinol. Metab. *85*, 361–367. https://doi.org/10.1210/jc.85.1.361.

37. Kousathanas, A., Pairo-Castineira, E., Rawlik, K., Stuckey, A., Odhams, C.A., Walker, S., Russell, C.D., Malinauskas, T., Wu, Y., Millar, J., et al. (2022). Whole-genome sequencing reveals host factors underlying critical COVID-19. Nature *607*, 97–103. https://doi.org/10.1038/s41586-022-04576-6.

38. Byeon, S.K., Madugundu, A.K., Garapati, K., Ramarajan, M.G., Saraswat, M., Kumar, M.,P., Hughes, T., Shah, R., Patnaik, M.M., et al. (2022). Development of a multiomics model for identification of predictive biomarkers for COVID-19 severity: a retrospective cohort study. Lancet Digit Health *4*, e632–e645. https://doi.org/10.1016/S2589-7500(22)00112-1.

39. White, M.E.H., Jones, D.M., Folter, J. De, Aulakh, S.K., Helen, R., Krüger, L., Demichev, V., Tober-Lau, P., and Kurth, F. (2022). OxoScan-MS: oxonium ion scanning mass spectrometry facilitates plasma glycoproteomics in large scale. Preprint at bioRxiv. https://doi.org/10.1101/2022.06.01.494393.

40. Hoffmann, M., Kleine-Weber, H., Schroeder, S., Krüger, N., Herrler, T., Erichsen, S., Schiergens, T.S., Herrler, G., Wu, N.H., Nitsche, A., et al. (2020). SARS-CoV-2 cell entry depends on ACE2 and TMPRSS2 and is blocked by a clinically proven protease inhibitor. Cell *181*. 271–280.e8. https://doi.org/10.1016/j.cell.2020.02.052.

41. Shu, T., Ning, W., Wu, D., Xu, J., Han, Q., Huang, M., Zou, X., Yang, Q., Yuan, Y., Bie, Y., et al. (2020). Plasma proteomics identify biomarkers and pathogenesis of COVID-19. Immunity *53*. 1108–1122.e5. https://doi.org/10.1016/j.immuni.2020.10.008.

42. Belchamber, K.B.R., Walker, E.M., Stockley, R.A., and Sapey, E. (2020). Monocytes and macrophages in alpha-1 antitrypsin deficiency. Int. J. Chron. Obstruct. Pulmon. Dis. *15*, 3183–3192. https://doi.org/10.2147/COPD.S276792.

43. Sandhaus, R.A. (2004). alpha1-Antitrypsin deficiency. 6: new and emerging treatments for alpha1-antitrypsin deficiency. Thorax *59*, 904–909. https://doi.org/10.1136/thx.2003.006551.

44. Kim, M., Cai, Q., and Oh, Y. (2018). Therapeutic potential of alpha-1 antitrypsin in human disease. Ann. Pediatr. Endocrinol. Metab. *23*, 131–135. https://doi.org/10.6065/apem.2018.23.3.131.

45. McElvaney, O.J., McEvoy, N.L., Boland, F., McElvaney, O.F., Hogan, G., Donnelly, K., Friel, O., Browne, E., Fraughen, D.D., Murphy, M.P., et al. (2022). A randomized, double-blind, placebo-controlled trial of intravenous alpha-1 antitrypsin for ARDS secondary to COVID-19. Med (NY) *3*. 233–248.e6. https://doi.org/10.1016/j.medj.2022.03.001.

46. Maas, C., and de Maat, S. (2021). Therapeutic SERPINs: improving on nature. Front. Cardiovasc. Med. *8*, 648349. https://doi.org/10.3389/fcvm.2021.648349.

47. Arakhamia, T., Lee, C.E., Carlomagno, Y., Kumar, M., Duong, D.M., Wesseling, H., Kundinger, S.R., Wang, K., Williams, D., DeTure, M., et al. (2020). Posttranslational modifications mediate the structural diversity of tauopathy strains. Cell *180*. 633–644.e12. https://doi.org/10.1016/j.cell.2020.01.027.

48. Weiss, G.L., Stanisich, J.J., Sauer, M.M., Lin, C.W., Eras, J., Zyla, D.S., Trück, J., Devuyst, O., Aebi, M., Pilhofer, M., et al. (2020). Architecture and function of human uromodulin filaments in urinary tract infections. Science *369*, 1005–1010. https://doi.org/10.1126/science.aaz9866.

49. Yao, H., Song, Y., Chen, Y., Wu, N., Xu, J., Sun, C., Zhang, J., Weng, T., Zhang, Z., Wu, Z., et al. (2020). Molecular architecture of the SARS-CoV-2 virus. Cell *183*. 730–738.e13. https://doi.org/10.1016/j.cell.2020.09.018.

50. Wu, D., Struwe, W.B., Harvey, D.J., Ferguson, M.A.J., and Robinson, C.V. (2018). N-glycan microheterogeneity regulates interactions of plasma proteins. Proc. Natl. Acad. Sci. USA *115*, 8763–8768. https://doi.org/10.1073/pnas.1807439115.

51. Liu, M.-Q., Zeng, W.-F., Fang, P., Cao, W.-Q., Liu, C., Yan, G.-Q., Zhang, Y., Peng, C., Wu, J.-Q., Zhang, X.-J., et al. (2017). pGlyco 2.0 enables precision N-glycoproteomics with comprehensive quality control and one-step mass spectrometry for intact glycopeptide identification. Nat. Commun. *8*, 438. https://doi.org/10.1038/s41467-017-00535-2.

52. Park, S.J., Lee, J., Qi, Y., Kern, N.R., Lee, H.S., Jo, S., Joung, I., Joo, K., Lee, J., and Im, W. (2019). CHARMM-GUI Glycan Modeler for modeling and simulation of carbohydrates and glycoconjugates. Glycobiology *29*, 320–331. https://doi.org/10.1093/glycob/cwz003.

53. Gecht, M., von Bülow, S., Penet, C., Hummer, G., Hanus, C., and Sikora, M. (2021). GlycoSHIELD: a versatile pipeline to assess glycan impact on protein structures. Preprint at bioRxiv. https://doi.org/10.1101/2021.08.04.455134.

54. Berendsen, H.J.C., van der Spoel, D., and van Drunen, R. (1995). GROMACS: a message-passing parallel molecular dynamics implementation. Comput. Phys. Commun. *91*, 43–56. https://doi.org/10.1016/0010-4655(95)00042-E.

55. Pettersen, E.F., Goddard, T.D., Huang, C.C., Couch, G.S., Greenblatt, D.M., Meng, E.C., and Ferrin, T.E. (2004). UCSF Chimera—a visualization system for exploratory research and analysis. J. Comput. Chem. *25*, 1605–1612. https://doi.org/10.1002/jcc.20084.

56. Humphrey, W., Dalke, A., and Schulten, K. (1996). VMD: visual molecular dynamics. J. Mol. Graph. *14*, 33–38. https://doi.org/10.1016/0263-7855(96)00018-5.

Chem, Volume *9*

Supplemental information

Connecting single-nucleotide polymorphisms, glycosylation status, and interactions of plasma serine protease inhibitors

Di Wu, Manman Guo, and Carol V. Robinson

Table of contents

I. Supplemental experimental procedures …………………………………………… 1

Detailed Methods ……………………………………………………………… 1

Figure S1 ……………………………………………………………………… 5

Figure S2 ……………………………………………………………………… 6

Figure S3 ……………………………………………………………………… 7

Figure S4 ……………………………………………………………………… 8

Figure S5 ……………………………………………………………………… 9

Figure S6 ……………………………………………………………………… 10

Figure S7 ……………………………………………………………………… 11

Figure S8 ……………………………………………………………………… 12

Figure S9 ……………………………………………………………………… 13

Figure S10 …………………………………………………………………… 14

Figure S11 …………………………………………………………………… 15

Figure S12 …………………………………………………………………… 16

Figure S13 …………………………………………………………………… 17

Figure S14 …………………………………………………………………… 18

Figure S15 …………………………………………………………………… 19

Figure S16 …………………………………………………………………… 20

Figure S17 …………………………………………………………………… 21

Figure S18 …………………………………………………………………… 22

Figure S19 …………………………………………………………………… 23

Figure S20 …………………………………………………………………… 23

Figure S21 …………………………………………………………………… 24

Figure S22 …………………………………………………………………… 25

Figure S23 …………………………………………………………………… 26

Figure S24 …………………………………………………………………… 27

II. Supplemental references ……………………………………………………… 28

Supplemental experimental procedures

Detailed Methods

Sample preparation of human plasma. Fresh human plasma (reference number: RDN-6711552, Research Donors Ltd.) was separated from fresh whole blood from a female donor collected in a vacuette tube containing sodium citrate anticoagulant. This human plasma was then centrifuged at 12000g for 10 min and the supernatant diluted with 1 M ammonium acetate to a final protein concentration of 1 mg/ml and buffer-exchanged into 200 mM ammonium acetate (pH 7.0) using a 10 kD MWCO centrifugal filter (Amicon Ultra-0.5 ml, Millipore) prior to native MS analysis.

Preparation for native MS. The isolated SERPINA1, SERPINA3, elastase and chymotrypsin were reconstituted in 50 mM Tris, pH7.4 at a concentration of 1 mg/ml for further treatment. Glycoproteins were buffer-exchanged into 200 mM ammonium acetate (pH 7.0) using a 10 kD MWCO centrifugal filter (Amicon Ultra-0.5 ml, Millipore) prior to native MS analysis.

Exoglycosidase treatment. The exoglycosidase digestion protocol was adapted from Chen et al [1]. Briefly, 200 µg glycoprotein (1 mg/ml) was buffer-exchanged into 50 mM sodium citrate buffer (pH 5.5) using a 10 kD MWCO centrifugal filter (Amicon Ultra-0.5 ml, Millipore). The glycoproteins were incubated first desialylated with neuraminidase (α2-3,6,8,9) then further incubated with galactosidase (β1-4) to remove the Gal residues and then incubated β-*N*-acetylglucosaminidase to release the GlcNAc residues (all three reactions performed at 37 ºC overnight). The exoglycosidase-treated glycoproteins were buffer-exchanged to 200 mM ammonium acetate for MS analysis.

Resolving isotopic peaks of human plasma proteins using native MS. Plasma proteins and SERINA1 were analysed on a modified Orbitrap Eclipse platform. The human plasma proteins and SERPINA1 were extensively desalted before the analysis. For native MS analysis, the mass spectra were firstly acquired using a resolution of 17500 to ensure complete removal of sodium and potassium ions, and then acquired using a resolution of 500000 with the spectra averaging function for resolving isotopic peaks.

Native top-down MS analysis of SERPINA1. The desalted SERPINA1 was analysed on a modified Orbitrap Eclipse platform. The major proteoform of SERPINA1 with three sialylated bi-antennary N-glycans was selected (m/z 3928 with a window of m/z 20, charge state +13) and further fragmented with HCD (20 V). The MS/MS spectra were manually analysed using Xcalibur (version 4.1, ThermoFisher).

Proteomics analysis of glycoproteins and plasma samples. Glycoproteins were buffer-exchanged to 100 mM Tris buffer (pH 8.0) containing 8 M urea and 5 mM dithiothreitol (DTT) then incubated at 56 ºC for 20 min and buffer-exchanged to 100 mM Tris buffer (pH 8.0) with 20 mM iodoacetamide (IAA).

The samples were then alkylated at room temperature for 20 min in the dark and buffer-exchanged to 50 mM NH_4HCO_3 (pH 8.0). The glycoproteins were then transferred to a new Eppendorf tube and digested with trypsin at 37 ºC overnight for proteolytic digestion. The digested peptides were dried and reconstituted with 1% formic acid for LC-MS/MS analysis. The tryptic peptides (100 ng) were analyzed on a Dionex Ultimate 3000 UHPLC coupled to an Orbitrap Eclipse Tribrid mass spectrometer (Thermo Fisher Scientific). The peptides were firstly loaded onto a 75 µm×2 cm pre-column and separated on a 75 µm×15 cm Pepmap C18 analytical column (Thermo Fisher Scientific) with a binary buffer system. Buffer A was 0.1% formic acid (FA) in 100% H_2O and buffer B was 0.1% FA in 80% acetonitrile with 20% H_2O. The Eclipse mass spectrometer was operated in data-dependant acquisition mode with one full MS scan followed by MS/MS scans with higher-energy collision-induced dissociation (HCD) fragmentation.

Proteomics data analysis. The RAW data were processed with Maxquant (version 1.6.2) [2] for protein identification and PGlyco (version 2.0) [3] for glycopeptide identification. Quantification of the site-specific microheterogeneity was performed manually using Xcalibur (version 4.1). The extracted ion chromatogram (XIC) of each glycopeptide was processed with 50 ppm mass tolerance and a 7-point Gaussian smoothing. The area under the curve (AUC) was integrated for glycopeptide quantification.

Protein structure modelling. Protein structures of SERPINA1 (PDB: 3NE4), SERPINA3 (PDB: 6HGE), trypsin (PDB: 1QPA and 1OPH), elastase (PDB: 1BMA), chymotrypsin (PDB: 4CHA), Thrombin-SERPINC1-heparin complex (PDB: 1TB6), SERPIND1 (PDB: 1JMJ), SERPINE2 (PDB: 4DY0), SERPINA6 (PDB: 2V95), cathepsin G (PDB: 1KYN), chymase (PDB:1KLT) were retrieved from the PDB. SERPINA1, 2, 3, 5, 10, 12 in Figure S21 and TMPRSS2 in Figure S23 were retrieved from the AlphaFold protein structural database (alphafold.ebi.ac.uk). The protein structures were processed using University of California, San Francisco Chimera program (version 1.15 and version X 1.2.5) [4].

Sequence alignment. The protein sequences were retrieved from UNIPROT database. The protein sequences were introduced to Jalview program (version 2.11.2.2) [5] and aligned using T-coffee algorithm with default settings [6]. The aligned sequences were highlighted using Clustalx colour scheme.

Molecular dynamics simulation. The crystal structure of human SERPINA1 (PDB: 3NE4) was used as a template for the M1V variant. The M3 (E400D) variant structure was generated using PyMOL and the glycoprotein structures for MD simulation were generated using CHARM-GUI (http://www.charm-gui.org) [7,8]. The protein N-terminus and C-terminus were patched with acetylation and methylamidation, respectively. The bi-antennary N-glycans with α2,6-linked *N*-acetylneuraminic acid residues (the most abundant N-glycan on SERPINA1) were modelled on Asn46, Asn83 and Asn247 using Glycan Reader and Modeler [9]. The glycoproteins were then placed in a periodic box of TIP3P water molecules with 150 mM KCl. The box boundaries are 1.5 nm away from the glycoprotein. The

CHARMM36m force field was used for the polypeptide chain and carbohydrate residues. All simulations were performed at 303.15 K. After 5000 steps of energy minimization, all atoms were equilibrated for 200 ps under constant particle number, volume and temperature (NVT) conditions. The simulations were then performed using under constant particle number, pressure and temperature (NPT) conditions using GROMACS (version 2021) [10]. The temperature was maintained at 303.15 K using a Nose-Hoover thermostat with a time constant of 1 ps. A Parrinello-Raham barostat was employed for pressure regulation. Van der Waals interactions were treated using a forced-based switching function between 10 and 12Å. Long-range electrostatics were treated with the particle-mesh Ewald (PME) method. SHAKE was used to constrain all bonds involving hydrogen atoms. The data analysis (RMSD, RMSF and H-bonding) was performed using built-in functions GROMACS and VMD [11].

Glycan modelling on SERPINA3. A sialylated bi-antennary N-glycan was modelled onto Asn271 in SERPINA3 using GlycoSHIELD (version 0.1) following the protocol described in Park, S. J. *et al.* [12].

Glycan modelling on SERPINA1-TMPRSS2, SERPINA3-Chymas and SERPINA3-Cathepsin G complexes. Sialylated bi-antennary N-glycans were modelled on Asn70, Asn107 and Asn271 in SERPINA1 and Asn83, Asn106, Asn127, Asn186 and Asn271 in SERPINA3. Two sialylated bi-antennary N-glycans were modelled on Asn213 and Asn249 in TMPRSS2 extracellular domain. An M2 tetrasaccharide Manα1-6Manβ1-4GlcNAcβ1-4GlcNAc was modelled on Asn71 in cathepsin G. Two sialylated bi-antennary N-glycans were modelled on Asn80 and Asn103 in chymase. The protein structure files in PDB format were prepared using Chimera and CHARM-GUI. All glycan modelling was performed using GlycoSHIELD. The visualization of the glycoprotein complexes was performed using Chimera.

Database search for the SERPINA1 variants with E400D mutation (rs1303) in COVID-19 proteomics dataset. We analysed two single amino acid variants (SAVs) of SERPINA1, namely E400 (canonical) and D400 (rs1303) in a well-established plasma dataset from COVID-19 patients [13]. In brief, plasma samples from 13 patients diagnosed as severe symptoms, 16 patients diagnosed as mild symptoms, and 13 healthy donors at Wuhan Jinyintan Hospital were used in this study. The plasma samples were digested with trypsin protease, labelled with 11-plex tandem mass tag (TMT), fractionated with strong cation exchange chromatography (SCX) and processed with liquid chromatography with tandem mass spectrometry (LC-MS/MS). To probe the SERPINA1 protein expression level in each sample, we first searched the quantitative MS dataset using Maxquant (version 2.0.3) [2] with an in-house constructed human proteome database (UNIPROT:UP000005640) with SARS-CoV-2 proteome [13]. We identified 860 human proteins in this dataset. We then retrieved the SAV information of these 860 identified human proteins from the Single Nucleotide Polymorphism database (dbSNP) and constructed a tailored FASTA file containing the sequences of these 860 human proteins with SAV information. We performed a second-round database search using Maxquant with the tailored FASTA file. The “variation mode” was enabled in Maxquant to search the SAV peptides. We manually

checked the identification of SAV peptides of SERPINA1 (E400 and D400) and extracted the TMT ratios for the following analysis.

Haplotyping SERPINA1 variants in the COVID-19 dataset. We determined the SERPINA1 haplotypes of each donor in COVID-19 dataset based on the TMT ratios of SAV peptides. We focused on two tryptic peptides containing the mutation site (E400D), namely FNKPFVFLMIEQNTK (E400) and FNKPFVFLMIDQNTK (D400). Both Glu (E) and Asp (D) carry a carboxy group in the side chain. The mutation of E to D does not introduce a significant change to the MS responses of these two peptides. Therefore, the ratio of FNKPFVFLMIEQNTK (E400) and FNKPFVFLMIDQNTK (D400) represents the relative abundances of M1V and M3 variants in each sample. The ratio of E400 to D400 can be calculated as:

$$r = \log_2\left(\frac{\text{TMT ratio}_{\text{FNKPFVFLMIEQNTK}}}{\text{TMT ratio}_{\text{FNKPFVFLMIDQNTK}}}\right)$$

We haplotyped SERPINA1 in each individual based on the ratio of E400 and D400 (r). We used an empirical value of 0.585 ($log_2 1.5$) as the threshold:

	SERPINA1 haplotype
$r < -0.585$	D400/D400
$-0.585 < r < 0.585$	E400/D400
$r > 0.585$	E400/E400

Using this approach, we determined the 14 individuals with SERPINA1$^{E400/E400}$, 17 individuals with SERPINA1$^{D400/D400}$ and 3 heterozygous individuals with SERPINA1$^{E400/D400}$. We further extracted the SERPINA1 abundance information from homozygous individuals (SERPINA1$^{E400/E400}$ and SERPINA1$^{D400/D400}$) to investigate E400 and D400 expression levels in COVID-19 patients.

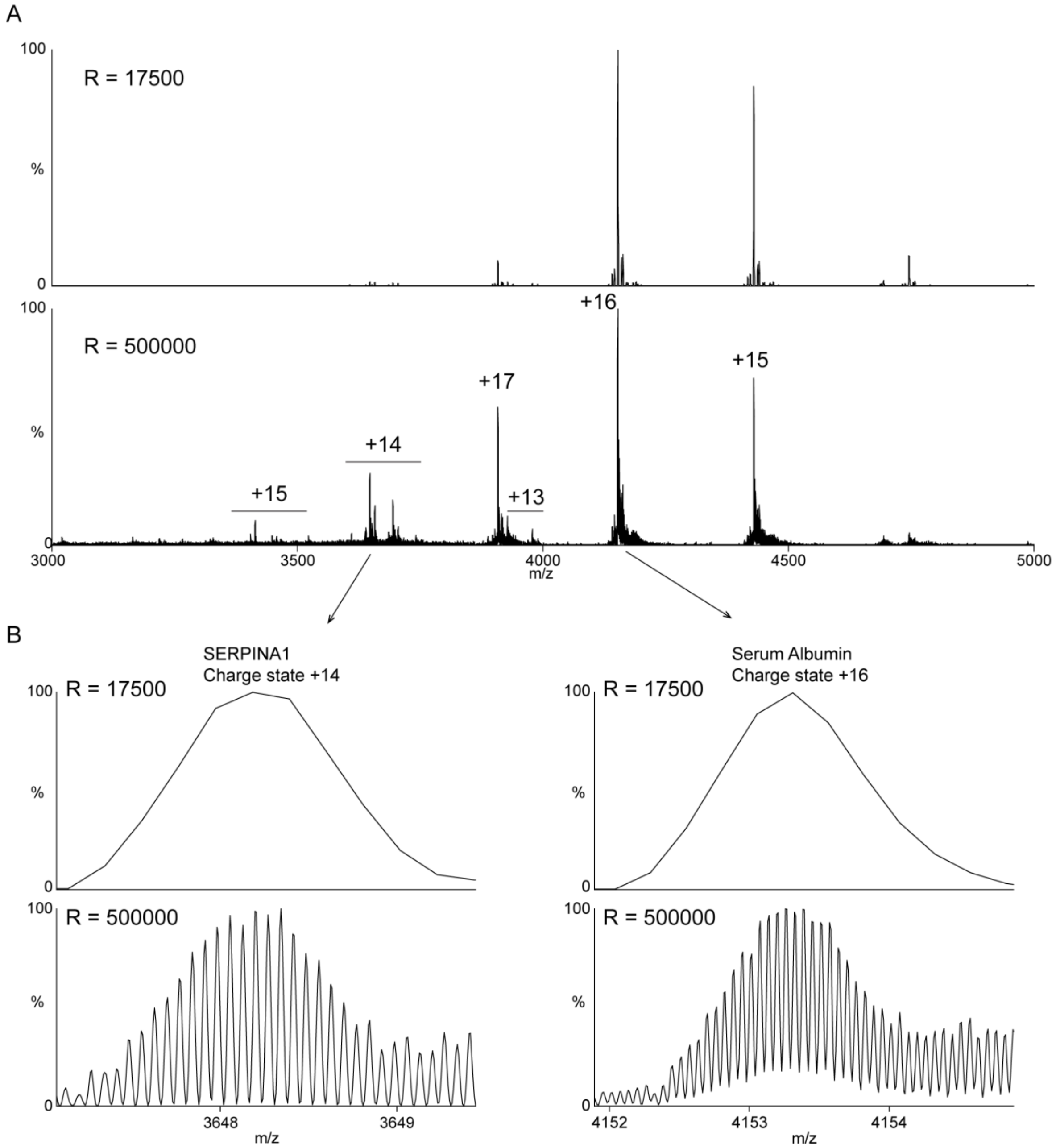

Figure S1. High-resolution native MS analysis of human plasma proteins. A) High-resolution native mass spectra of non-depleted human plasma sample from an individual donor with resolving power (R) of 17500 and 500000. B) High-resolution native MS (resolving power of 500000) recorded isotopic peaks of SERPINA1 and serum albumin.

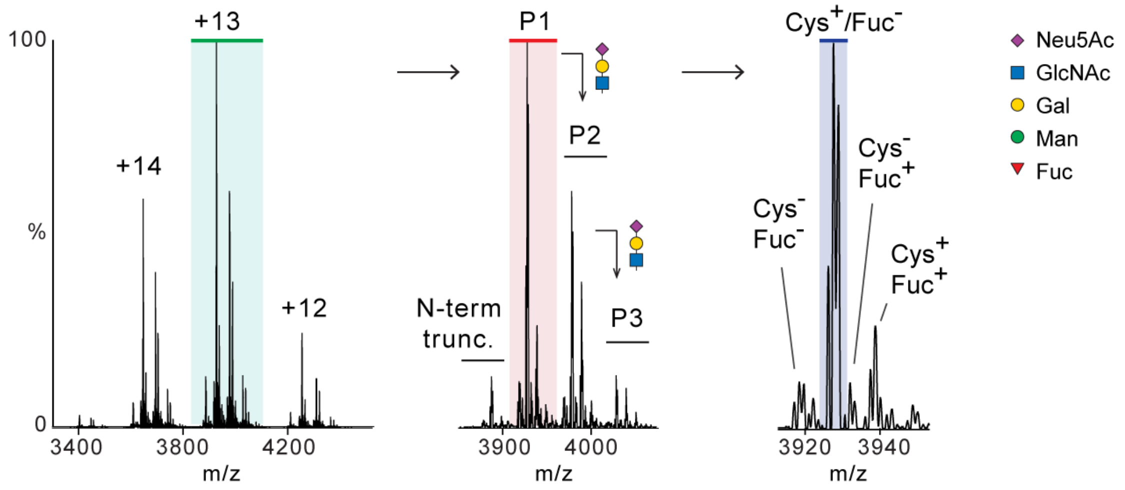

Figure S2. Native MS analysis of SERPINA1. Native MS revealed peaks consistent with N-glycan branching (addition of GlcNAc-Gal-Neu5Ac units, P1 to P3 series), N-terminal truncation, fucosylation (Fuc) and cysteinylation (Cys) status.

A

Variant	Theoretical Mass
M2	51028.10
M1A	51033.12
M3	51047.15
M1V	51061.17

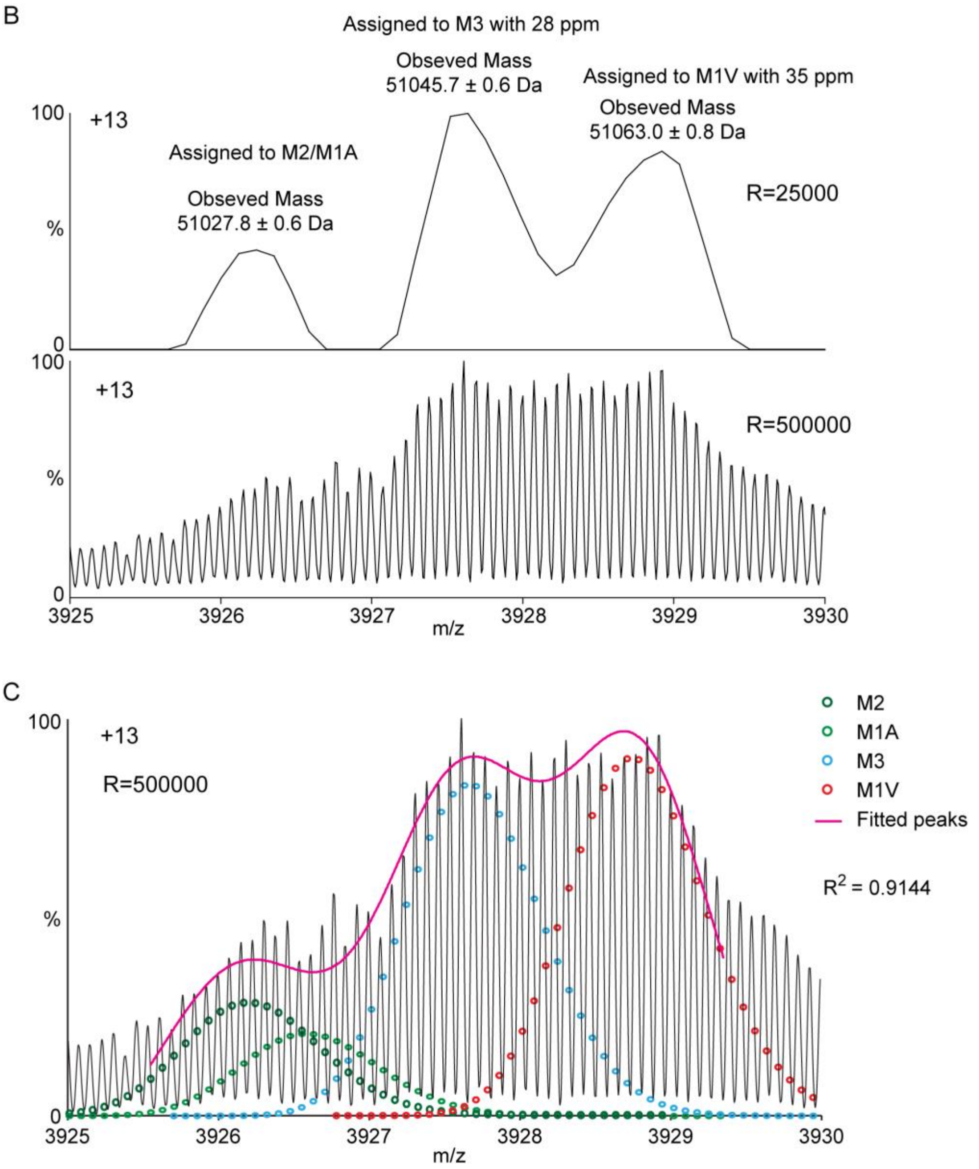

Figure S3. High-resolution native MS analysis of SERPINA1. A) Average masses of SERPINA1 main proteoforms. B) High-resolution mass spectra of SERPINA1 with resolving power of 25000 and 500000. Two major peaks (51045.7 Da and 51063.0 Da) were assigned to M3 and M1V variants, respectively. The smallest peak (51027.8 Da) was assigned to the overlapped M2 and M1A variants. C) theoretical isotopic peaks of M1V, M1A, M2 and M3 were fitted to the high-resolution native mass spectrum of SERPINA1. The coefficient of determination (R^2) of the fitting is 0.9144, suggesting a good correlation between the recorded and fitted peaks.

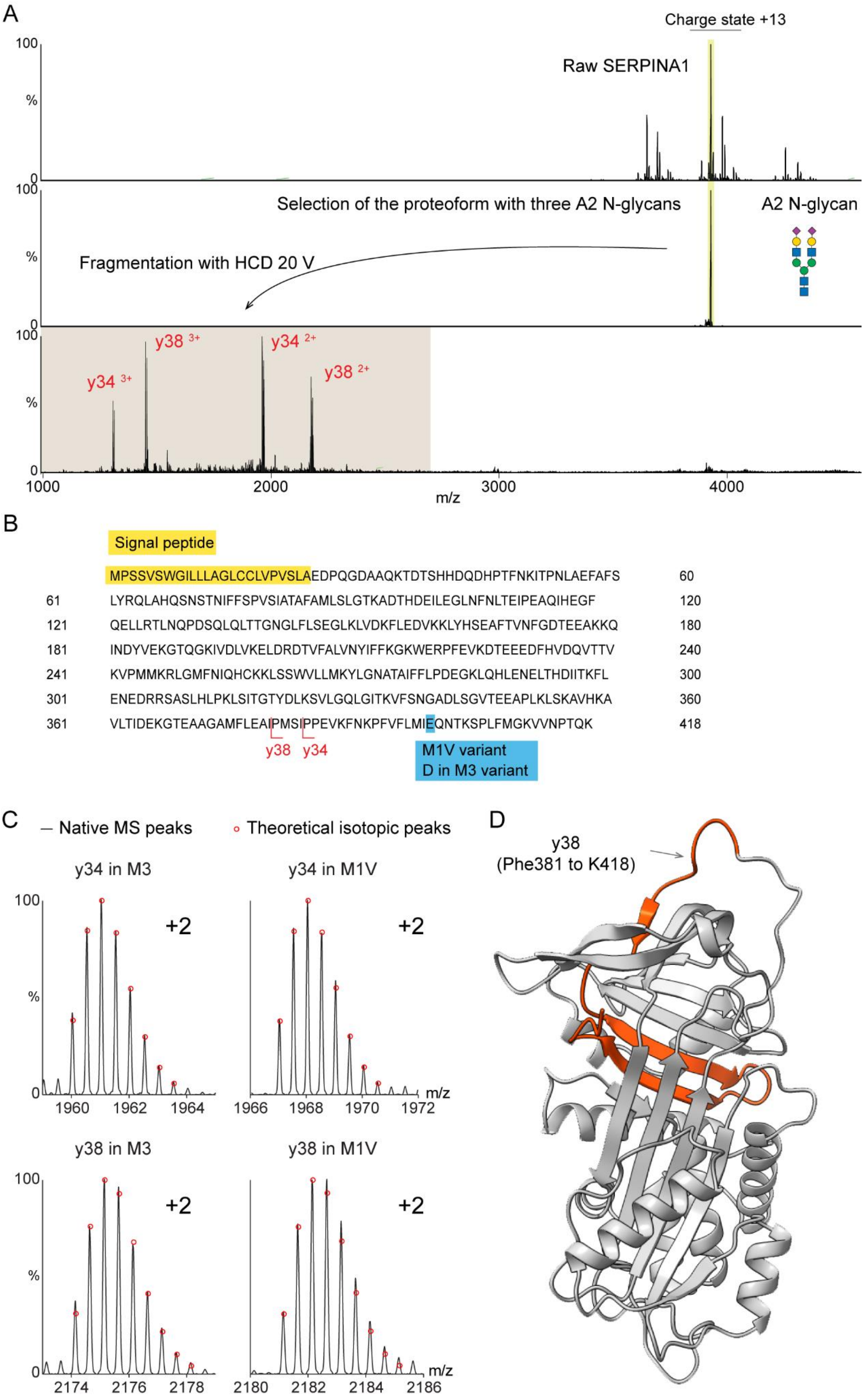
A
Charge state +13
Raw SERPINA1
Selection of the proteoform with three A2 N-glycans
A2 N-glycan
Fragmentation with HCD 20 V
y34 3+
y38 3+
y34 2+
y38 2+
100
%
0
1000
2000
3000
4000
m/z
B
Signal peptide
MPSSVSWGILLLAGLCCLVPVSLAEDPQGDAAQKTDTSHHDQDHPTFNKITPNLAEFAFS 60
61 LYRQLAHQSNSTNIFFSPVSIATAFAMLSLGTKADTHDEILEGLNFNLTEIPEAQIHEGF 120
121 QELLRTLNQPDSQLQLTTGNGLFLSEGLKLVDKFLEDVKKLYHSEAFTVNFGDTEEAKKQ 180
181 INDYVEKGTQGKIVDLVKELDRDTVFALVNYIFFKGKWERPFEVKDTEEEDFHVDQVTTV 240
241 KVPMMKRLGMFNIQHCKKLSSWVLLMKYLGNATAIFFLPDEGKLQHLENELTHDIITKFL 300
301 ENEDRRSASLHLPKLSITGTYDLKSVLGQLGITKVFSNGADLSGVTEEAPLKLSKAVHKA 360
361 VLTIDEKGTEAAGAMFLEAIPMSIPPEVKFNKPFVFLMIEQNTKSPLFMGKVVNPTQK 418
y38 y34
M1V variant
D in M3 variant
C
Native MS peaks
Theoretical isotopic peaks
y34 in M3
y34 in M1V
y38 in M3
y38 in M1V
+2
1960 1962 1964
1966 1968 1970 1972
2174 2176 2178
2180 2182 2184 2186
m/z
D
y38
(Phe381 to K418)

Figure S4. Native top-down MS analysis of SERPINA1. A) The SERPINA1 proteoform with three fully sialylated bi-antennary N-glycan (A2) was selected and further fragmented with HCD (20 V). The y34 and y38 fragment ions were detected. No glycan fragments were observed. B) The sequence of SERPINA1. Both y34 and y38 fragments cover the C-terminal residues, including the D400/E400 which is the only different amino acid between M1V and M3 variants. C) Mass spectra of SERPINA1 fragments (y34 and y38) from the native top-down analysis. Theoretical ion distributions are indicated by the red dots. D) Structure of SERPINA1. The y38 fragment is highlighted in orange. It covers part of the RCL.

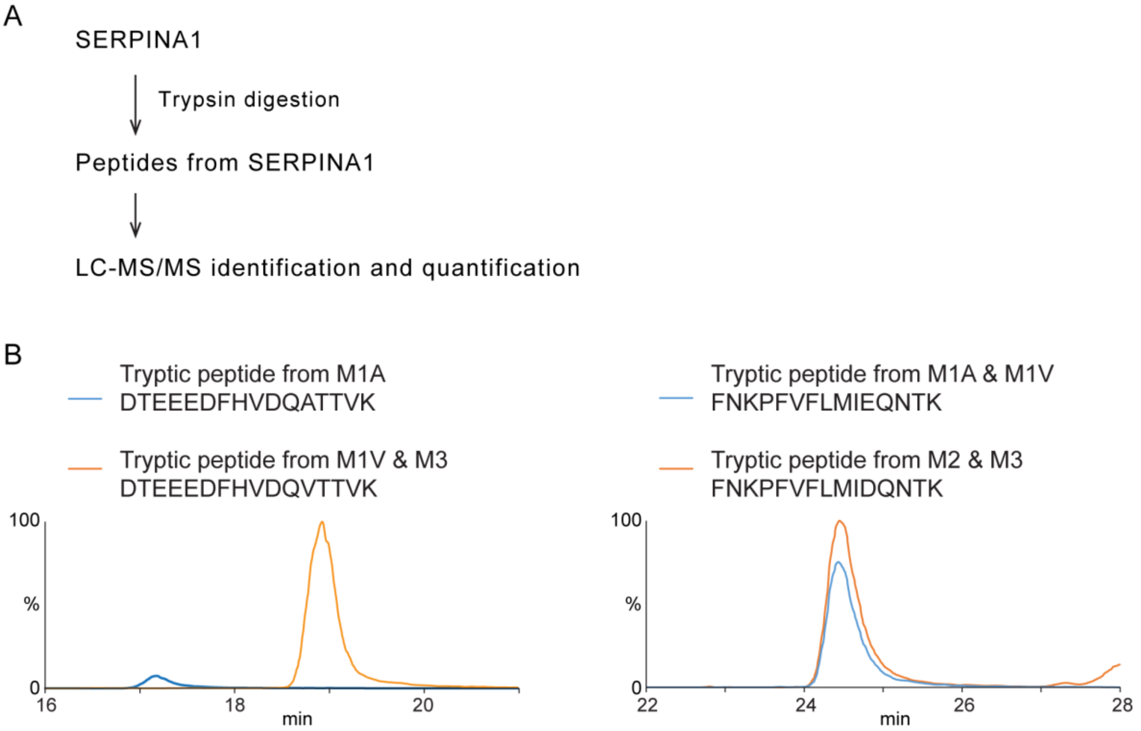

C

Variant	Mutations	dbSNP ID	Amino acid (Codon)	Frequency
M1V	Wild-type	rs6647	V237A (GTG>GCG)	GCG=0.22
M1A	V237A	rs709932	R125H (CGT>CAT)	CAT=0.16
M2	R125H/E400D	rs1303	E400D (GAA>GAC)	GAC=0.25
M3	E400D			

Figure S5. Proteomics analysis of SERPINA1. A) Flow chart of the proteomics analysis of SERPINA1. B) Extracted ion chromatograms (EICs) of the corresponding tryptic peptides to each variant. The tryptic peptide with Val237Ala mutation (DTEEEDFHVDQATTVK) is unique to M1A variant. The other tryptic peptides are not unique to each variant. C) The dbSNP ID and frequency of M1V, M1A, M2 and M3 variants.

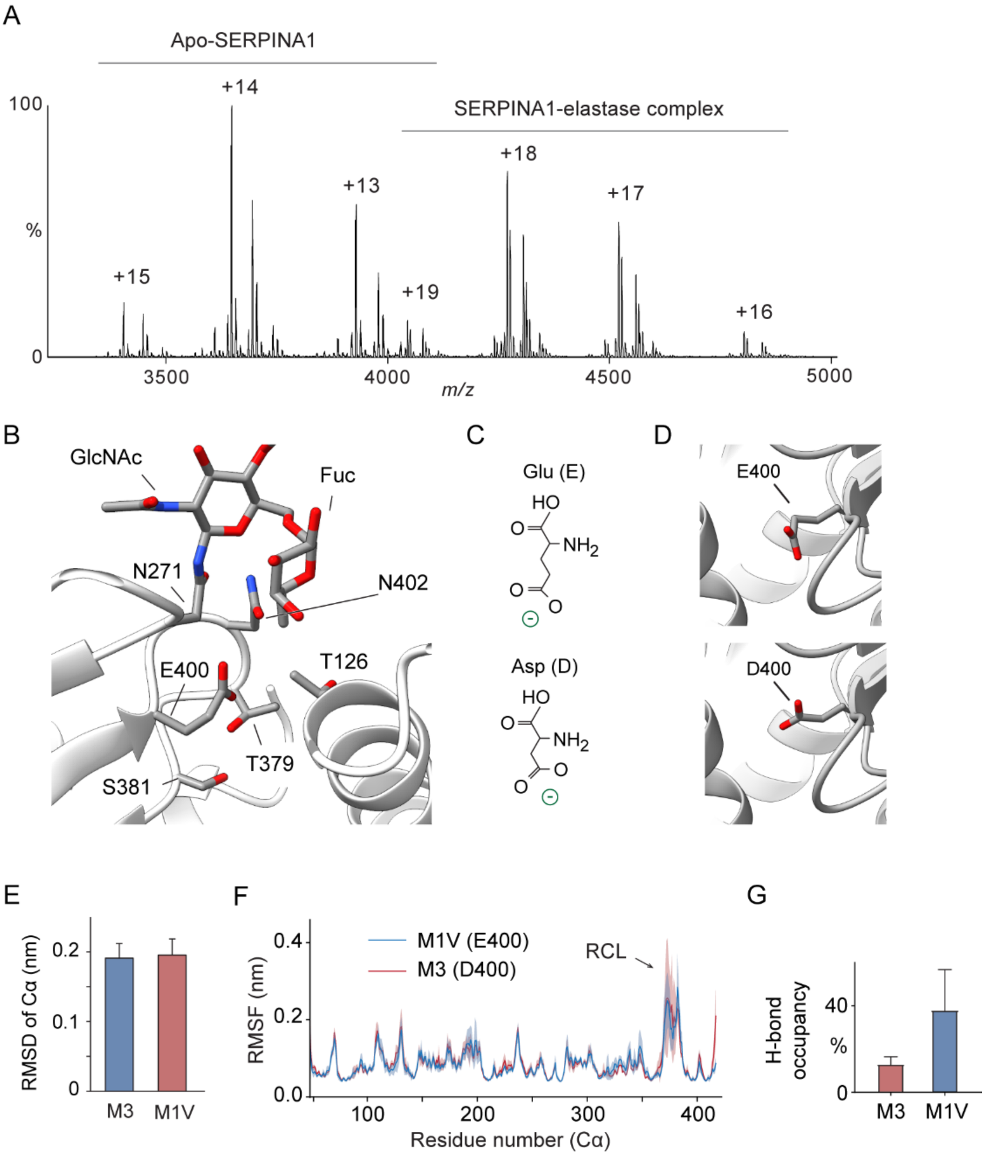

Figure S6. Structural analysis of SERPINA1. A) Native mass spectrum of SERPINA1-elastase complexes. The apo-SERPINA1 and SERPINA1-elastase complex peaks are labelled with the corresponding charge states. B) The side chain of E400 (M1V variant) can form hydrogen bonds with the surrounding amino acid residues (T126, T379, S381, T402). C) Structure of glutamic acid (Glu) and aspartic acid (Asp). D) Structure comparison of Glu400 (M1V variant) and Asp400 (M3 variant). E) The Cα atom root-mean-square deviation (RMSD) plot of M1V and M3 in MD simulations. F) The Cα atom root-mean-square fluctuation (RMSF) plot of M1V and M3 in MD simulations. The 95% confidence intervals for M1V and M3 variants are highlighted in light red and light blue respectively. G) The hydrogen bond (H-bond) occupancies of the side chains of Glu400 (M1V variant) and Asp400 (M3 variant) interacting with Thr126, Thr379, Ser381 and Asn402 in MD simulation.

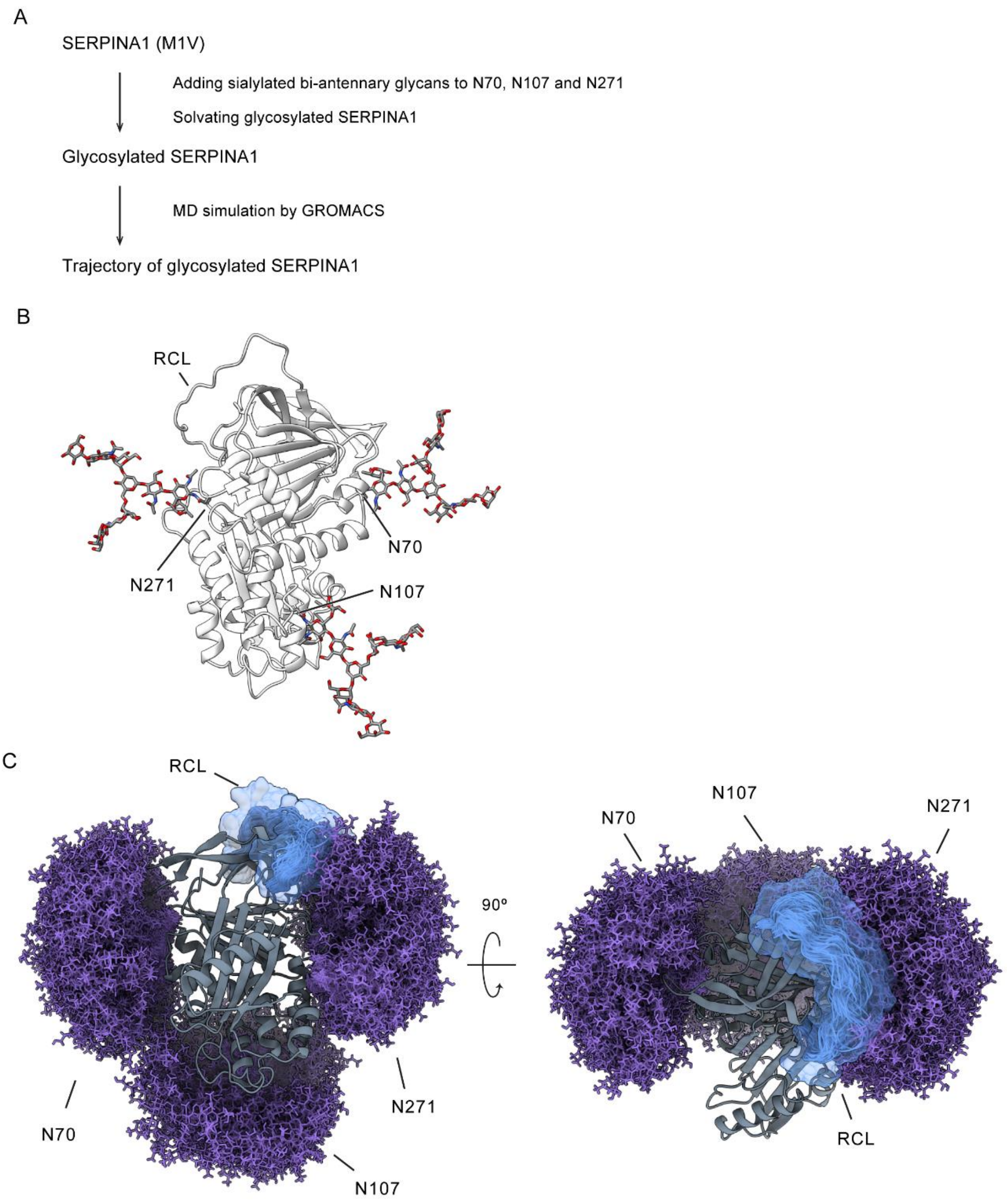

Figure S7. MD simulations of SERPINA1. A) Flow chart of molecular dynamics simulation for glycosylated SERPINA1. B) a modeled SERPINA1 structure with three sialylated bi-antennary N-glycans on N70, N107 and N271. C) Stereo-view of the structure of glycosylated SERPINA1 from the 150 ns MD simulation trajectory simulation. The snapshots of the three N-glycans and RCL conformations (1 frame per ns) are extracted and overlayed.

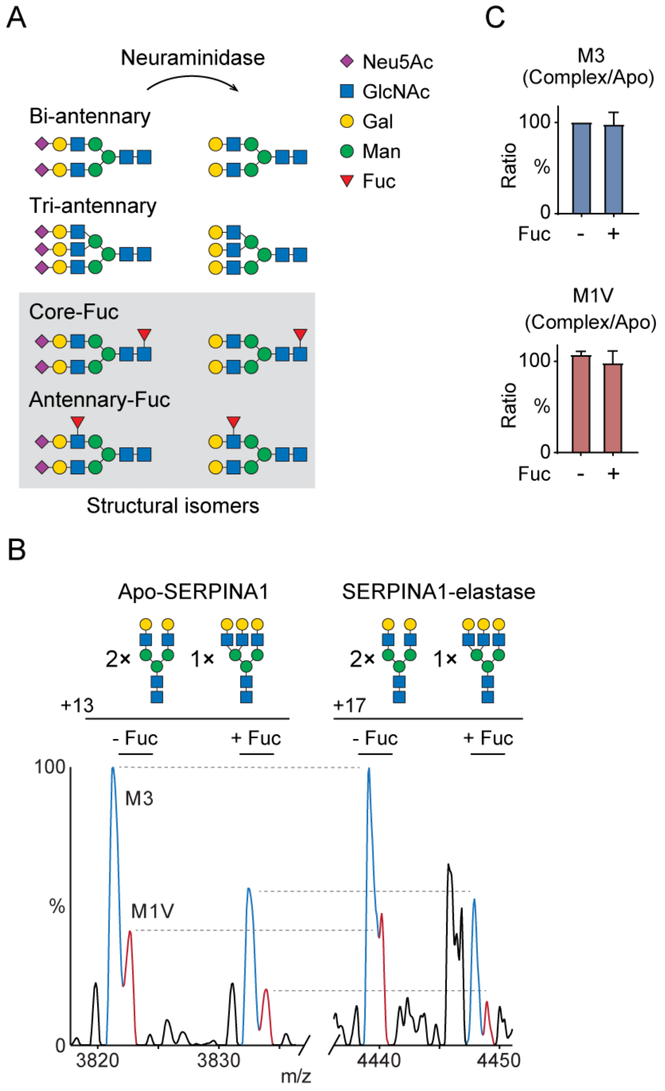

Figure S8. Native MS analysis of desialylated SERPINA1. A) Neuraminidase treatment releases all terminal Neu5Ac residues from N-glycans. However, this treatment cannot distinguish isomeric core- and antennary fucosylated N-glycans. B) Native mass spectra of the desialylated SERPINA1 variants M3 and M1V in apo forms and complex forms with elastase. The N-glycan composition and variants are labeled. C) Bar graphs of the ratio of desialylated SERPINA1-elastase complex to desialylated apo-SERPINA1, with and without fucosylation. Bars show mean ± standard deviation from three individual replicates.

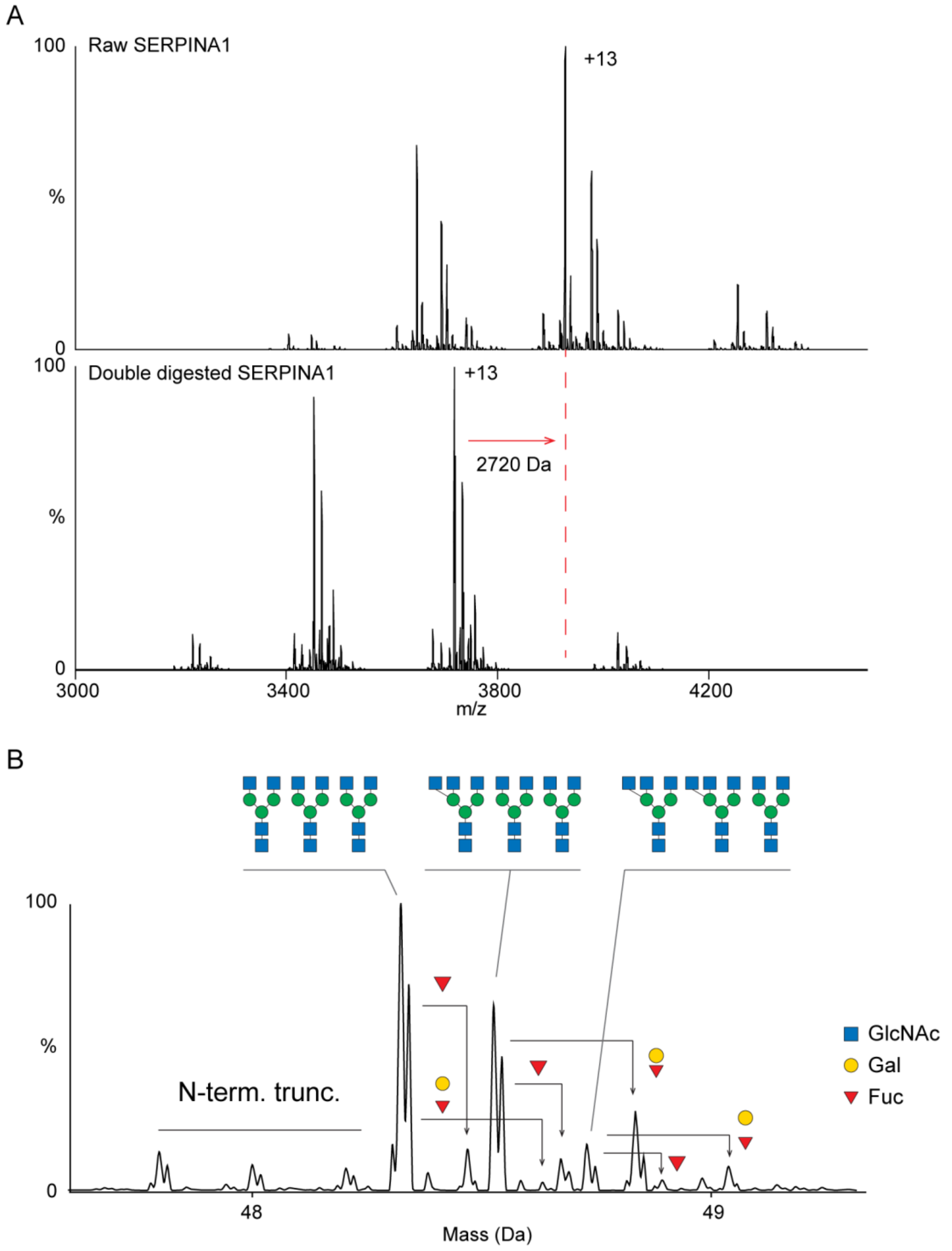

Figure S9. Native MS analysis of double digested SERPINA1. A) Native mass spectra of double exoglycosidase digested SERPINA1 (neuraminidase and galactosidase treated) and raw SERPINA1. All peaks in the double digested SERPINA1 spectrum shift to lower m/z values compared to the untreated form. All Neu5Ac and Gal residues from SERPINA1, are removed during double digestion resulting in a loss of 2720 Da (six Neu5Ac-Gal units) to the main species (base peak). B) Annotation of the zero-charge spectrum of double exoglycosidase digested SERPINA1. We assigned major peaks to the corresponding proteoforms and confirmed complete double digestion.

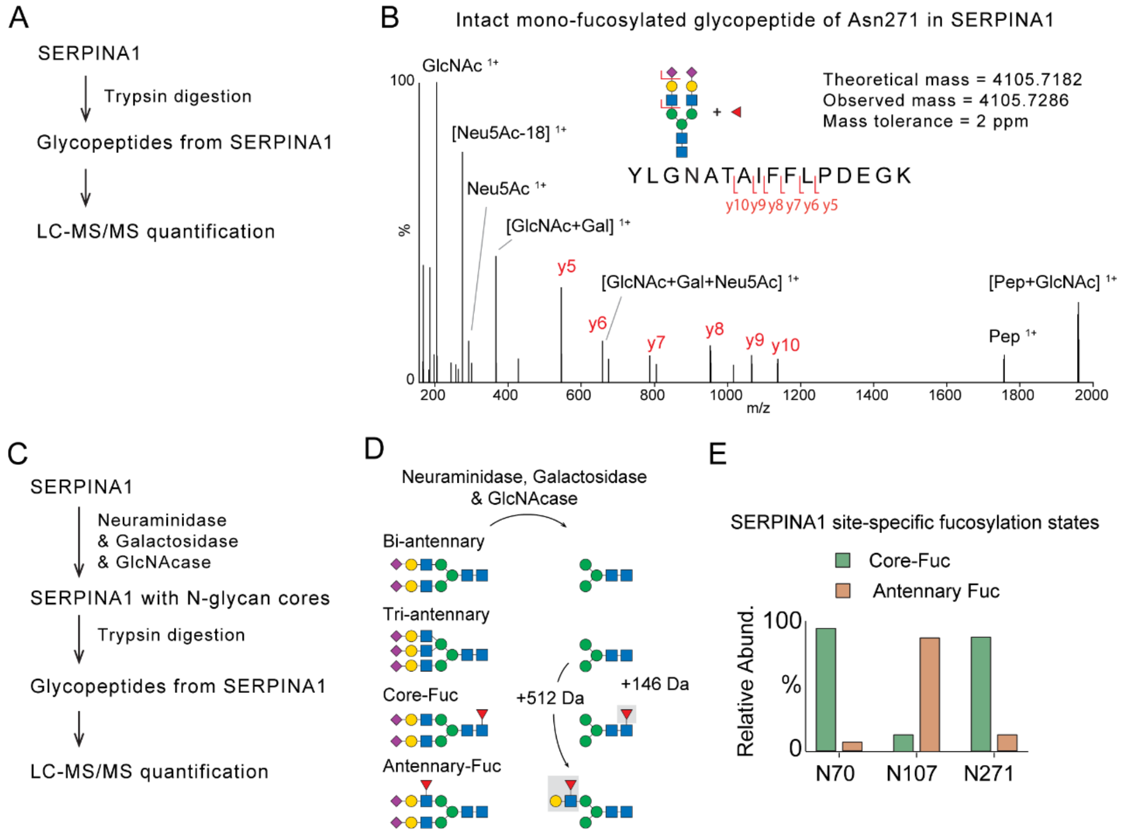

Figure S10. Glycoproteomics analysis of SERPINA1 site-specific fucosylation. Firstly, we performed glycoproteomics of raw SERPINA1 (panel A). We identified the intact mono-fucosylated glycopeptide of Asn271 in SERPINA1 based on the MS/MS spectrum (panel B). We confirmed the presence of fucosylation on Asn271. However, we couldn’t distinguish core- or antennary fucosylation based on the MS/MS spectrum. Therefore, we digested SERPINA1 with neuraminidase, galactosidase and GlcNAcase sequentially, and performed glycoproteomics analysis with the triple exoglycosidase digested SERPINA (panel C). The triple exoglycosidase digestion transformed the core and antennary fucosylated N-glycans to different structures (panel D). This enables us to identify and quantify core and antennary fucosylation on each glycosylation site in SERPINA1. Using this strategy, we identified and quantified the core- and antennary fucosylation across all three sites (panel E). To compare the fucosylation levels across all three sites, the total fucosylated (core and antennary fucosylated) tryptic peptide intensities of each site were normalized to 100%. Bar graphs show the relative abundances of core- and antennary fucosylation on Asn70, Asn107 and Asn271. This suggests all three N-glycosylation sites can be either core or antennary fucosylated. Moreover, core-fucosylation dominates Asn70 and Asn271.

A

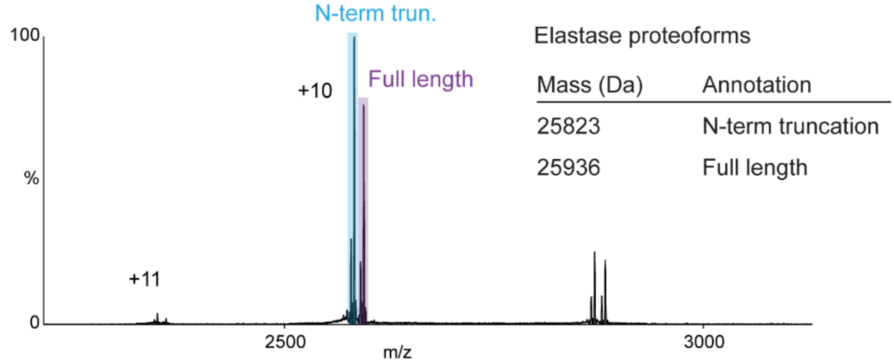

Elastase proteoforms

Mass (Da)	Annotation
25823	N-term truncation
25936	Full length

B

SERPINA1 proteoforms

Mass (Da)	Annotation
48327	HexNAc12Hex9
48473	HexNAc12Hex9Fuc1
48635	HexNAc12Hex10Fuc1
48530	HexNAc13Hex9
48676	HexNAc13Hex9Fuc1
48838	HexNAc13Hex10Fuc1
48984	HexNAc13Hex10Fuc2
48733	HexNAc14Hex9
48879	HexNAc14Hex9Fuc1
49041	HexNAc14Hex10Fuc1

C

SERPINA1 with N-term trunc Elastase

Mass (Da)	Annotation
74150	HexNAc12Hex9
74296	HexNAc12Hex9Fuc1
74458	HexNAc12Hex10Fuc1
74353	HexNAc13Hex9
74499	HexNAc13Hex9Fuc1
74661	HexNAc13Hex10Fuc1
74807	HexNAc13Hex10Fuc2
74556	HexNAc14Hex9
74702	HexNAc14Hex9Fuc1
74864	HexNAc14Hex10Fuc1

8 Da

8 Da

SERPINA1 with full length Elastase

Mass (Da)	Annotation
74263	HexNAc12Hex9
74409	HexNAc12Hex9Fuc1
74571	HexNAc12Hex10Fuc1
74466	HexNAc13Hex9
74612	HexNAc13Hex9Fuc1
74774	HexNAc13Hex10Fuc1
74920	HexNAc13Hex10Fuc2
74669	HexNAc14Hex9
74815	HexNAc14Hex9Fuc1
74977	HexNAc14Hex10Fuc1

Figure S11. Native MS analysis of elastase. A) Native mass spectrum of elastase. The N-terminal truncated and full-length forms are highlighted in blue and purple, respectively. B) Theoretical molecular weight and monosaccharide composition of double exoglycosidase digested SERPINA1 glycoforms. C) Theoretical molecular weight of double exoglycosidase digested SERPINA1 complexed with elastase. The major glycoforms suitable for native MS quantification are labelled in red. We highlighted two pairs of unresolvable glycoforms in yellow and green, respectively. Native MS cannot resolve two 74 kD proteins differing with 8 Da [14]. Therefore, we cannot accurately quantify fucosylation regulation to double exoglycosidase treated SERPINA1-elastase complexes using native MS analysis.

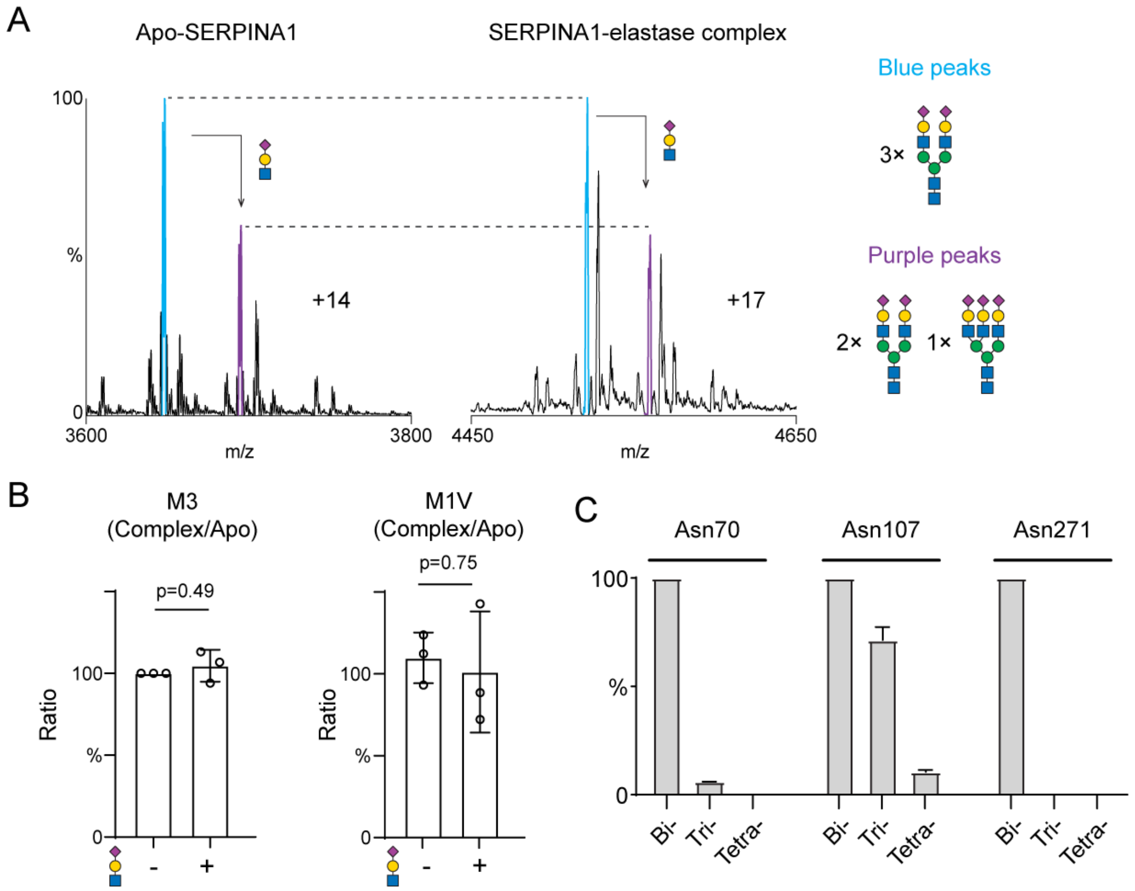

Figure S12. MS analysis of N-glycan branching regulation to SERPINA1-elastase interactions. A) Native mass spectrum of apo-SERPINA1 and SERPINA1-elastase complexes. The peaks are labelled with the corresponding charge states. apo-SERPINA1 and SERPINA1-elastase complex peaks with three bi-antennary N-glycans are highlighted in blue. The peaks with two bi-antennary and one tri-antennary N-glycan are highlighted in purple. B) Bar graphs of the ratio of the SERPINA1-elastase complex to apo-SERPINA1, with and without the tri-saccharide Neu5Ac-Gal-GlcNAc unit. Bars show mean ± standard deviation with dots from three independent experiments. A student's t-test was performed to calculate the p value. C) Glycoproteomics analysis of the site-specific N-glycan branching level. Bar graphs of the relative abundance of bi-, tri- and tetra- antennary N-glycans at Asn70, Asn107 and Asn271. Bars show mean ± standard deviation from three independent glycoproteomics experiments.

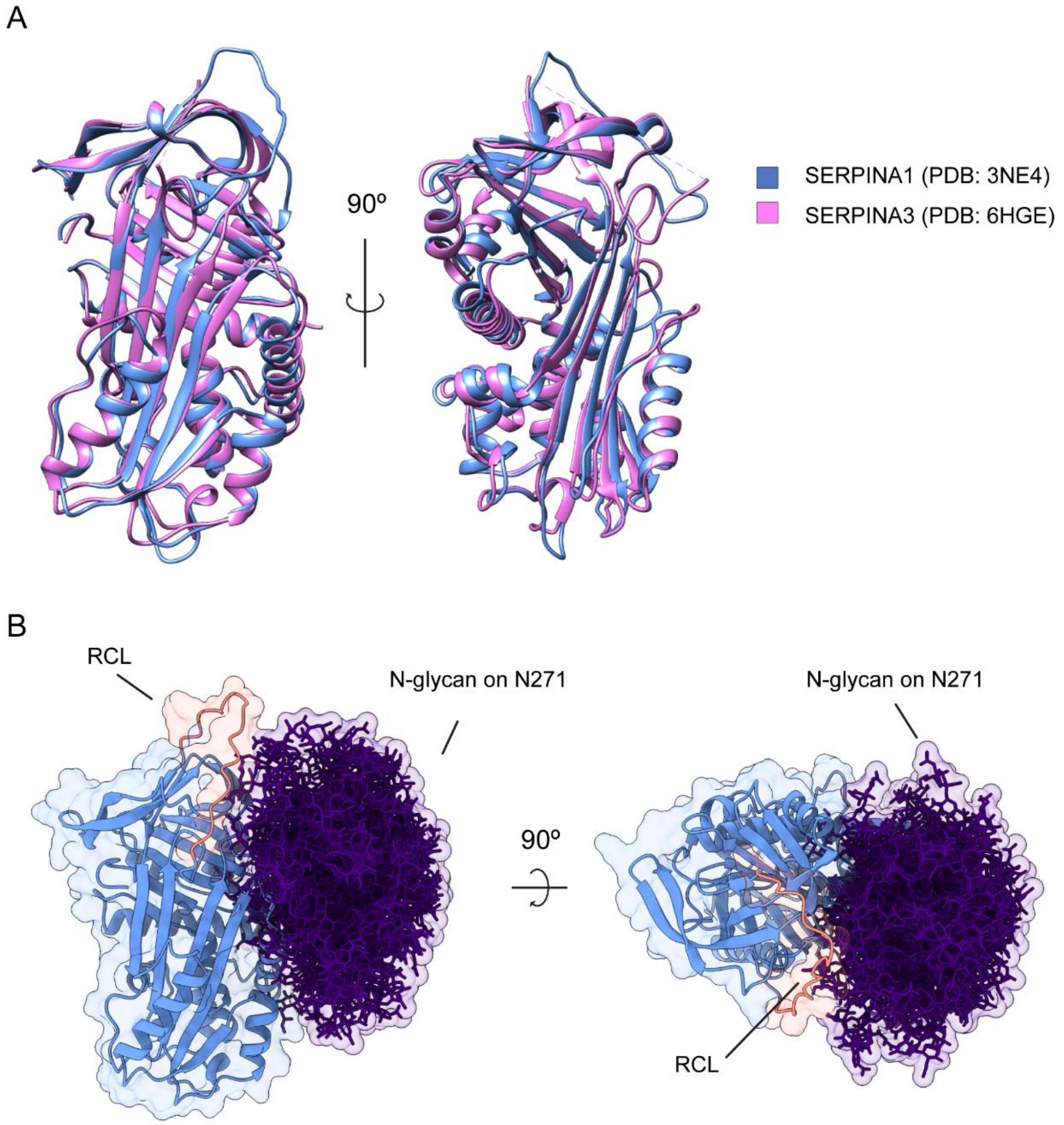

Figure S13. Structural analysis of SERPINA3. A) Structural comparison of SERPINA1 and SERPINA3. The structure superimposing of SERPINA1 (PDB: 3NE4) and SERPINA3 (PDB: 6HGE) were performed using UCSF Chimera program. B) The relative positions of reactive center loop (RCL) and N-glycan on Asn271 in SERPINA3. The RCL is highlighted in orange. The N-glycan conformers on Asn271 were simulated with GlycoSHIELD program using a sialylated bi-antennary N-glycan as a model. The possible N-glycan conformers on Asn271 are overlayed and shown in purple.

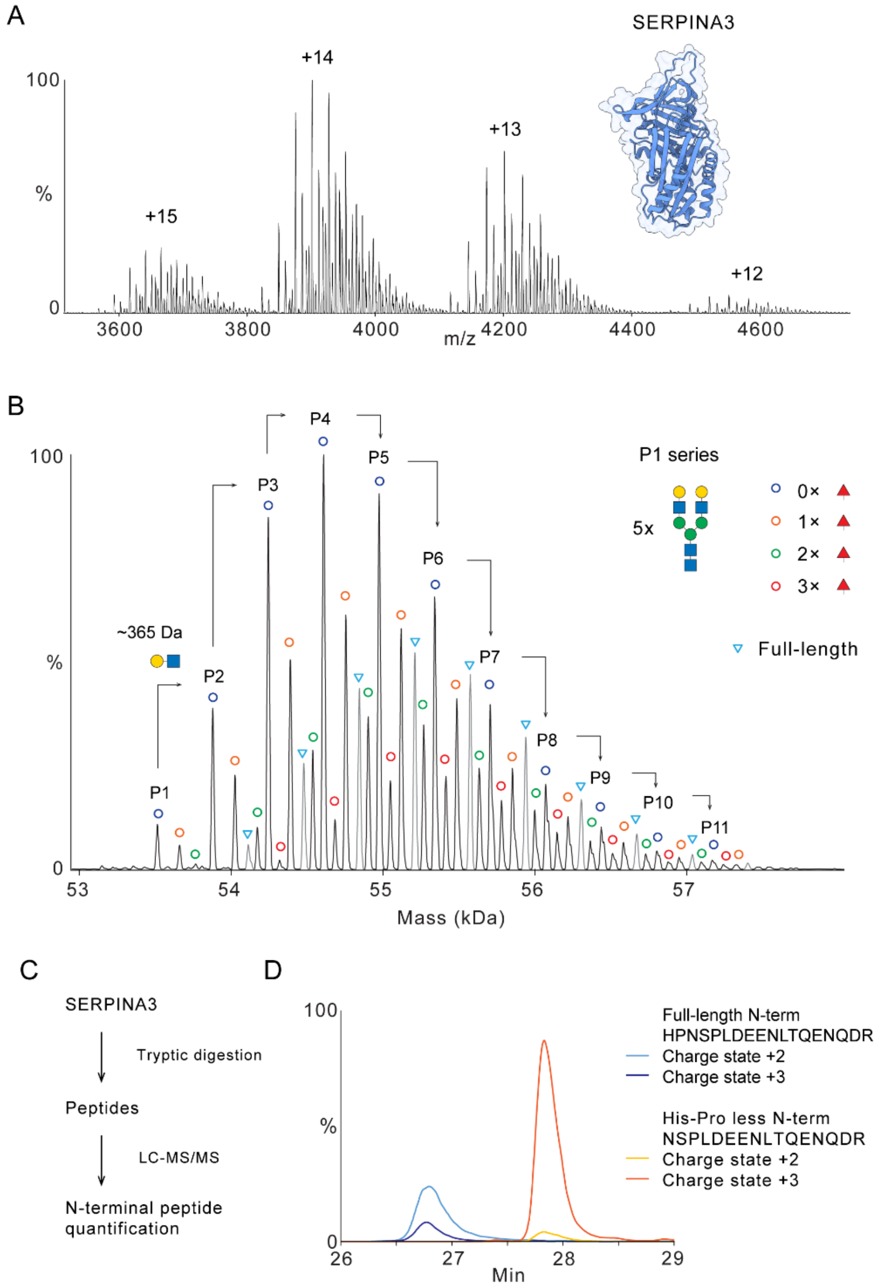

Figure S14. MS analysis of SERPINA3. A) Native mass spectrum of desialylated SERPINA3. B) Annotation of the desialylated SERPINA3 proteoforms. The N-glycan branching (addition of Gal-GlcNAc units, P1 to P11 peak series) and fucosylation (addition of fucose residues) are labelled. C) The flow chart of proteomics analysis of SERPINA3 N-terminal truncation. D) The extracted ion chromatograms of the tryptic peptides from full-length and N-terminal truncated (His-Pro less variant) SERPINA1.

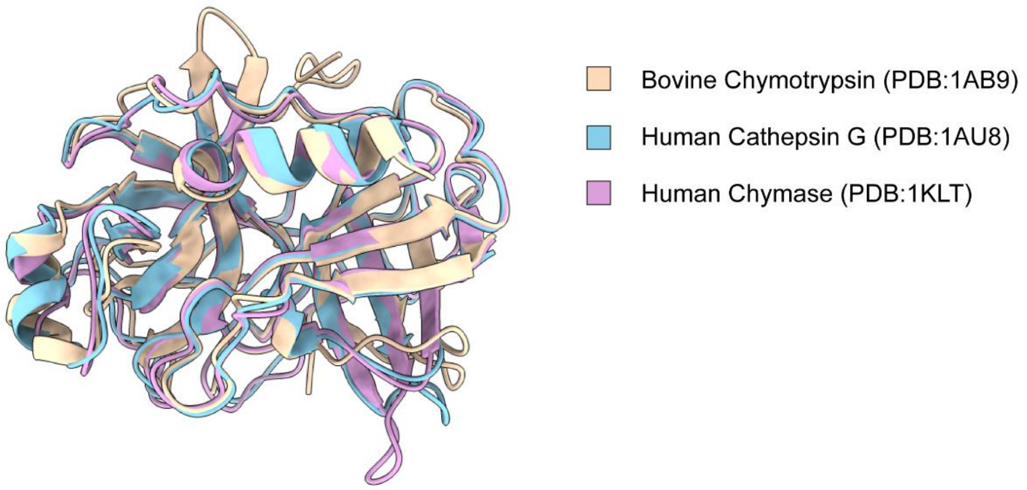

Figure S15. Structural comparison of bovine chymotrypsin (PDB:1AB9), human cathepsin G (PDB: 1AU8) and human chymase (PDB: 1KLT). The structural alignment was performed using UCSF Chimera program.

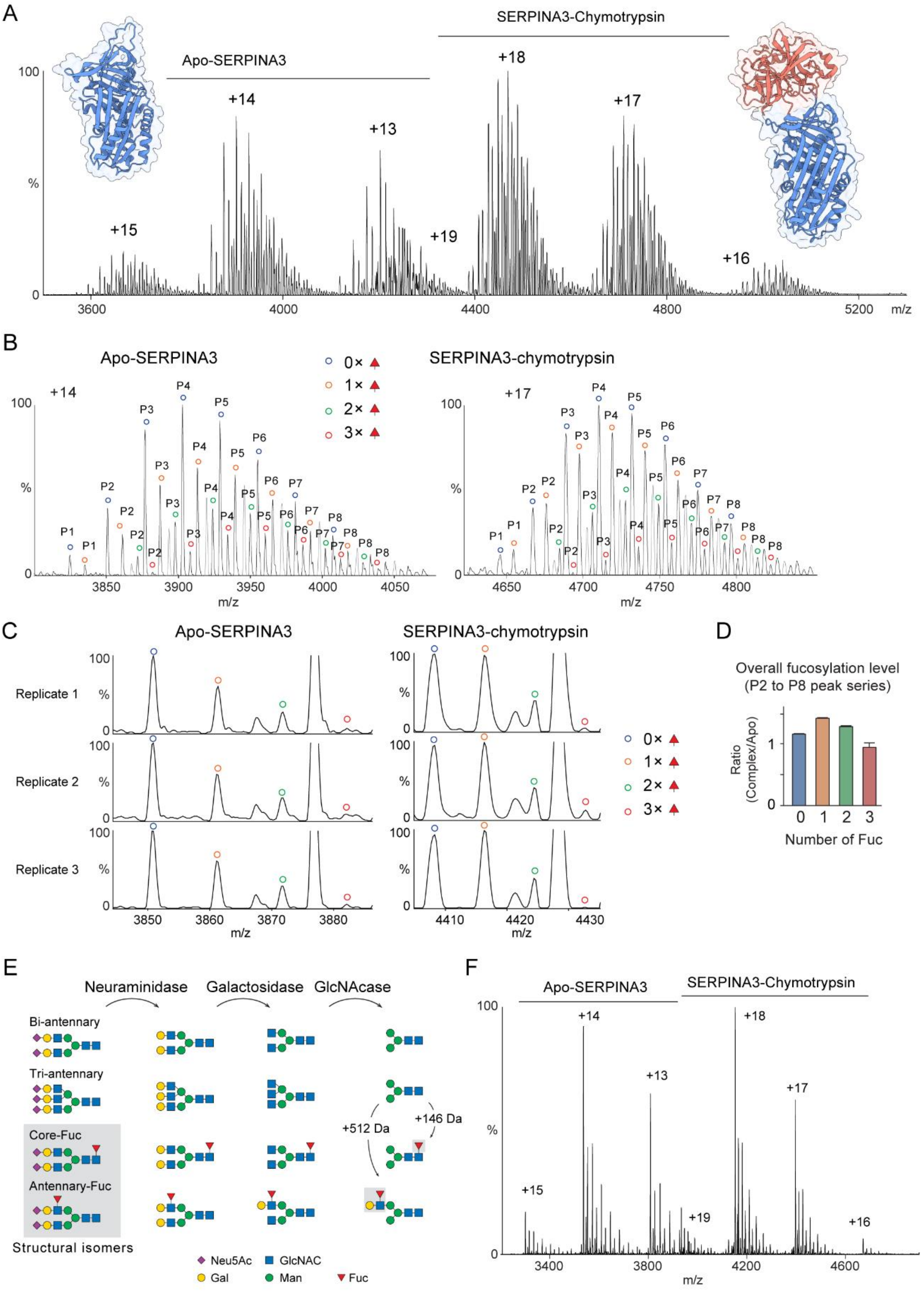

Figure S16. Native MS analysis of SERPINA3-chymotrypsin complexes. A) Native mass spectrum of SERPINA3-chymotrypsin complexes. B) annotation of the proteoforms of SERPINA3 and SERPINA3-chymotrypsin complexes. Fucosylation statuses, namely non-, mono-, bi- and tri- fucosylation are labelled with blue, orange, green and red circles, respectively. Peaks with the same hexose (Hex) and

N-acetylhexosamine (HexNAc) numbers are labeled P1 to P8. C) Native mass spectra of P2 series of apo-SERPINA3 and SERPINA3-chymotrypsin complexes. The spectra from three replicates are shown. Fucosylation statuses, namely non-, mono-, bi- and tri- fucosylation are labelled with blue, orange, green and red circles, respectively. D) Bar graph of the ratio of the complex to apo protein with 0 to 3 fucose residues. Bars show mean±s.d. from three independent experiments. E) Triple exoglycosidase treatment of SERPINA3 to distinguish core- and antennary fucosylation. F) The native MS spectrum of triple exoglycosidase treated SERIPNA3 and SERPINA3-chymotrypsin complexes.

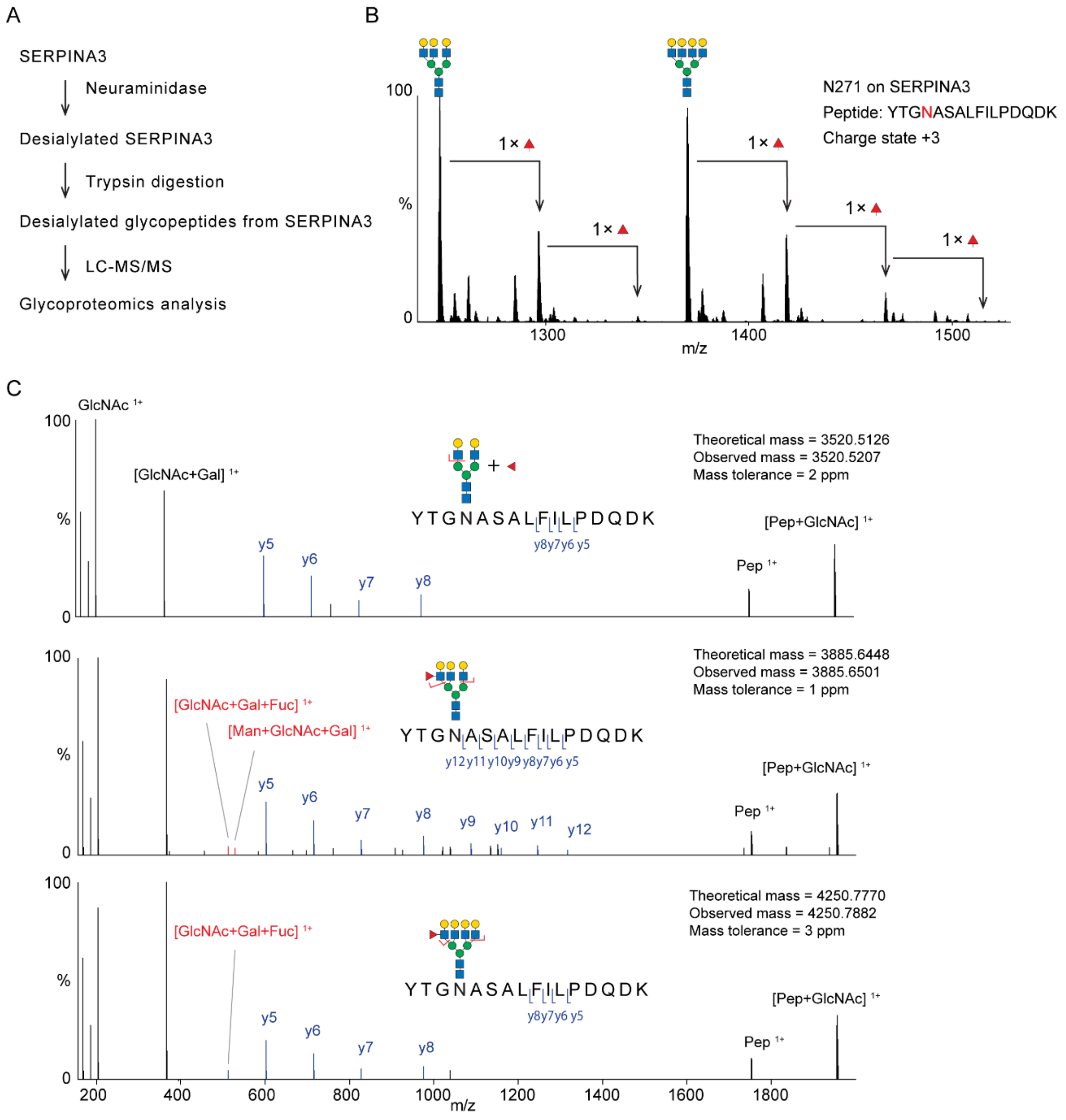

Figure S17. Glycoproteomics analysis of SERPINA3. A) Flow chart of the glycoproteomics analysis of desialylated SERPINA3. B) The mass spectrum of the glycopeptide (YTGNASALFILPDQDK) primarily carries tri- and tetra- antennary N-glycans without/with fucose residues on Asn271. C)

Identification of mono-fucosylated bi-, tri- and tetra- antennary N-glycans on Asn271. We identified the diagnostic ion of $[GlcNAc+Gal+Fuc]^{1+}$ in the MS/MS spectra of Asn271 peptide with tri- and tetra-antennary N-glycans. This suggests the presence of antennary fucosylation on tri- and tetra- antennary N-glycans. However, we cannot rule out the presence of core-fucosylation on tri- and tetra-antennary N-glycans. Moreover, we didn't observe this diagnostic ion in the MS/MS spectrum of Asn271 peptide with bi-antennary N-glycan. It implies that the bi-antennary N-glycan on Asn271 may be principally core-fucosylated.

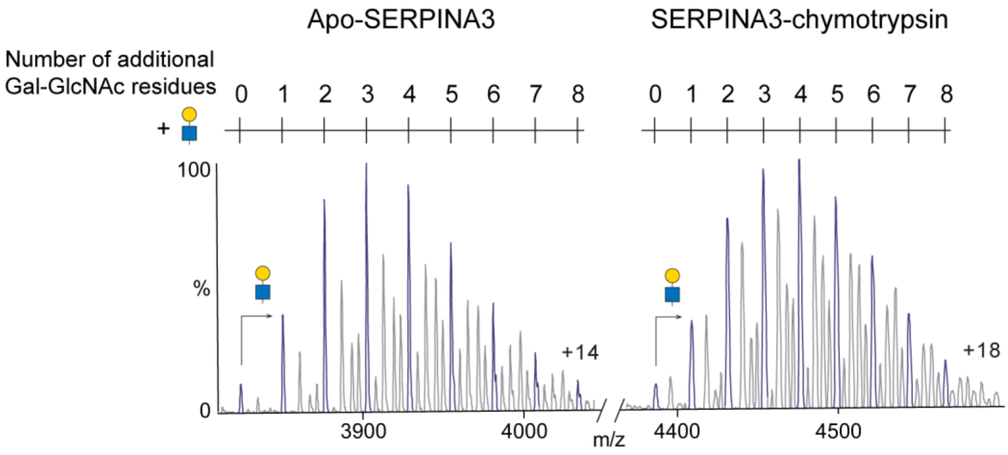

Figure S18. Annotation of N-glycan branching on apo-SERPINA3 and SERPINA3-chymotrypsin complexes.

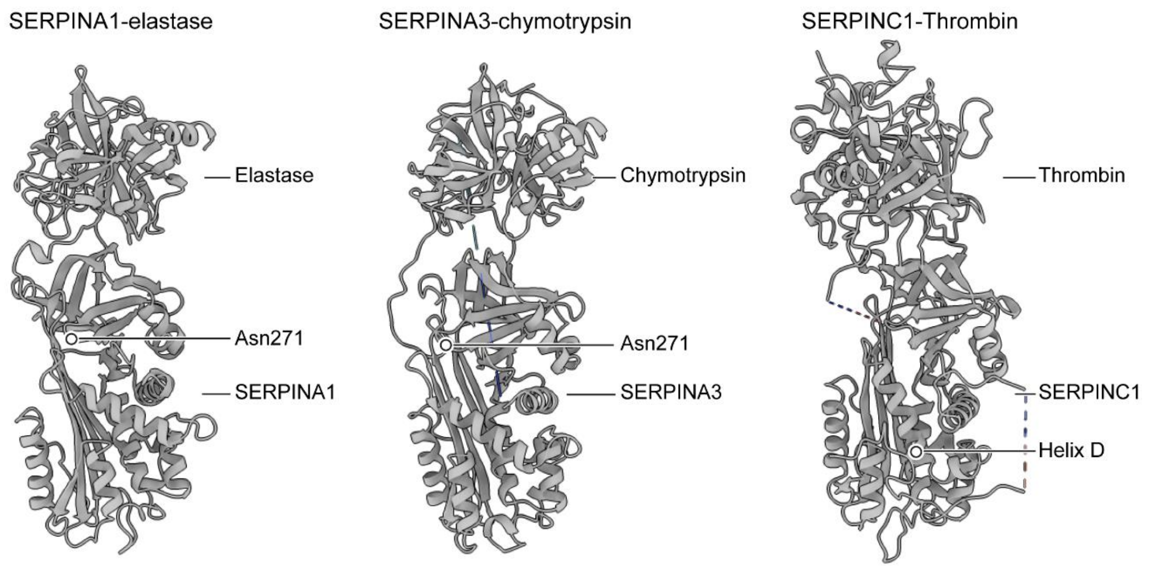

Figure S19. Structures of SERPINA1-elastase, SERPINA3-chymotrypsin, and SERPINC1-thrombin. The structures of SERPINA1-elastase and SERPINA3-chymotrypsin complexes were modelled using SERPINA1 (PDB: 3NE4), elastase (PDB: 1BMA), SERPINA3 (PDB: 6HGE) and chymotrypsin (PDB: 4CHA) using SERPINA1-trypsin complex (PDB: 1OPH) as a template using UCSF Chimera program. The structure of SERPINC1-thrombin was extracted from Thrombin-SERPINC1-heparin complex (PDB: 1TB6). The Asn271 in SERPINA1 and SERPINA3, and the helix D in SERPINC1 are highlighted, respectively.

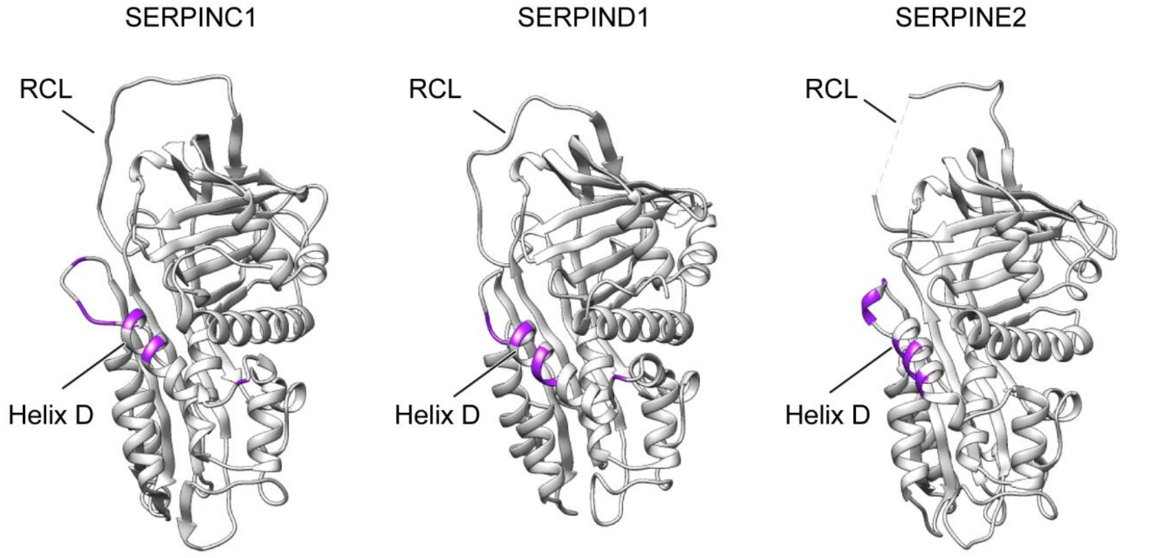

Figure S20. Structures of SERPINC1 (PDB:1OPH), SERPIND1 (PDB: 1JMJ) and SERPINE2 (PDB: 4DY0). The positively charged amino acids in helix D are highlighted in pink.

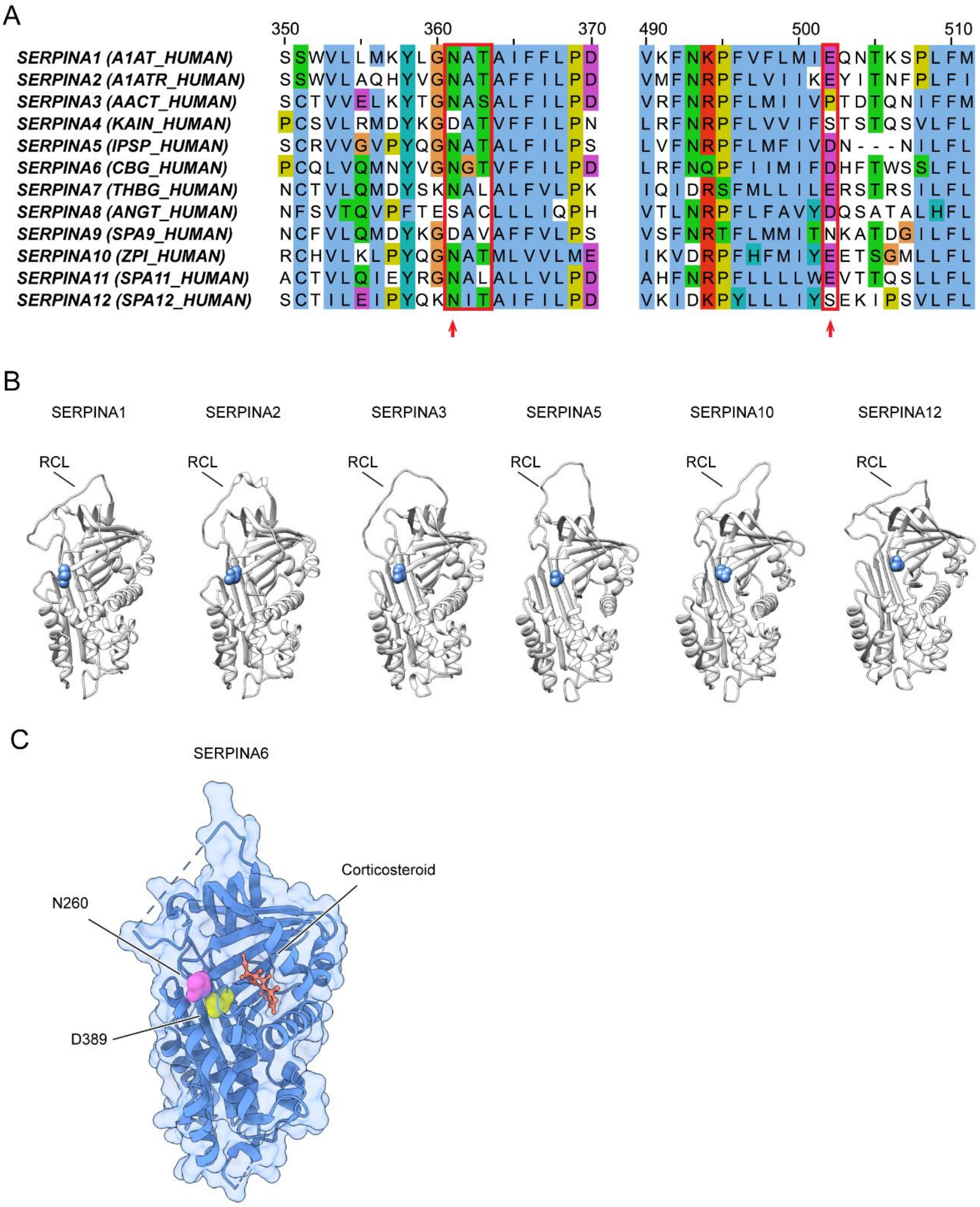

Figure S21. Structural analysis of SERPINs. A) Sequence alignment of the twelve members in SERPIN clade A. The sequence alignment was performed using Jalview program (B) Structures of SERPINA1, A2, A3, A5, A6 and A12. The N-glycosylation sites corresponding to N271 in SERPINA1/A3 are highlighted in blue. The SERPIN structures were retrieved from AlphaFold protein structural database. C) Structure of SERPINA6 (PDB: 2V95). The N260 (corresponding to N271 in SERPINA1/A3) is highlighted in pink. The D389 (corresponding to E400 in SERPINA1) is highlighted in yellow. The ligand, corticosteroid is in orange.

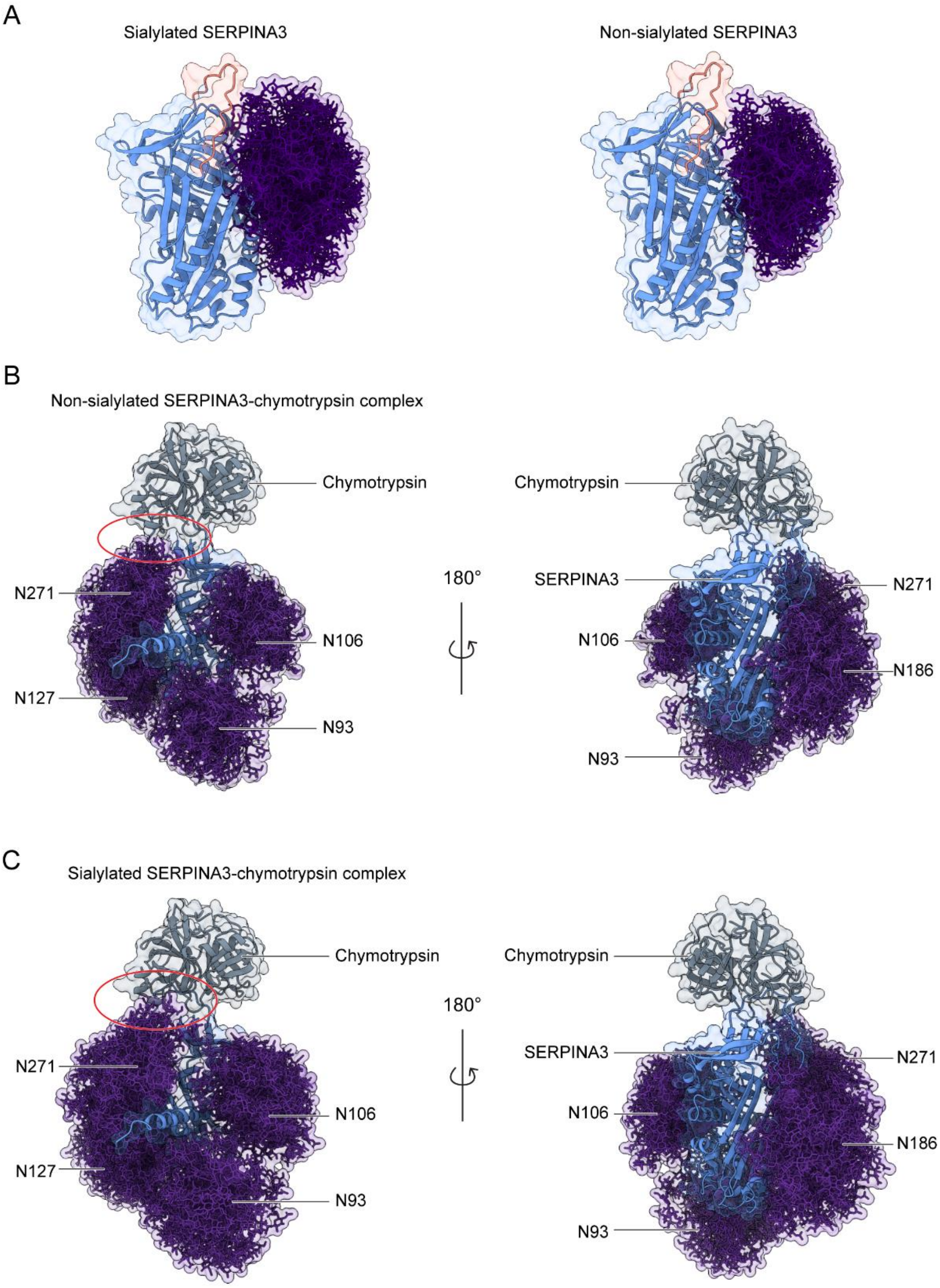

Figure S22. Structural analysis of glycosylated SERPINA3-chymotrypsin complexes. A) SERPINA3 with possible conformers of sialylated bi-antennary N-glycan and non-sialylated bi-antennary N-glycan on Asn271. The SERPINA3-chymotrypsin complexes with possible conformers of non-sialylated and sialylated bi-antennary N-glycans are shown in panel B and C, respectively. The red circles highlight that some N-glycan conformers on Asn271 are proximal to chymotrypsin.

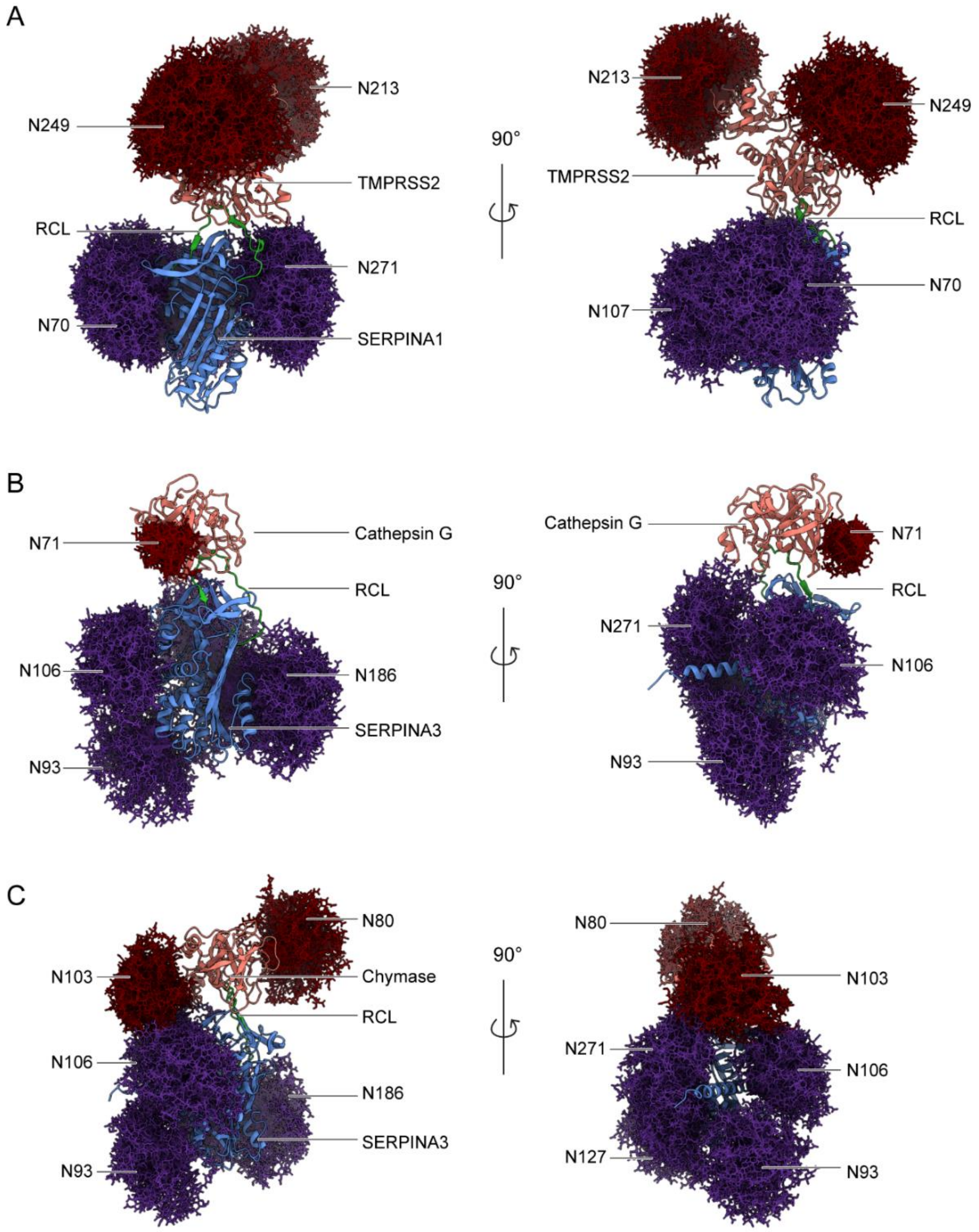

Figure S23. Modelling of the interactions between SERPINA1-TMPRSS2, SERPINA3-Cathepsin G and SERPINA3-Chymase complexes with all possible conformers of the sialylated bi-antennary N-glycans (panels A, B and C, respectively). Sialylated bi-antennary N-glycans were modelled at Asn70, Asn107 and Asn271 in SERPINA1, and Asn93, Asn106, Asn127, Asn186 and Asn271 in SERPINA3. According to previous biochemical studies, sialylated bi-antennary N-glycans were modelled at Asn213 and Asn219 in TMPRSS2 extracellular domain [15], and Asn80 and Asn103 in Chymase [16]. A small M2 glycan (Manα1-6Manβ1-4GlcNAcβ1-4GlcNAcβ) was modelled at Asn71 in Cathepsin G [17]. The N-glycans on TMPRSS2, Cathepsin G and Chymase are highlighted in dark red. The N-glycans on SERPINA1 and SERPINA3 are labelled in dark purple. The RCLs in SERPINA1 (Gly368 to Lys392) and SERPINA3 (Gly369 to Arg394) are highlighted in green in all models.

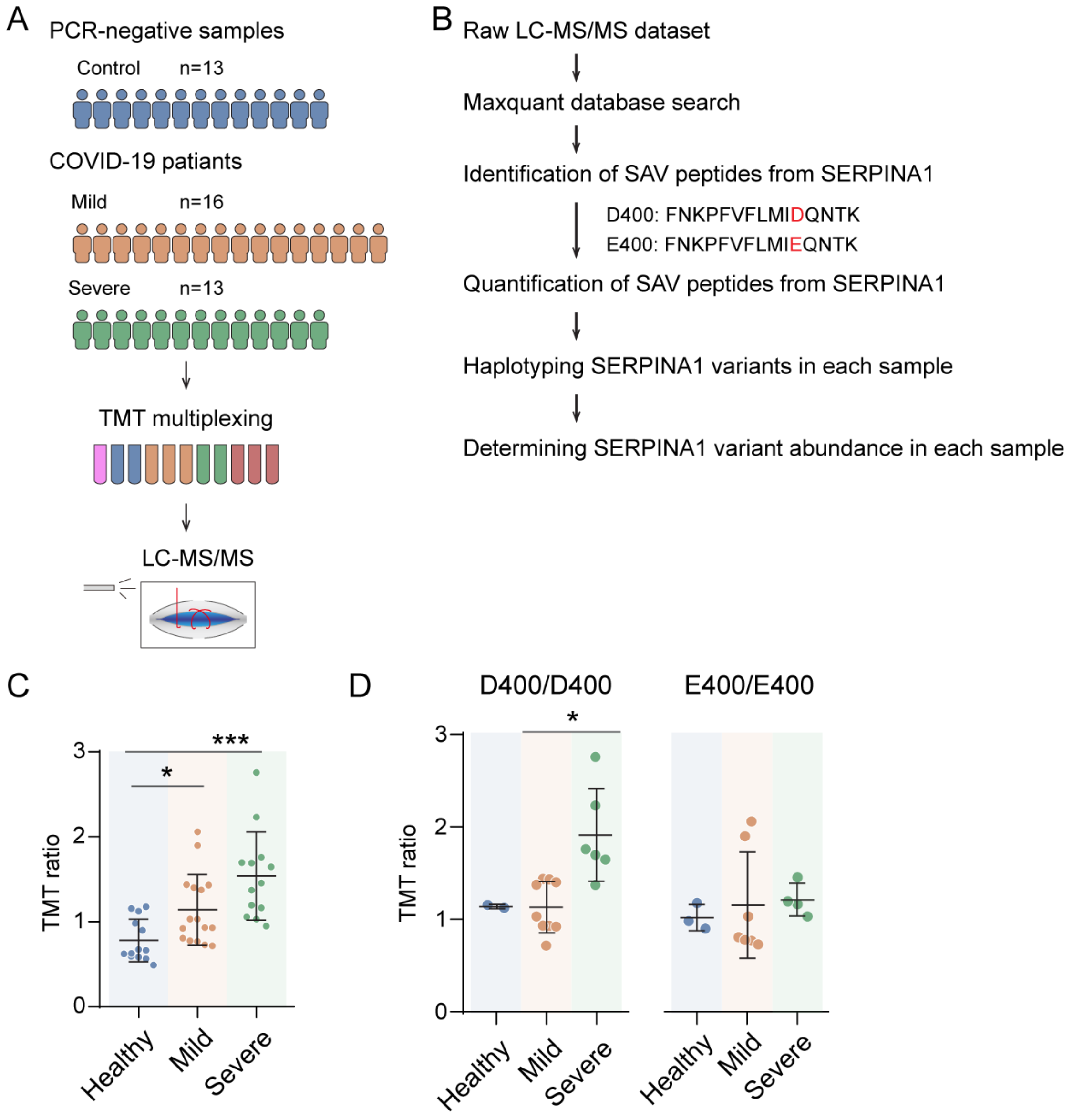

Figure S24. Analysis of SERPINA1 single amino acid variants (SAVs) in COVID-19. A) Overview of plasma sample information and proteomics workflow of COVID-19 patients, including 16 individuals with mild symptoms and 13 individuals with severe symptoms. Analysis of these plasma samples was performed by another research group using trypsin digestion, labelling with TMT 11-plex, fractionation and analysis with LC-MS/MS and reported elsewhere [13]. B) SAV analysis of a COVID-19 proteomics dataset. Briefly, we processed the LC-MS/MS raw data with Maxquant using a tailored human plasma protein database with SAV information. We identified SAV peptides from SERPINA1 (E400 and D400) and quantified their abundances. We performed phasing of E400 and D400 based on the relative abundances (TMT ratio) of the corresponding peptides in each sample. Detailed data processing steps are described in the Supplementary Methods. C) Relative abundances of SERPINA1 in healthy, mild and severe groups. Means and standard deviations of three groups were plotted (dots represent data from each sample). Nonparametric Mann-Whitney tests were performed to calculate p value (*, $p<0.05$; ***, $p<0.001$). Bonferroni correction was applied for post hoc analysis. D) Relative abundances of

SERPINA1$^{D400/D400}$ and SERPINA1$^{E400/E400}$ variants in healthy, mild and severe groups. Plots represent the means and standard deviations of three groups with dots representing data from each individual. Nonparametric Mann-Whitney tests were performed to calculate p value (*, $p<0.05$). Bonferroni correction was performed for post hoc analysis.

Supplemental references

1. Chen, S., Wu, D., Robinson, C. V, and Struwe, W.B. (2021). Native Mass Spectrometry Meets Glycomics: Resolving Structural Detail and Occupancy of Glycans on Intact Glycoproteins. Anal. Chem. *93*, 10435–10443. 10.1021/acs.analchem.1c01460.

2. Cox, J., Neuhauser, N., Michalski, A., Scheltema, R.A., Olsen, J. V, and Mann, M. (2011). Andromeda: a peptide search engine integrated into the MaxQuant environment. J. Proteome Res. *10*, 1794–1805. 10.1021/pr101065j.

3. Liu, M.-Q., Zeng, W.-F., Fang, P., Cao, W.-Q., Liu, C., Yan, G.-Q., Zhang, Y., Peng, C., Wu, J.-Q., Zhang, X.-J., et al. (2017). pGlyco 2.0 enables precision N-glycoproteomics with comprehensive quality control and one-step mass spectrometry for intact glycopeptide identification. Nat. Commun. *8*, 438. 10.1038/s41467-017-00535-2.

4. Pettersen, E.F., Goddard, T.D., Huang, C.C., Couch, G.S., Greenblatt, D.M., Meng, E.C., and Ferrin, T.E. (2004). UCSF Chimera--a visualization system for exploratory research and analysis. J. Comput. Chem. *25*, 1605–1612. 10.1002/jcc.20084.

5. Waterhouse, A.M., Procter, J.B., Martin, D.M.A., Clamp, M., and Barton, G.J. (2009). Jalview Version 2--a multiple sequence alignment editor and analysis workbench. Bioinformatics *25*, 1189–1191. 10.1093/bioinformatics/btp033.

6. Notredame, C., Higgins, D.G., and Heringa, J. (2000). T-coffee: A novel method for fast and accurate multiple sequence alignment. J. Mol. Biol. *302*, 205–217. 10.1006/jmbi.2000.4042.

7. Jo, S., Kim, T., Iyer, V.G., and Im, W. (2008). CHARMM-GUI: A web-based graphical user interface for CHARMM. J. Comput. Chem. *29*, 1859–1865. 10.1002/jcc.20945.

8. Lee, J., Cheng, X., Swails, J.M., Yeom, M.S., Eastman, P.K., Lemkul, J.A., Wei, S., Buckner, J., Jeong, J.C., Qi, Y., et al. (2016). CHARMM-GUI Input Generator for NAMD, GROMACS, AMBER, OpenMM, and CHARMM/OpenMM Simulations Using the CHARMM36 Additive Force Field. J. Chem. Theory Comput. *12*, 405–413. 10.1021/acs.jctc.5b00935.

9. Park, S.J., Lee, J., Qi, Y., Kern, N.R., Lee, H.S., Jo, S., Joung, I., Joo, K., Lee, J., and Im, W. (2019). CHARMM-GUI Glycan Modeler for modeling and simulation of carbohydrates and

glycoconjugates. Glycobiology *29*, 320–331. 10.1093/glycob/cwz003.

10. Abraham, M.J., Murtola, T., Schulz, R., Páll, S., Smith, J.C., Hess, B., and Lindahl, E. (2015). GROMACS: High performance molecular simulations through multi-level parallelism from laptops to supercomputers. SoftwareX *1–2*, 19–25. 10.1016/j.softx.2015.06.001.

11. Humphrey, W., Dalke, A., and Schulten, K. (1996). VMD: Visual molecular dynamics. J. Mol. Graph. *14*, 33–38. 10.1016/0263-7855(96)00018-5.

12. Gecht, M., von Bülow, S., Penet, C., Hummer, G., Hanus, C., and Sikora, M. (2021). GlycoSHIELD: a versatile pipeline to assess glycan impact on protein structures. Preprint at bioRxiv, 2021.08.04.455134. 10.1101/2021.08.04.455134.

13. Shu, T., Ning, W., Wu, D., Xu, J., Han, Q., Huang, M., Zou, X., Yang, Q., Yuan, Y., Bie, Y., et al. (2020). Plasma Proteomics Identify Biomarkers and Pathogenesis of COVID-19. Immunity *53*, 1108-1122.e5. 10.1016/j.immuni.2020.10.008.

14. Lössl, P., Snijder, J., and Heck, A.J.R. (2014). Boundaries of Mass Resolution in Native Mass Spectrometry. J. Am. Soc. Mass Spectrom. *25*, 906–917. 10.1007/s13361-014-0874-3.

15. Chen, Y.-W., Lee, M.-S., Lucht, A., Chou, F.-P., Huang, W., Havighurst, T.C., Kim, K., Wang, J.-K., Antalis, T.M., Johnson, M.D., et al. (2010). TMPRSS2, a Serine Protease Expressed in the Prostate on the Apical Surface of Luminal Epithelial Cells and Released into Semen in Prostasomes, Is Misregulated in Prostate Cancer Cells. Am. J. Pathol. *176*, 2986–2996. 10.2353/ajpath.2010.090665.

16. Chen, R., Jiang, X., Sun, D., Han, G., Wang, F., Ye, M., Wang, L., and Zou, H. (2009). Glycoproteomics analysis of human liver tissue by combination of multiple enzyme digestion and hydrazide chemistry. J. Proteome Res. *8*, 651–661. 10.1021/pr8008012.

17. Loke, I., Packer, N., and Thaysen-Andersen, M. (2015). Complementary LC-MS/MS-Based N-Glycan, N-Glycopeptide, and Intact N-Glycoprotein Profiling Reveals Unconventional Asn71-Glycosylation of Human Neutrophil Cathepsin G. Biomolecules *5*, 1832–1854. 10.3390/biom5031832.
